# Impact of Oxygen and Sulfur Heteroatom Core Substitution on Catalyst Properties of Phenoxazines and Their Performance in Organocatalyzed Atom Transfer Radical Polymerization (O‐ATRP)

**DOI:** 10.1002/chem.202501179

**Published:** 2025-07-09

**Authors:** Jessica L. Lathrop, Brandon S. Portela, Robert S. Paton, Garret M. Miyake

**Affiliations:** ^1^ Department of Chemistry Colorado State University 200 W. Lake St. Fort Collins Colorado 80523 USA

**Keywords:** catalysis, photocatalysts, photochemistry, radical polymerizations, SuPRCat

## Abstract

Phenoxazines are a successful class of organic photoredox catalysts (PCs) with tunable redox and photophysical properties. Originally, we aimed to realize more reducing phenoxazine PCs through heteroatom core substituted (HetCS) derivatives, while maintaining an efficiently oxidizing PC^·+^. However, core modification with thioether or ether functionality to a PC that exhibits photoinduced intramolecular charge transfer (CT) negligibly alters the singlet excited state reduction potential (*E*_S1_°*), while yielding a less oxidizing PC^·+^ (*E*_1/2_) (*E*_1/2 _= 0.50–0.64 V vs. SCE) compared to the noncore modified PC **1** (0.68 V vs. SCE). Photophysical characterization of HetCS PCs revealed that increasing electron density on the core of a CT exhibiting PC stabilizes the emissive state and PC^·+^, resulting in a relatively unchanged *E*_S1_°* compared to PC **1**. In contrast, modifying the core of a PC that does not exhibit CT yields a highly reducing *E*_S1_°* (PC **3** = −2.48 V vs. SCE) compared to its CT equivalent (PC **1d** = −1.68 V vs. SCE). The impact of PC property on photocatalytic ability was evaluated through organocatalyzed atom transfer radical polymerization (O‐ATRP). HetCS PCs were able to yield poly(methyl methacrylate) with low dispersity and moderate targeted molecular weight as evaluated by initiator efficiency (I*) in DMAc (*Ð* = 1.20–1.26; I* = 47–57%). Ultimately, this work provides insight into how phenoxazine PC properties are altered through structural modification, which can inform future PC design.

## Graphical abstract

Phenoxazine core structural modification reveals the impact of charge transfer on stronger excited state reduction potentials. We observed that modifying the core of a photocatalyst that does not exhibit charge transfer yields a highly reducing singlet excited state reduction potential compared to a charge transfer equivalent.

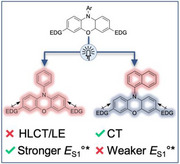

## 1 Introduction

Inspired by nature's ability to harvest solar energy and convert it into chemical energy, photoredox catalysis has emerged as a powerful approach for driving synthetic transformations through harnessing the power of light.^[^ ^1^ ^]^ Advancements within photoredox catalysis have revealed the important structure‐property‐performance relationships of photoredox catalysts (PCs). Metal‐based PCs, such as ruthenium and iridium complexes, are among the most employed PCs in photoredox catalysis due to efficient light absorption, reversible redox properties, high quantum yield of intersystem crossing (*ɸ*_ISC_), and long excited state lifetimes.^[^ ^2^, ^3^ ^]^ While ruthenium and iridium PCs are successful, there are some limitations to their use. PCs are usually difficult to separate from polymeric materials, and metal catalyst residue can limit material applications in electronics or biological systems^.[^ ^4^, ^5^ ^]^ Moreover, ruthenium and iridium are costly to obtain both financially and environmentally. Therefore, organic PCs are sought after as more sustainable alternatives.^[^ ^6^ ^]^ While some organic PCs have properties that rival metal PCs, such as high molar absorptivity (*ε*_max,abs_)^[^ ^7^, ^8^, ^9^ ^]^ and high *ɸ*_ISC_,^[^ ^10^ ^]^ further elucidation of structure‐property relationships will aid in PC design for improved catalytic ability, such as increasing reactivity and selectivity through precisely tuned redox potentials.

**Figure 1:**
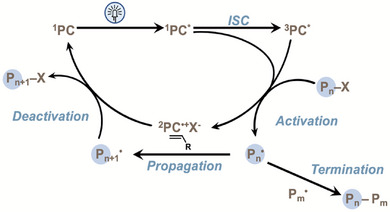
Catalytic cycle for O‐ATRP proceeding through an oxidative quenching pathway.

Among the diverse applications of organic PCs, organocatalyzed atom transfer radical polymerization (O‐ATRP) is a polymerization method that utilizes organic PCs and visible light to produce well‐defined polymers.^[^ ^4^, ^11^, ^12^, ^13^, ^14^, ^15^ ^]^ In previous work, we sought to design organic PCs that exhibited photophysical and redox properties that would lead to improved polymerization control, defined by producing polymers possessing targeted molecular weight as evaluated by initiator efficiency (I*) approaching 100% and low dispersity (1.0 < *Ð* ≤ 1.5).^[^ ^4^ ^]^ In particular, the ability of the PC to efficiently catalyze activation and deactivation steps is crucial to keep radical concentrations low and maintain polymerization control through minimizing bimolecular radical termination events (Figure 1).^[^ ^4^ ^]^ Upon photoexcitation, PCs can perform single electron transfer (SET) from either the singlet or triplet excited state to activate the initiator or dormant polymer chain‐end (Figure 1). While PCs that exhibit near‐unity *ɸ*_ISC_ have increased access to the triplet excited state and allow for efficient O‐ATRP at low catalyst loadings, previous studies have shown that the singlet excited state can also contribute to catalysis due to high concentrations of initiator and PC in O‐ATRP.^[^ ^16^, ^17^ ^]^ As such, experimental investigation of the triplet excited state is beyond the scope of this work and reserved for future exploration of HetCS PCs. To mediate activation and deactivation steps in O‐ATRP, the PC must have an excited state reduction potential (*E*°*) more reducing (*E*°(P_n_‐Br/P_n_‐Br^·−^) ∼ −0.8–0.6 V vs. SCE) than common alkyl bromide ATRP initiators or dormant polymer chain‐ends as well as a more oxidizing PC radical cation (PC^·+^) oxidation potential (*E*_1/2_ or *E*°(^2^PC^·+^/^1 ^PC) > −0.8 V vs. SCE) than the propagating polymer radical to reinstall the bromine chain‐end.^[^ ^18^, ^19^ ^]^ However, synthetically tuning the PC core to afford a highly reducing singlet excited state reduction potential (*E*_S1_°*) and a sufficiently oxidizing PC^·+^ presents challenges, as these properties are interrelated. Specifically, targeting a highly oxidizing PC^·+^ typically leads to a less reducing *E*_S1_°*, while a more reducing *E*_S1_°* typically lends a less oxidizing PC^·+^ (**Eqn. 1**).^[^ ^6^, ^9^ ^]^ One approach to circumvent the challenge of yielding highly oxidizing and reducing PCs is to destabilize the lowest energy singlet excited state (*E*_S1_) through structural modification of a PC that already possesses a high *E*_1/2_.

(1) $${E_{S1}}^{\circ *}=E_{1/2}-E_{S1}$$

In addition to redox properties, photophysical characteristics impact the catalytic ability of the PC. Synthetically tuning a PC to afford visible light absorption (*λ*_max,abs_) is desirable not only to minimize unwanted side reactions through selective excitation of the PC but also to reduce the need for specialized equipment capable of safely generating high‐energy UV light.^[^ ^20^, ^21^ ^]^ Additionally, PCs that possess high *ε*_max,abs_ can more readily absorb light to access the reactive excited state (^n^PC*). The character of the lowest energy excited state, such as charge transfer (CT), is also relevant for achieving control in O‐ATRP. PCs that exhibit CT character typically offer enhanced control compared to those that have locally excited (LE) character. CT is the relocalization of electron density from one area of the molecule to another after photoexcitation, while LE character PCs show electron density remaining localized on the core.^[^ ^11^, ^22^ ^]^ Additionally, PCs can exhibit hybridized local and charge transfer (HLCT) character, which is characterized by having both LE and CT contributions arising from the hybridization of LE and CT excited states.^[^ ^17^, ^23^, ^24^ ^]^ HLCT states displaying minimal solvatochromatic shifts and narrow emissive spectra are classified as LE‐dominated due to limited CT contribution.^[^ ^25^, ^26^ ^]^ Alternatively, HLCT states exhibiting high solvatochromatic shifts and broad emission spectrums are referred to as CT‐dominated due to reduced LE contribution.^[^ ^27^ ^]^ CT is hypothesized to facilitate faster electron transfer by inducing charge separation within the molecule, which reduces unproductive recombination between the excited electron and hole.^[^ ^11^, ^28^, ^29^ ^]^ We have explored numerous PC families for application within O‐ATRP such as perylenes,^[^ ^30^ ^]^ dihydroacridines,^[^ ^9^ ^]^ dihydrophenazines,^[^ ^7^, ^12^ ^]^ phenothiazines,^[^ ^14^ ^]^ and phenoxazines.^[^ ^8^, ^11^ ^]^ Here, this work focuses on the derivatization of phenoxazines due to their favorable photophysical and redox properties within O‐ATRP. In particular, phenoxazine PCs commonly exhibit a high *E*_1/2_ (0.52–0.72 V vs. SCE) necessary for efficient deactivation, while also possessing favorable photophysical properties such as absorption profiles tailing into the visible light region (*λ*_max,abs_ = 345–411 nm) and high *ε*_max,abs_ (6850 M^−1^cm^−1^–37700 M^−1^cm^−1^).^[^ ^8^ ^]^ However, phenoxazines are typically less reducing (*E*_S1_°* = −1.67 to −2.03 V vs. SCE)^[^ ^8^, ^11^ ^]^ than *N,N*‐dihydrophenazines (*E*_S1_°* = −1.64 to −2.50 V vs. SCE).^[^ ^8^, ^12^ ^]^ This observation motivated us to pursue a more reducing *E*_S1_°* through phenoxazine core modification.

**Figure 2:**
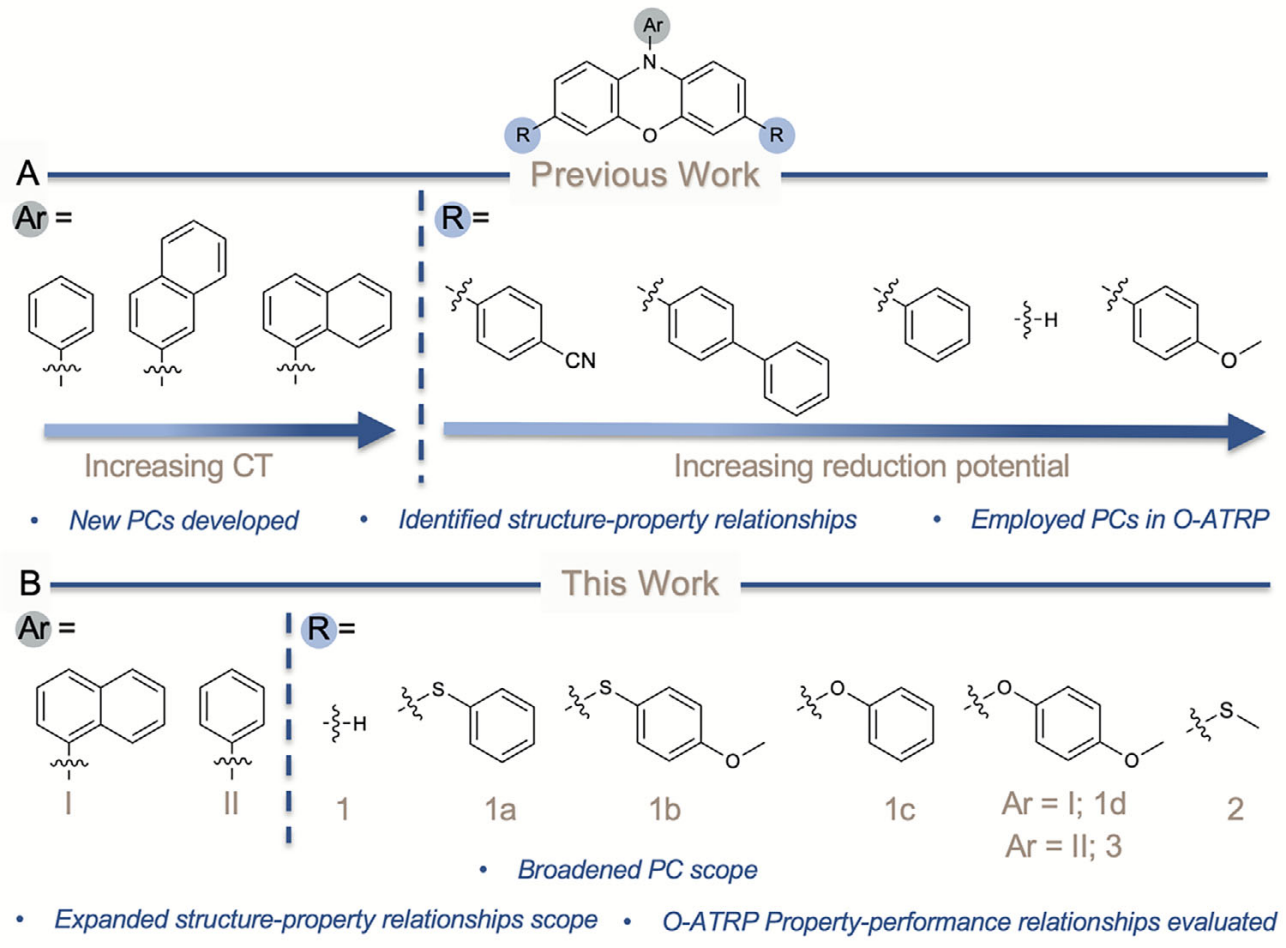
A) Previously reported phenoxazine PCs and their photophysical trends. B) Summary of PCs investigated in this work.

The major points for structural modification of phenoxazines that are synthetically easily accessible include the *N*‐substituent and 3 and 7 core positions (Figure 2). For substitutions on nitrogen, 1‐naphthalene results in a lower energy singlet excited state compared to phenyl or 2‐naphthalene, where shorter donor‐acceptor distances from the core increase the driving force for CT character and lead to more stabilized excited state species (Figure 2A).^[^ ^28^ ^]^ Installing aryl substituents on the 3 and 7 positions of the core stabilizes the π* orbital through extended conjugation, thereby resulting in red‐shifted *λ*_max,abs_ and increased *ε*_max,abs_ as core conjugation increases. Furthermore, incorporating an electron‐rich substituent, such as 4‐methoxyphenyl, onto the 3 and 7 core positions of *N*‐2‐naphthalene phenoxazine resulted in a more reducing *E*_S1_°* (*E*_S1,exp_°* = −1.81 V vs. SCE) whereas an electron‐deficient substituent, such as 4‐cyanophenyl, led to a less reducing *E*_S1_°* (*E*_S1,exp_°* = −1.75 V vs. SCE) (Figure 2A).^[^ ^8^ ^]^ Core modification with electron‐donating substituents increases electron density on the core, resulting in a more stabilized PC^·+^ and a more reducing excited state PC.

Building on these design principles, we aimed to modify the core of *N*‐1‐naphthalene phenoxazine with electron‐donating oxygen and sulfur heteroatom aryl substituents to improve excited‐state reduction potentials, while preserving key properties such as sufficient *E*_1/2_ for deactivation, visible‐light absorption, and CT character. We experimentally and computationally investigated the redox and photophysical properties of six different heteroatom derivatives to gain insight into PC structure‐property relationships, with PCs **2** and **3** selectively characterized to further test the hypotheses developed within this work (Figure 2B). We found that HetCS on CT character PCs result in a relatively unchanged *E*_S1_°* compared to PC **1** due to stabilization of PC^·+^ and the singlet emissive state, lowering both *E*_1/2_ and *E*_S1_, respectively (**Eqn 1**). However, HetCS on a LE‐dominated HLCT character PC core (PC **3**) resulted in a stabilized PC^·+^ and a destabilized singlet emissive state, lowering *E*_1/2_ and increasing *E*_S1_, thereby resulting in a less oxidizing but more reducing PC compared to the CT analog. Furthermore, PCs **1a**, **1b**, **1c**, **1d**, and **3** were employed in O‐ATRP to investigate how the catalytic properties influence polymerization performance. PCs **1a**, **1b**, **1c**, and **1d** were able to synthesize poly(methyl methacrylate) (PMMA) with relatively low dispersity in DMAc (*Ð* = 1.20–1.26; I* = 47–57%). This work reveals structure‐property relationships and the interplay between CT character and electronics that will inform future photocatalyst modifications for applications in both polymerization and small molecule transformations.

## 2 Results and Discussion

### 2.1 Redox Properties

**Figure 3:**
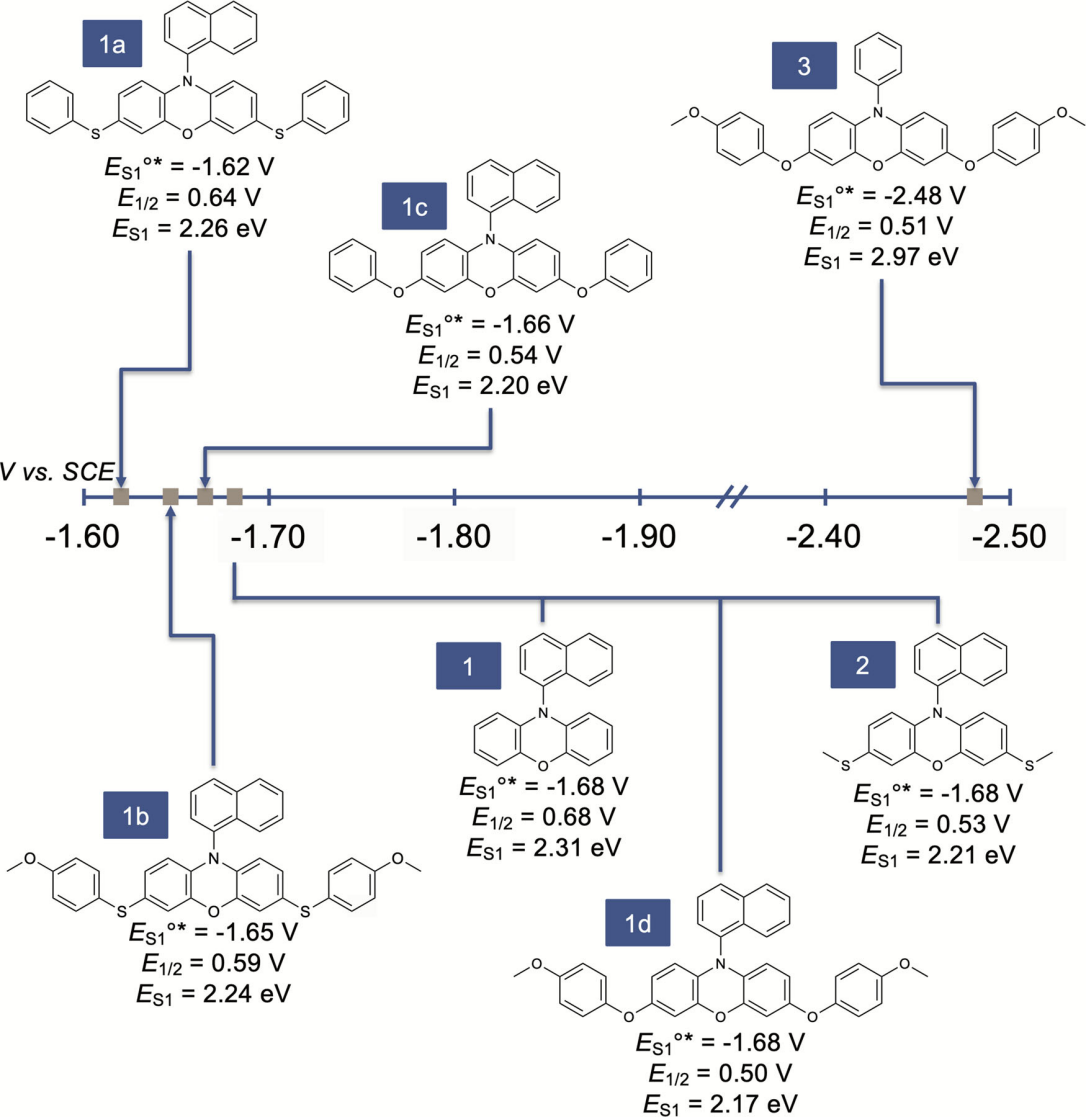
PC structures and their associated redox properties.

Previously reported phenoxazine structure‐property relationships demonstrated that incorporating electron‐donating aryl groups onto the phenoxazine core led to a more reducing *E*_S1_°* and less oxidizing *E*_1/2_ compared to core modification with electron‐withdrawing aryl groups.^[^ ^8^ ^]^ In pursuit of accessing more reducing phenoxazine PCs, we functionalized the PC core with electron‐donating oxygen and sulfur heteroatoms of varying aryl electronics, with phenyl serving as electron‐neutral and 4‐methoxyphenyl as electron‐rich. In comparison to PC **1,** we hypothesized that HetCS PCs would possess a less oxidizing *E*_1/2_ and a more reducing *E*_S1_°* through PC^·+^ stabilization and *E*_S1_ destabilization due to the core possessing more electron density. When the redox properties were measured, HetCS PCs exhibited less oxidizing *E*_1/2_ (*E*_1/2 _= 0.50 V to 0.64 V vs. SCE) compared to PC **1** (*E*_1/2 _= 0.68 V vs. SCE), while maintaining a relatively unaffected *E*_S1_°*, excluding PC **3** (Figure 3, Table 1). As PC **1** possesses only carbon core substituents, the less oxidizing *E*_1/2_ observed in HetCS PCs can most likely be attributed to enhanced electron donation onto the core. We observed an increased stabilization of PC^·+^ going from the sulfur substituted PCs **1a** (*E*_1/2 _= 0.64 V vs. SCE) and **1b** (*E*_1/2 _= 0.59 V vs. SCE) to oxygen substituted PCs **1c** (*E*_1/2 _= 0.54 V vs. SCE) and **1d** (*E*_1/2_ = 0.50 V vs. SCE), with a trend of decreasing oxidation potential from PC **1a** to PC **1d**. These data indicate that the oxygen‐based substituents stabilize PC^·+^ more than sulfur‐based substituents, which we propose might be due to better atomic overlap of oxygen with carbon in comparison to larger sulfur atoms.^[^ ^31^ ^]^

**Table 1:** Photophysical and redox properties for investigated PCs.

PC	*λ*_max,abs_ [nm]^[^ ^a^ ^]^	*λ*_max,em_ [nm]^[^ ^b^ ^]^	*ε*_max,abs_ [M^−1^cm^−1^]^[^ ^c^ ^]^	*E*_S1,exp_ (eV)^[^ ^d^ ^]^	*E*_1/2_ [^2^PC^·+^/^1^PC] [V vs. SCE]^[^ ^e^ ^]^	E°*_S1,exp_ [^2^PC^·+^/^1^PC*] [V vs. SCE]^[^ ^f^ ^]^	*E*_T1,calc[eV]_^[^ ^g^ ^]^
**1**	318	536	8040	2.31	0.68	−1.68	2.64
**1a**	347	549	15000	2.26	0.64	−1.62	2.61
**1b**	346	554	13000	2.24	0.59	−1.65	2.48
**1c**	323	563	9500	2.20	0.54	−1.66	2.48
**1d**	320	572	10000	2.17	0.50	−1.68	2.42
**2**	340	560	−	2.21	0.53	−1.68	2.44
**3**	319	417	9800	2.97	0.51	−2.48	2.74

^[a]^ Wavelength of maximum absorption measured in DMAc using UV/Vis.

^[b]^ Wavelength of maximum fluorescence measured in DMAc using 20 nm above *λ*_max,abs_ of each PC as the excitation wavelength

^[c]^ Determined through Beer‐Lambert's Law.

^[d]^ *E*_S1,exp_ calculated through relationship of *E*_S1 _= 1240 / *λ*_max,em_.

^[e]^ Oxidation potential measured in DMAc with a 0.1 M NBu_4_PF_6_ electrolyte solution and Ag/AgNO_3_ reference electrode.

^[f]^ See **Eqn. 1** for *E*_S1_°* calculation.

^[g]^ Computed at the M06‐2X‐D3‐SMD^DMAc^/def2‐SVPD(H;C)def2‐TZVPD(O;S)//ωB97XD/6–31+G**‐SMD^DMAc^ level of theory.

While *E*_1/2_ followed previously established trends for all HetCS PCs, the *E*_S1_°* remained relatively unaffected for all PCs except PC **3**. In order to analyze *E*_S1_°*, we examined the *E*_S1_ trends within HetCS PCs. Originally, we hypothesized that increasing electron density onto an already electron‐rich core would result in a higher *E*_S1_ due to π* orbital destabilization. However, we observed that PCs **1a** through **2** demonstrated lower *E*_S1_ compared to PC **1**, with decreasing *E*_S1_ from PC **1a** (*E*_S1_ = 2.26 eV) to PC **1d** (*E*_S1_ = 2.17 eV) (Table 1). The lower *E*_S1_ suggests stabilization of the lowest energy singlet excited state, where PC **1d** has the most stable singlet excited state while PC **1a** has the least stable. Combining the *E*_S1_ and *E*_1/2_ trends, we observed that increasing electron density on the core correlates with a more stabilized singlet emissive state.

### 2.2 Photophysical Properties

**Figure 4:**
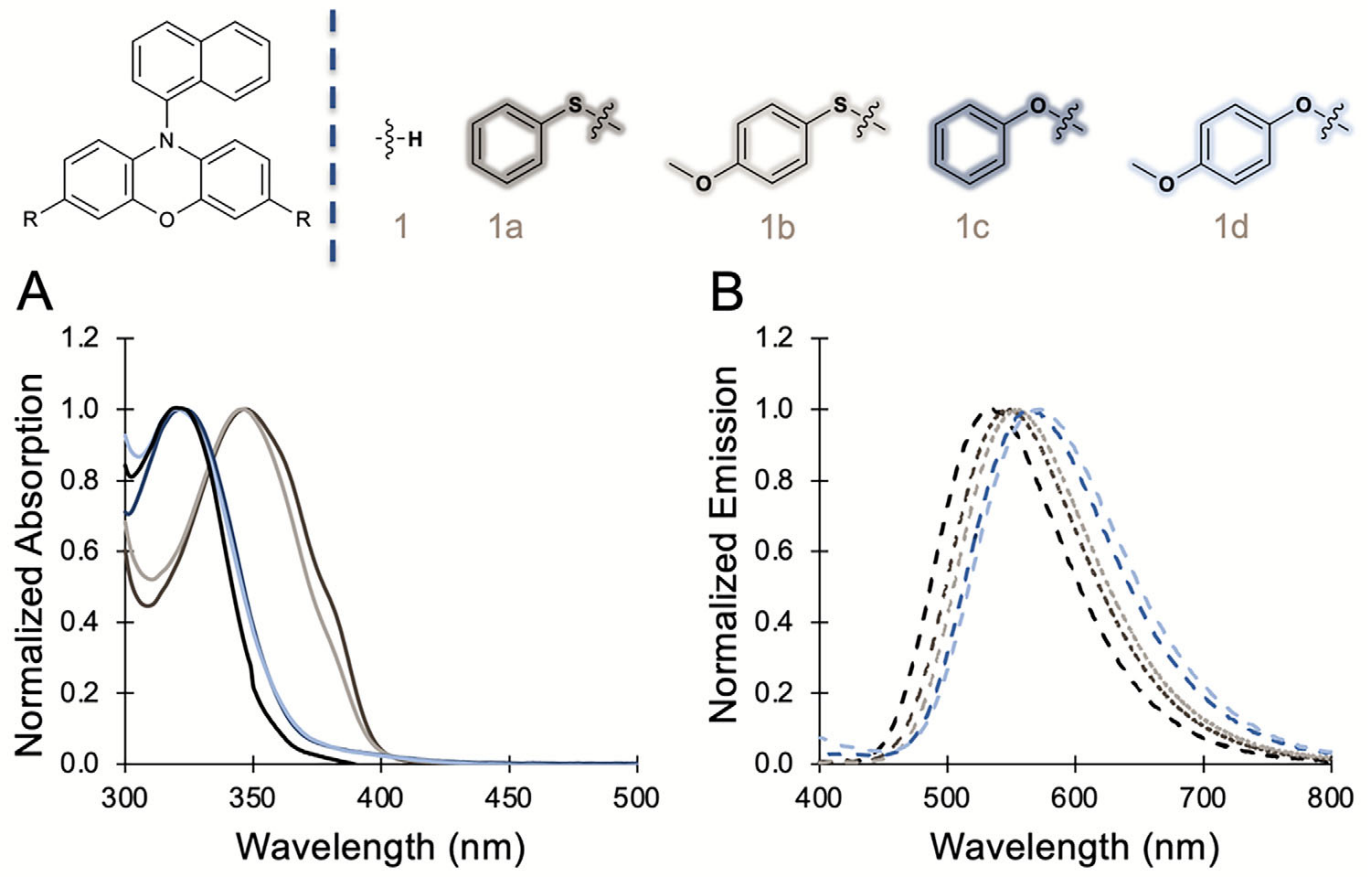
A) Overlaid absorption spectra for PCs **1**, **1a**, **1b**, **1c**, and **1d** obtained in DMAc. B) Overlaid emission spectra for PCs **1**, **1a**, **1b**, **1c**, and **1d** measured in DMAc.

Another motivation for designing efficient organic PCs is modifying the core with aryl substituents to red‐shift *λ*_max,abs_ and increase *ε*_max,abs_.^[^ ^8^ ^]^ We hypothesized that PC **1a**, **1b**, **1c**, and **1d** would have a red‐shifted *λ*_max,abs_ compared to PC **1**. UV/Vis measurements, density functional theory (DFT), and time‐dependent density functional theory (TD‐DFT) were employed to investigate the impact of HetCS on λ_max,abs_ and the orbitals involved in photoexcitation. Notably, we observed a red‐shifted *λ*_max,abs_ for PCs **1a** and **1b,** while PCs **1c** and **1d** maintained similar absorption profiles as PC **1** (Table 1, Figure 4). This observation motivated us to computationally investigate the highest molecular orbital contribution involved in the photoexcitation process to elucidate further why oxygen and sulfur are impacting absorption differently. Computations were performed at the M06‐2X(D3)/def2‐SVPD(H;C)def2‐TZVPD(O;S)//ωB97XD/6–31+G(d,p) level of theory with a SMD solvation model for DMAc. Calculations used the Gaussian 16 rev. C01 package. Further details, references, and minor orbital contributions are given in the Supporting Information.

**Figure 5:**
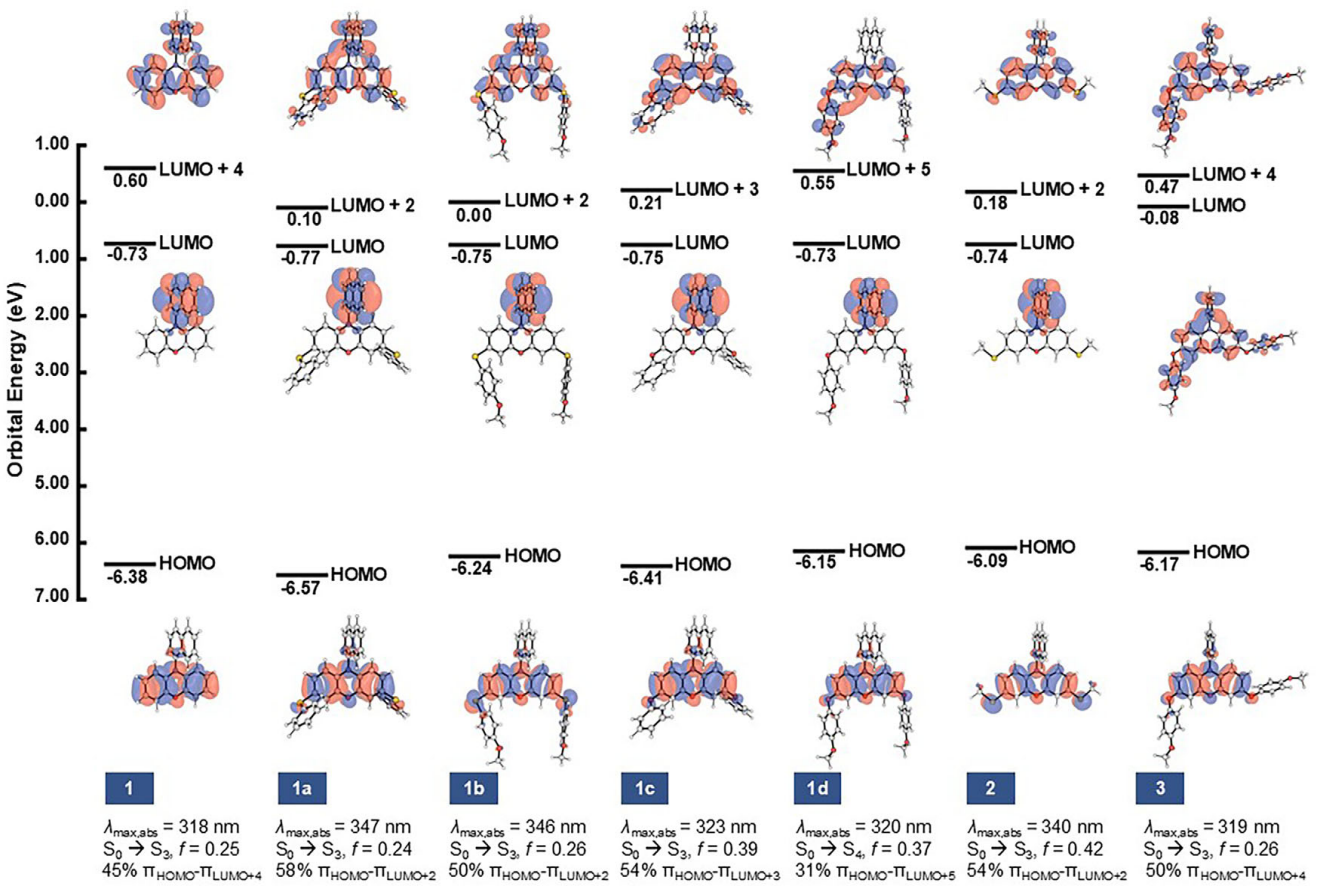
Computed orbital energies (in eV) and orbital percentage contributions of PCs **1**, **1a**, **1b**, **1c**, **1d**, **2**, and **3** at their corresponding *λ*_max,abs_.

PC **1** has the highest energy *λ*_max,abs_ (318 nm), which is predicted to be the S_o_ → S_3_ excitation with an oscillator strength (*f*) of 0.25 and with an orbital contribution of 45% from π_HOMO_‐π_LUMO+4_. As shown with PCs **1**, **1a**, **1b**, **1c**, and **1d**, the highest occupied molecular orbital (HOMO) is localized in the phenoxazine core, and the initial photoexcitation event involves the promotion of an electron residing in the π_HOMO_ orbital to a high‐lying π^*^ orbital of CT character (Figure 5). PC **1a** exhibits the lowest energy *λ*_max,abs_ (347 nm), which corresponds to the S_o_ → S_3_ excitation with *f *= 0.24 and with orbital contribution of 58% from the π_HOMO_‐π_LUMO+2_. For PC **1b**, the *λ*_max,abs_ (346 nm) is predicted to be the S_o_ → S_3_ excitation with *f *= 0.26 and with orbital contributions of 50% from the π_HOMO_‐π_LUMO+2_. For the electron‐neutral oxygen HetCS PC **1c**, the *λ*_max,abs_ (323 nm) is predicted to be the S_o_ → S_3_ excitation with *f *= 0.39 and with orbital contribution of 54% π_HOMO_‐π_LUMO+3_. For PC **1d**, the *λ*_max,abs_ (320 nm) is predicted to be the S_o_ → S_4_ excitation with *f *= 0.37 and with orbital contributions of 31% π_HOMO_‐π_LUMO+5._ From our computational analysis, we found that the primary orbital responsible for photoexcitation retains its electron density on the core for PCs **1c** and **1d,** while PCs **1a** and **1b** exhibit charge density relocalization. Thus, *λ*_max,abs_ is influenced by inductive effects and core electronics in addition to extended conjugation. We reason that extending core conjugation and inductive effects may be stabilizing the initially excited state through delocalization of electron density, while electron donation onto the core may destabilize it, leading to opposing effects on *λ*_max,abs_. Based on the *E*_1/2_ trends, PCs **1c** and **1d** possess a more electron‐rich core in comparison to PCs **1a** and **1b**. Due to increased electron donation onto the core, PCs **1c** and **1d** could be combating the extended conjugation effect more compared to PCs **1a** and **1b**, therefore leading to a blue‐shift in *λ*_max,abs_.

To provide further support for the alternative hypothesis, PC **2** was synthesized with methanethiol on the 3 and 7 core positions to probe the impact of alkyl sulfur substitution on absorption relative to aryl sulfur substituents on PCs **1a** and **1b**. We hypothesized that PC **2** would exhibit a blue‐shifted *λ*_max,abs_ compared to PCs **1a** and **1b** due to the alkyl sulfur substituents, but a red‐shifted *λ*_max,abs_ compared to PCs **1c** and **1d** because of less electron donation onto the core. In support of this alternative hypothesis, PC **2** exhibited a blue‐shifted *λ*_max,abs_ (340 nm) compared to the aryl sulfur substituted PCs, but a more red‐shifted *λ*_max,abs_ relative to those with oxygen core modifications (Table 1). Computations show the electron‐rich aryl substituted PCs (PCs **1b** and **1d**) exhibited a higher energy HOMO (0.14 eV, 0.23 eV) in comparison to PC **1**. These data suggest that destabilization of the HOMO energy from electron donation onto the phenoxazine core is predominant over the stabilization through conjugation and inductive effects. However, for the electron‐neutral sulfur HetCS PC **1a**, the HOMO energy is stabilized by 0.19 eV, while the electron‐neutral oxygen HetCS PC **1c** HOMO energy remains effectively unchanged. These findings are attributed to reduced electron donation from the electron‐neutral sulfur core substituent, suggesting that stabilization through extended conjugation is more significant, leading to a decrease in HOMO energy. In contrast, the electron‐neutral oxygen core substituent exhibits greater electron donation to the phenoxazine core, counteracting stabilization through extended conjugation and resulting in a negligible change in HOMO energy.

The lowest energy singlet excited state was characterized to investigate the impact of HetCS on emissive states. PCs exhibit CT when electron density shifts from one location within the molecule to another in the excited state, while LE and HLCT character PCs exhibit unchanged or delocalized electron density across the molecule. Previously, we observed CT in phenoxazine PCs from the core to either the *N*‐aryl group or core substituents, depending on the identity of the substituent. Previous studies have shown that incorporating naphthalene as the *N*‐aryl group on a phenoxazine core promotes CT from the core to the naphthalene group, resulting in a radical cation‐like core and a radical anion‐like naphthalene moiety.^[^ ^28^ ^]^ We suggest that donating electron density onto the core of a PC with CT character might stabilize the radical cation‐like core, thereby resulting in a red‐shifted *λ*_max,em_, lower *E*_S1_, and ultimately, a less reducing *E*_S1_°*. Additionally, because LE and HLCT character PCs show electron density remaining on the core, we hypothesized that HetCS would destabilize π* orbitals and increase *E*_S1_, and consequently yield a more reducing PC in comparison to CT character PCs. To test this hypothesis, we were guided by previous literature to structurally tune PC **1d** into a PC with LE or HLCT character by replacing the naphthalene group with a phenyl group (PC **3**).^[^ ^28^ ^]^ We experimentally and computationally probed the character of the lowest energy singlet excited state to determine if PC **3** exhibits predominately CT, LE, or HLCT character.

**Figure 6:**
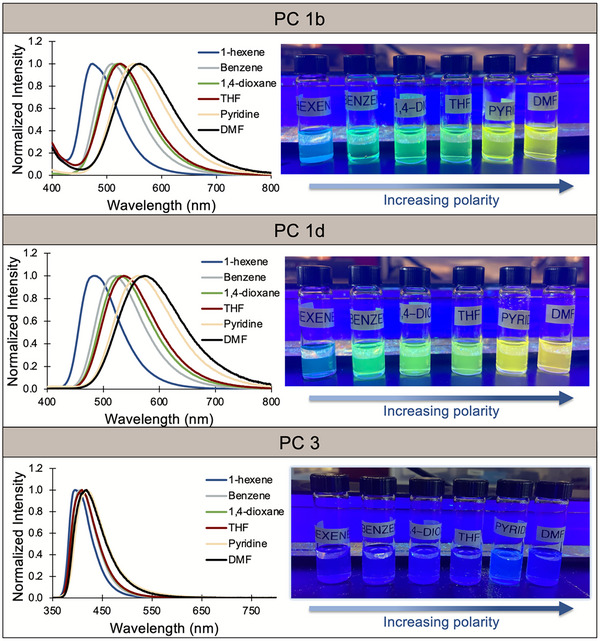
Overlaid emission spectra (left) and photographs (right) of PCs **1b**, **1d**, and **3** dissolved in varying polarity solvents and irradiated with UV light.

Experimentally, solvatochromism supports if a molecule is of CT character in the lowest energy singlet excited state, as evidenced by a red‐shift in *λ*_max,em_ in increasingly polar solvents due to CT character stabilization.^[^ ^32^, ^33^ ^]^ Due to similar core substituents, the solvatochromic nature of PCs **1b** and **1d** were compared to that of PC **3**. As expected, PCs **1b** and **1d** exhibited solvatochromism, which was observed through red‐shifts in emission of 87 nm and 90 nm when comparing solutions in 1‐hexene and DMF, respectively (Figure 6). PC **3**, however, had a relatively solvent‐independent *λ*_max,em_, indicating primarily LE or LE‐dominated HLCT character in the lowest energy singlet excited state. Computations were then employed to futher elucidate whether PC **3** was of LE or LE‐dominated HLCT character in the emissive state.

The computed LUMOs of PCs **1b** and **1d** are exclusively π* orbitals of the *N*‐aryl substituent and their LUMO energies remain unchanged. However, the computed π* orbitals of PC **3** are delocalized across the molecule and are higher in energy relative to PC **1** by 0.65 eV (Figure 5). These data suggest that during internal conversion to the S_1_ state, the LUMOs of PCs **1b** and **1d** will be populated first to form a CT S_1_ state and PC **3** LUMOs will be populated slower and form an LE‐dominated HLCT S_1_ state.

Once PC **3** was observed to exhibit a LE‐dominated HLCT character, we sought to determine the impact CT or LE‐dominated HLCT character has on *E*_S1_, and ultimately, *E*_S1_°*. PC **1d** exhibits a more stabilized emissive state (*λ*_max,em_ = 572 nm; *E*_S1_ = 2.17 eV) in comparison to PC **3** (*λ*_max,em_ = 417 nm; *E*_S1_ = 2.97 eV). Given that PC **3** yields a more destabilized emissive state and the same *E*_1/2_ compared to PC **1d**, we hypothesized that PC **3** will also exhibit a more reducing *E*_S1_°* compared to PC **1d** (Table 1). Excitingly, PC **3** is a highly reducing PC (*E*_S1_°* = −2.48 V vs. SCE) compared to the CT analog PC **1d** (*E*_S1_°* = −1.68 V vs. SCE). These data support the hypothesis that donating electron density to the core of a CT character PC stabilizes the emissive state, thereby leading to a lower *E*_S1_ and a less reducing PC compared to HetCS LE‐dominated HLCT character PCs.

The stark difference in reduction potentials between PC **1d** and PC **3** highlights the influence of CT or LE‐dominated HLCT character on redox potentials. Originally, we hypothesized that increasing electron density of an already electron‐rich core would destabilize *E*_S1_, while stabilizing PC^·+^, resulting in a more reducing PC than PC **1**. While *E*_1/2_ trends support this original hypothesis, we also found that increasing electron density to the core of a CT character PC can also stabilize *E*_S1_, which we posit is due to stabilization of the radical cation‐like character of the core after photoexcitation. Computationally calculated triplet energies follow similar trends as the experimentally determined *E*_S1_. However, heteroatom core substitution could have an effect on triplet excited state lifetimes, *Φ*_ISC_, and nonradiative decay, which will be explored in future work. Overall, the synthesis and subsequent characterization of HetCS PCs has offered clarity into the influence of photophysical properties on redox properties that can be applied to future phenoxazine PC design.

### 2.3 Polymerization Results

After investigating the structure‐property relationships of HetCS phenoxazines, the PCs were employed in O‐ATRP to relate how the properties influence catalytic performance. PCs **1a**, **1b**, **1c**, and **1d** were employed in polymerizations of methyl methacrylate (MMA) using diethyl 2‐bromo‐2‐methyl‐malonate (DBMM) as the initiator and white light LEDs as the light source. Effective O‐ATRP PCs mediate efficient activation and deactivation by possessing a more reducing singlet or triplet excited state than the alkyl bromine initiator or polymer chain‐end and a more oxidizing *E*_1/2_ than the propagating polymer chain. Through previous property‐performance studies, phenoxazine catalyzed O‐ATRP has been able to yield polymers with predictable MW (I* = 91%) and good (*Ð* = 1.19) dispersity.^[^ ^8^ ^]^ These parameters served as our metric of success while analyzing the performance of phenoxazine HetCS PCs.

Considering PC redox properties first, we compared how the identity of the heteroatom substituent impacted performance. Since PCs **1a**, **1b**, **1c**, and **1d** show minimal variation in *E*_S1_°*, we focused on *E*_1/2_. Previous literature suggests that a highly oxidizing PC yields polymers with lower *Ð* due to more efficient deactivation of the propagating polymer chain.^[^ ^18^ ^]^ Considering that PCs **1a** and **1d** show the greatest difference in *E*_1/2_ (*E*_1/2 _= 0.68 V vs. SCE for PC **1a** and *E*_1/2 _= 0.50 V vs. SCE for PC **1d**), we hypothesized that PC **1a** would produce polymeric material with a lower *Ð* compared to PC **1d**. Interestingly, PCs **1a** and **1d** yielded polymers with similar *Ð* (*Ð* = 1.26 and *Ð* = 1.22, respectively) in DMAc, despite their differences in *E*_1/2_ (Table 2). Moreover, *Ð* values remained fairly consistent for polymers catalyzed by all HetCS PCs in their respective solvents. These findings demonstrate that all measured HetCS PCs exhibit a sufficiently oxidizing *E*_1/2_ to efficiently deactivate the propagating polymer chain and achieve polymerization control.

**Table 2:** Results of O‐ATRP catalyzed by HetCS PCs.^[^ ^[a]^, ^[b]^ ^]^

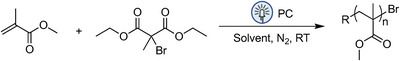					
PC	Solvent^[^ ^[c]^, ^[d]^ ^]^	Conv. [%]^[^ ^e^ ^]^	*M*_n,exp_ [kDa]^[^ ^f^ ^]^	*Ð* ^[^ ^f^ ^]^	I* [%]^[^ ^g^ ^]^
**1a**	DMAc	97.5	14.8	1.26	68
	EtOAc	79.4	7.80	1.37	105
**1b**	DMAc	94.7	13.0	1.20	75
	EtOAc	83.6	8.27	1.34	104
**1c**	DMAc	97.7	18.2	1.22	55
	EtOAc	92.6	10.3	1.39	93
**1d**	DMAc	97.8	21.4	1.21	47
	EtOAc	94.0	10.2	1.41	95
3	DMAc	50.0	81.5	1.62	6

^[a]^ Ratio of [MMA]:[DBMM]:[PC] is [1000]:[10]:[1], 1.0 mL of MMA irradiated with white LED beakers under N_2_.

^[b]^ R is the initiator fragment (diethyl 2‐methylmalonate).

^[c]^ 1.0 mL anhydrous DMAc and time points taken at 22 hrs.

^[d]^ 1.0 mL anhydrous ethyl acetate and time points taken at 24 hrs.

^[e]^ Calculated by ^1^H NMR.

^[f]^ Determined by GPC with multiangle light scattering.

^[g]^ I* calculated through [*M*_n,exp_]/[*M*_n,theo_], where the *M*_n,theo_ is the theoretical number average molecular weight calculated by conversion.

Next, we investigated how solvent polarity altered catalytic performance for PCs **1a**, **1b**, **1c**, and **1d**. Previous studies on phenazine PCs report that polymerizations performed in less polar solvents, such as EtOAc, result in slower conversion and lower *Ð* compared to those performed in more polar solvents, like DMAc.^[^ ^34^ ^]^ Improvements in control are attributed to a stronger PC^·+^Br^−^ ion pair formed in less polar solvents, as well as a more destabilized CT state, leading to a greater driving force for deactivation and improved activation, respectively.^[^ ^22^ ^]^ Consequently, we posit that PCs **1a**, **1b**, **1c**, and **1d** would exhibit greater polymerization control in EtOAc compared to DMAc. These HetCS PCs, however, do not fully follow previously observed trends. While PCs **1a**, **1b**, **1c**, and **1d** do show mildly slower conversions, *Ð* is generally higher in ethyl acetate (*Ð* = 1.37–1.41) than in DMAc (*Ð* = 1.20–1.26) (Table 2). Notably, PCs **1a**, **1b**, **1c**, and **1d** all exhibit lower I* values (I* = 47–75%) in DMAc compared to EtOAc (I* *=* 93–105%*)*, indicating a decreased ability to achieve targeted molecular weight in more polar solvents. These observations suggest additional factors, such as access to the triplet excited state, triplet lifetimes, or additional side reactivity, may be influencing polymerization control in the different solvents and merits further investigation in future studies.

To investigate whether CT character or a more reducing *E*_S1_°* yields better control over polymerizations, we compared the polymerization performance between PC **1d** and PC **3**. PC **1d** is a less reducing PC (*E*_S1_°* = −1.68 V vs. SCE) and exhibits CT character, while PC **3** is more reducing (*E*_S1_°* = −2.46 V vs. SCE) and exhibits LE‐dominated HLCT character in the emissive state. Since PCs **1d** and **3** exhibit similar *E*_1/2_ values, we hypothesized that oxidation potential would not be a factor in differing polymerization control (Table 1). Previously, CT character PCs have exhibited improved polymerization control in comparison to LE character PCs, which is hypothesized to be due to charge separation within the molecule yielding faster electron transfer as well as impacting triplet yields and associated photophysical properties.^[^ ^11^, ^28^ ^]^ Since LE‐dominated HLCT character PCs exhibit delocalized electron density across the entire molecule, we posited that PC **3** would behave similarly to LE character PCs and exhibit worse polymerization control compared to PC **1d**. Indeed, PC **1d** exhibited moderate polymerization control (*Ð* = 1.21; I* = 47%), while PC **3** exhibited poor control (*Ð* = 1.62; I* = 6%) in polymerizations performed in DMAc. Due to PC **3** primarily absorbing in the UV region, UV‐irradiated polymerization control experiments ruled out the possibility that its limited visible light absorption was responsible for poor polymerization control. These data are consistent with previous trends of CT character PCs offering superior polymerization control compared to non‐CT character PCs.

## 3 Conclusion

Investigation of HetCS phenoxazine derivatives has revealed additional factors, such as stabilization through inductive effects and electron destabilization of the core, may also influence *λ*_max,abs_ in addition to extended conjugation. Moreover, characterization of HetCS PCs has highlighted the influence of CT in comparison to LE‐dominated HLCT emissive states on redox properties. Originally, we hypothesized that HetCS would stabilize PC^·+^, while destabilizing *E*_S1_, resulting in a less oxidizing *E*_1/2_ and a more reducing *E*_S1_°*. While the predicted *E*_1/2_ outcome was observed, PCs **1a**, **1b**, **1c**, **1d**, and **2** exhibited relatively unaffected *E*_S1_°* values compared to PC **1**, prompting us to investigate how the photophysical properties are impacting redox characteristics. Our exploration of the emissive states revealed that donating electron density to the core of a PC that exhibits CT to the *N*‐aryl substituent stabilizes the emissive state, whereas donation to a LE‐dominated HLCT character PC core destabilizes the emissive state, resulting in a more reducing *E*_S1_°*. Supporting the hypothesis of electron donation to a LE‐dominated HLCT core yields more reducing PCs, PC **3** exhibited a substantially more reducing *E*_S1_°* (*E*_S1_°* = −2.48 V vs. SCE), while maintaining the same *E*_1/2_ relative to the CT analog PC **1d** (*E*_S1_°* = −1.68 V vs. SCE; *E*_1/2_ = 0.50 V vs. SCE). While the application of PCs **1a**, **1b**, **1c**, and **1d** in O‐ATRP yielded good control in DMAc (*Ð* = 1.20–1.26; I* = 47–75%), the data indicate that further investigation of PC properties is needed to fully understand the factors influencing catalytic performance. Additionally, the poor performance of PC **3** (*Ð* = 1.62; I* = 6%) in DMAc suggests that synthetically tuning a phenoxazine PC to achieve a more reducing *E*_S1_°* will only enhance performance in O‐ATRP if the PC also exhibits CT character in the emissive state. Through the synthesis and characterization of HetCS PCs, new perspectives on previously established structure‐property relationships were uncovered that can help guide the future design of phenoxazine PCs and their applications within photoredox catalysis.

## Supporting Information

The authors have cited additional references within the Supporting Information.^[^ ^8^, ^11^ ^]^

## Conflict of Interest

The authors declare no conflict of interest.

## Supporting information



Supporting Information

## Data Availability

The data that support the findings of this study are available in the supplementary material of this article.
