# Supplementary material for: Impact of Oxygen and Sulfur Heteroatom Core Substitution on Catalyst Properties of Phenoxazines and Their Performance in Organocatalyzed Atom Transfer Radical Polymerization (O‐ATRP)
Source: Chemistry. 2025 Jul 9;31(50):e202501179. doi: 10.1002/chem.202501179 (PMC12415318; doi:10.1002/chem.202501179)
Supplement: Supplementary file 1 — Supporting Information [file CHEM-31-e202501179-s001.pdf]

Supporting Information
for

Impact of Oxygen and Sulfur Heteroatom Core Substitution on Catalyst Properties of Phenoxazines and Their Performance in Organocatalyzed Atom Transfer Radical Polymerization (O-ATRP)

Jessica L. Lathrop, Brandon S. Portela, Robert S. Paton*, Garret M. Miyake*

Department of Chemistry, Colorado State University, 200 W. Lake St., Fort Collins, Colorado 80523, United States

*E-mail: robert.paton@colostate.edu, garret.miyake@colostate.edu

TABLE OF CONTENTS

Materials and Methods 3

Chemical Prep and Storage 3

Experimental Equipment 3

Procedures 4

Synthesis of Photocatalysts 4

Photophysical Characterization 12

UV/Vis Absorption Spectroscopy 12

Emission Spectroscopy 17

Fluorescence Spectroscopy 17

Solvatochromism 19

Cyclic Voltammetry 21

Redox Calculations 24

Supplemental Polymerization Data 24

*Control Polymerization with PC **1a*** 24

General Polymerization Procedure with White LED beakers 24

General Polymerization Procedure with 54 W UV Light 25

O-ATRP of HetCS PCs with 54 W UV Light 25

O-ATRP irradiated with visible and 54 W UV light to compare HLCT and CT PCs 26

Kinetics of MMA polymerization in ethyl acetate 27

Kinetics of MMA polymerization in DMAc 31

Computational Details 34

METHODS AND MATERIALS

Chemical Preparation and Storage

For 1-napthalene-10-phenoxazine synthesis, Phenoxazine was purchased from Accela and sodium *t*-butoxide, anhydrous toluene (Sure/Seal), tri-*t*-butyl phosphine,1-bromonapthalene, and bis(dibenzylideneacetone) palladium(0) were purchased from Sigma-Aldrich.

For 3,7-dibromo-1-napthalene-10-phenoxazine and 3,7-dibromo-1-phenyl-10-phenoxazine synthesis, *N*-bromosuccimide (NBS) was purchased from Sigma-Aldrich and stored at -4 °C in the dark. Chloroform was purchased from Alpha Aesar and glacial acetic acid was purchased from Sigma-Aldrich.

For 1-phenyl-10-phenoxazine synthesis, Ruphos, Ruphos Precat., and anhydrous 1,4-dioxane (Sure/Seal) were purchased from Sigma-Aldrich.

For PC 1a and 1b, tris(dibenzylideneacetone) dipalladium(0), N,N-diisopropylethylamine (DIPEA), and xantphos were purchased from Sigma-Aldrich. Benzenethiol and 4-methoxybenzenthiol were both purchased from Sigma-Aldrich, freeze pump thawed three times, and stored under N_2 upon arrival.

For PC 1c, 1d, and 3, copper (I) iodide and N,N-dimethylglycine hydrochloride were purchased from Sigma-Aldrich. Phenol and 4-methoxyphenol were purchased from Alpha Aesar. Cesium carbonate was purchased from Ambeed.

For PC 2, copper (I) iodide and anhydrous dimethyl sulfoxide (Sure/Seal) were purchased from Sigma-Aldrich. 1,4-diazabicyclo [2.2.2.] octane (DABCO) was purchased from Oakwood Chemical.

For all polymerizations, methyl methacrylate (MMA) and diethyl 2-bromo-2-methyl-malonate (DBMM) were purchased from Sigma-Aldrich and stirred over calcium hydride overnight. Both were distilled under reduced pressure and freeze pump thawed three times before storing them under N_2 at -40 °C in the dark. MMA and DBMM were warmed up to room temperature prior to using. Anhydrous ethyl acetate and dimethylacetamide (Sure/Seal) were purchased from Sigma-Aldrich and stored under N_2.

For PC characterization, DMAc was purchased from Sigma-Aldrich in a Sure/Seal and UPLC grade acetonitrile was purchased from Sigma-Aldrich. Solvents for solvatochromism (1-hexene, benzene, 1,4-dioxane, THF, pyridine, and DMF) were purchased from Sigma-Aldrich, Sigma-Aldrich, Oakwood Chemical, Sigma-Aldrich, Sigma-Aldrich, and Aqua Solutions Inc., respectively.

Experimental Equipment

Light beakers used for polymerizations were lined with 51 LED lights from double-density white LED Strips purchased from Creative Lighting Solutions (item no. CL-FRS1210-5M-12V-WH). Light beakers were constructed through wrapping 51 LED lights on the inside of a 500 mL beaker and wrapping the outside of the beaker in aluminum foil. The light intensity at the center of the beaker was determined to be 437 Lux, as measured by an Amprobe LED light meter.

54 W UV light used for polymerizations was a Vogue Professional Powerful & Double Wide 54 watt UV lamp Light Nail Dryer wrapped in aluminum foil. The UV light emits 365 nm and has a fan built in.

Electrochemistry: For all cyclic voltammetry measurements, a 3-electrode electrochemical cell was utilized. The three electrodes used were a glassy carbon working electrode, platinum counter electrode, and the reference electrode contained a silver/silver

nitrate solution (0.1 M $Ag/AgNO_3$ and 0.1 M Bu_4NPF_6 in acetonitrile). PC **1**, **1a**, **1b**, **1c**, **1d**, and **3** were prepared at 2mM concentration in DMAc and 0.1 M Bu_4NPF_6 was added. PC **2** was analyzed at an unknown concentration in DMAc with 0.1 M Bu_4NPF_6. All samples were sparged with N_2 for 15 minutes prior to running the experiment and the reference electrode solution was prepared the same day as the experiments. The glassy carbon electrode was polished by making figure 8's in an alumina slurry on polishing paper prior to running CV each day.

Nuclear magnetic resonance (NMR) Spectroscopy. Bruker US400, Bruker Ascend 400, and Bruker NEO400 were all utilized for ^{1}H NMR and ^{13}C NMR characterization. All ^{1}H NMR characterization is reported in parts per million (ppm) units and was measured relative to solvent residual peaks of deuterated benzene (7.16 ppm) and deuterated DMSO (2.50 ppm). All ^{13}C NMR characterization is reported in ppm relative to residual solvent peaks of deuterated benzene (128.06 ppm) and deuterated DMSO (39.52 ppm).

Matrix-assisted laser desorption/ionization (MALDI) Mass Spectrometry. High resolution mass spectrometry was performed using a Bruker UltrafleXtreme MALDI-TOF/TOF.

UV/Visible Spectroscopy. UV/Visible spectroscopy was performed using Agilent Cary 5000 UV-Vis-NIR-Spectrometer.

Fluorescence Spectroscopy. Steady state fluorescence spectroscopy was performed on FS5 Spectrofluorometer from Edinburgh Instruments.

Gel Permeation Chromatography (GPC). Polymer molecular weight analyses were performed using gel permeation chromatography (GPC) and multi-angle light scattering (MALS). The HPLC stack was purchased from Agilent and equipped with one guard column and three PLgel 5 µm MIXED-C gel permeation columns. A TrEX differential refractometer and miniDAWN TREOS light scattering detector from Wyatt Technologies were also used in polymer analysis. Polymerization analysis experiments were run in HPLC grade THF at a flow rate of 1.0 mL/min. The dn/dc value of 0.084 was used for PMMA molecular weight analysis.

SYNTHETIC PROCEDURES

Synthesis of Photocatalysts

Syntheses of PC **1**, 1-phenyl-10-phenoxazine, 3,7-dibromo-1-napthalene-10-phenoxazine, and 3,7-dibromo-1-phenyl-10-phenoxazine were performed using previous literature procedures.[8,11]

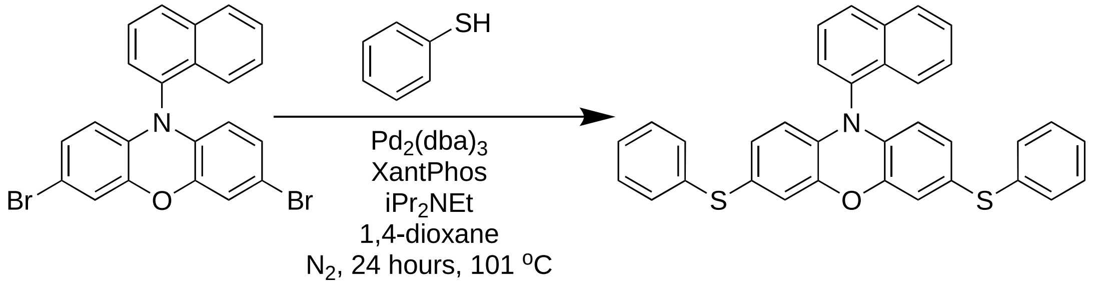

Figure S1. Scheme for the synthesis of **PC 1a.**

Synthesis of 3,7-di(benzenethiol)-1-napthalene-10-phenoxazine (PC 1a). 3,7-dibromo-1-napthalene-10-phenoxazine (374.9 mg, 0.8025 mmol, 1.0 eq) was charged to an oven dried

storage tube and back cycled with nitrogen three times. $Pd_2(dba)_3$ (183.7 mg, 0.2006 mmol, 0.25 eq) and Xantphos (232.2 mg, 0.4013 mmol, 0.50 eq) were added to the storage tube. Benzenethiol (0.327 mL, 3.21 mmol, 6.0 eq) was added to the mixture and 10.0 mL of anhydrous 1,4-dioxane was used to dissolve the reaction mixture. *i*Pr_2NEt (0.559 mL, 3.21 mmol, 6.0 eq) was added outside of the glovebox and the reaction mixture was sparged for 15 minutes. The reaction was heated to 101 °C and let stir for 24 hours under nitrogen. Upon completion of the reaction, the reaction mixture was diluted with DCM, washed with DI water x3, brine x2, and dried over anhydrous $MgSO_4$. The drying agent was filtered off via silica plug to remove residual palladium catalyst. The remaining reaction mixture was concentrated under reduced pressure. The residue was purified through flash chromatography with 1:9 toluene to hexanes with 5% TEA as the eluent. Yield: 202 mg (47.9%). ^{1}H NMR (400 MHz, $(CD_3)_2SO$) δ (ppm): 8.11-8.20 (m, 2H, *J* = 13.3 Hz); 7.98 (d, 1H, *J* = 8.2 Hz); 7.29-7.38 (t, 4H, *J* = 7.5 Hz); 7.21-7.26 (m, 6H, *J* = 7.6 Hz).

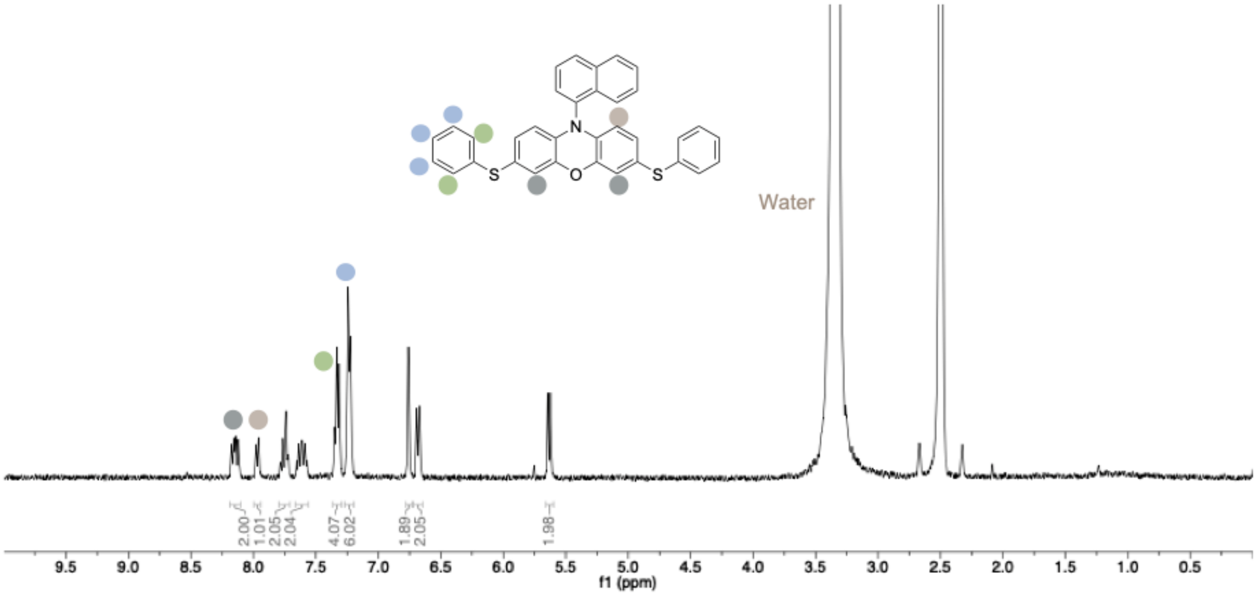

Figure S2. ^{1}H NMR spectrum of **PC 1a** in $(CD_3)_2SO$ at 23 °C with relevant peaks labeled.

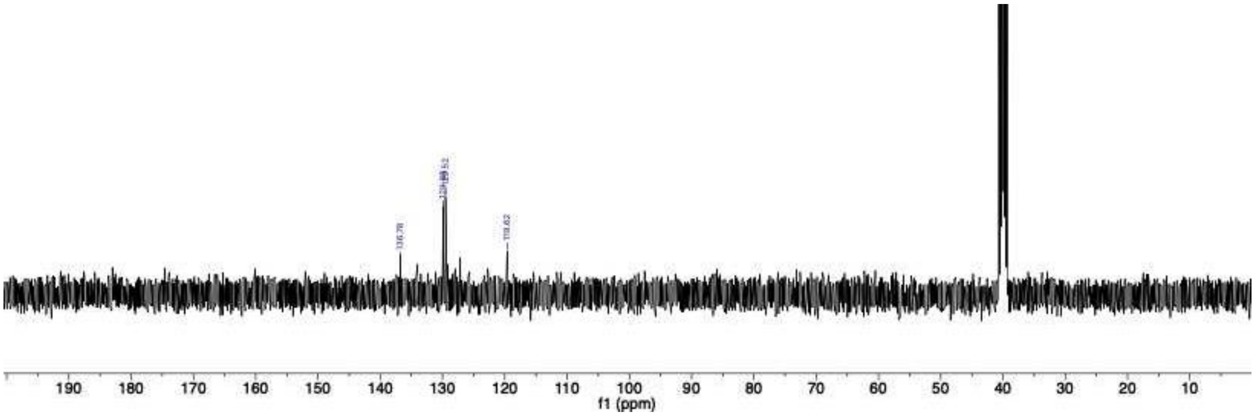

Figure S3. ^{13}C NMR spectrum of **PC 1a** in $(CD_3)_2SO$ at 23 °C with relevant peaks labeled.

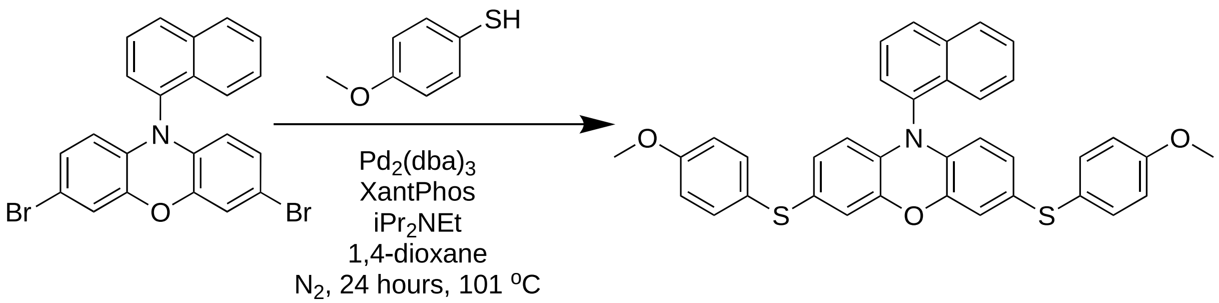

Figure S4. Scheme for the synthesis of **PC 1b.**

Synthesis of 3,7-di(4-methoxybenzenethiol)-1-napthalene-10-phenoxazine (PC 1b). 3,7-dibromo-1-napthalene-10-phenoxazine (249.9 mg, 0.5349 mmol, 1.0 eq) was charged to an oven dried storage tube and back cycled with nitrogen three times. $Pd_2(dba)_3$ (122.5 mg, 0.1337 mmol, 0.25 eq) and Xantphos (154.8 mg, 0.2675 mmol, 0.50 eq) were added to the storage tube. 4-methoxybenzenethiol (0.263 mL, 2.14 mmol, 4.0 eq) was added to the mixture and 15.0 mL of anhydrous 1,4-dioxane was used to dissolve the reaction mixture. *i*Pr_2NEt (0.373 mL, 2.14 mmol, 4.0 eq) was added outside of the glovebox followed by sparging of the reaction mixture for 15 minutes. The reaction was stirred at 101 °C for 24 hours under nitrogen. Upon completion of the reaction, the reaction mixture was diluted with DCM, washed with DI water x3, brine x2, and dried over anhydrous $MgSO_4$. The drying agent was filtered off via silica plug to remove residual palladium catalyst. The remaining reaction mixture was concentrated under reduced pressure. The residue was purified through flash chromatography with 3:5 ethyl acetate to hexanes with 5% TEA as the eluent. Yield: 224 mg (71.5%). ^1H NMR (400 MHz, $(CD_3)_2SO$) δ (ppm): 8.08-8.18 (m, 2H, *J* = 9.2 Hz); 7.31 (d, 4H, *J* = 1.7 Hz); 6.94 (d, 4H); 3.74 (s, 6H).

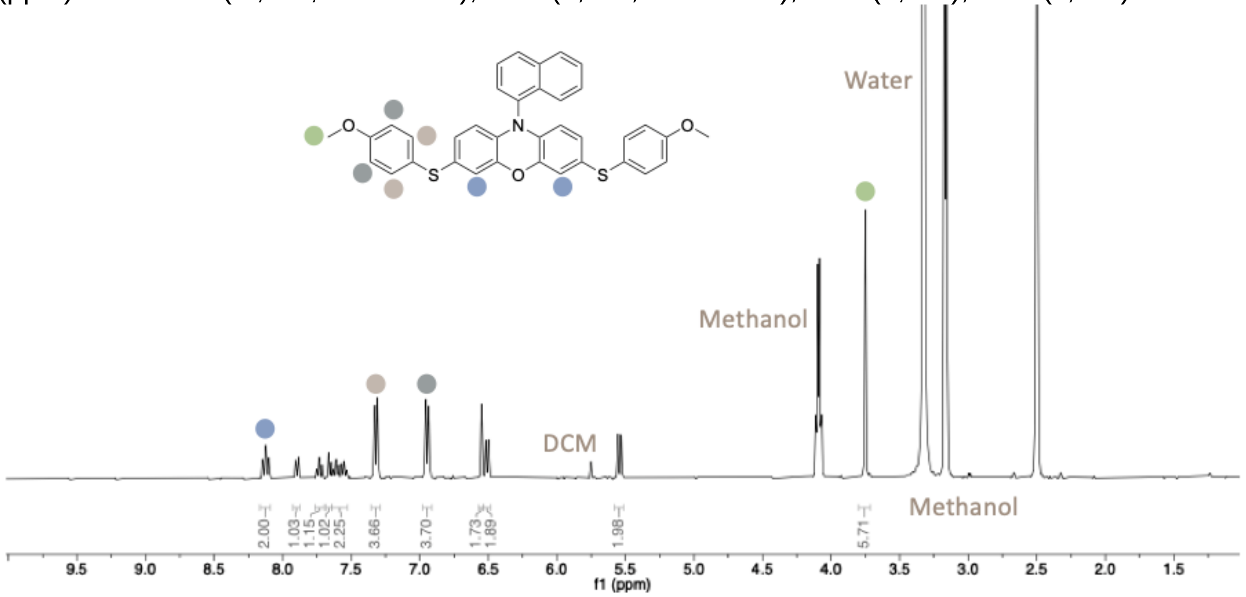

Figure S5. ^1H NMR spectrum of **PC 1b** in $(CD_3)_2SO$ at 23 °C with relevant peaks labeled.

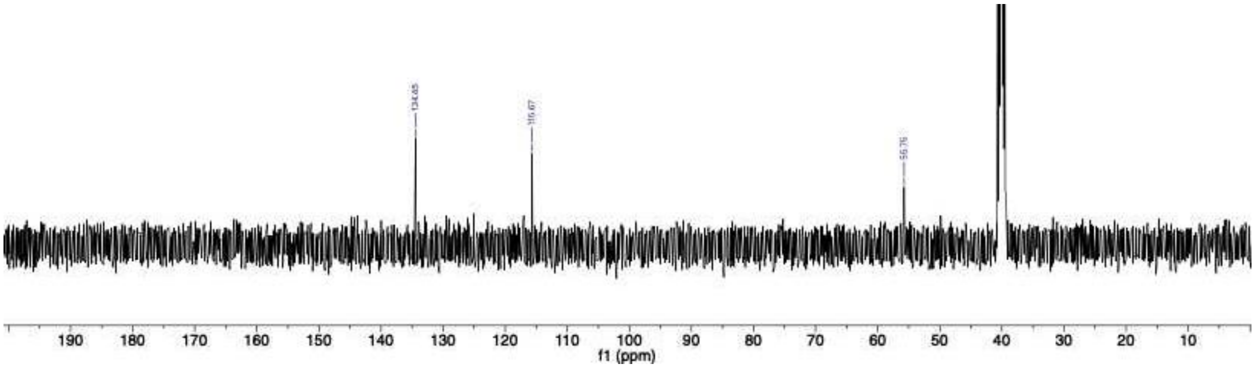

Figure S6. ^{13}C NMR spectrum of **PC 1b** in $(CD_3)_2SO$ at 23 °C with relevant peaks labeled.

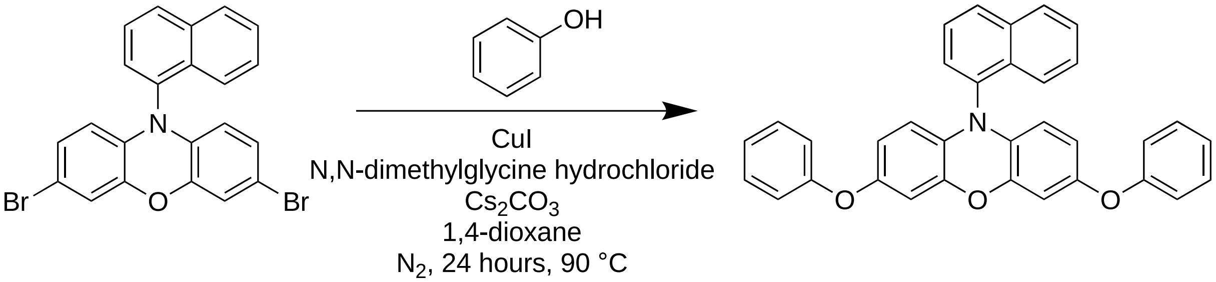

Figure S7. Scheme for the synthesis of **PC 1c.**

Synthesis of 3,7-di(phenol)-1-napthalene-10-phenoxazine (PC 1c). 3,7-dibromo-1-napthalene-10-phenoxazine (199.7 mg, 0.4256 mmol, 1.0 eq.), phenol detached crystals (240.3 mg, 2.554 mmol, 6.0 eq.), N,N-dimethylglycine hydrochloride (41.6 mg, 0.298 mmol, 0.70 eq.) and cesium carbonate (833.0 mg, 2.560 mmol, 6.0 eq.) were charged to an oven dried storage tube. The storage tube was backfilled with nitrogen three times. Copper iodide (24.4 mg, 0.128 mmol, 0.30 eq.) was added to the reaction mixture and dissolved with 4.0 mL anhydrous 1,4-dioxane. The reaction was stirred at 90 °C for 24 hours under nitrogen. Once the reaction was complete, the reaction mixture was diluted with ethyl acetate. The organic layer was washed three times with DI water, brine twice, and dried over $MgSO_4$. The drying agent was filtered off and the reaction mixture was concentrated under reduced pressure. The remaining solid was purified via flash chromatography with 1:9 toluene to hexanes with 5% TEA solvent system. Yield: 60 mg (28.0%). ^1H NMR (400 MHz, $(CD_3)_2SO$) δ (ppm): 8.13 (t, 2H, J = 7.7 Hz); 7.98 (d, 1H, J = 7.8 Hz); 7.34 (t, 4H); 7.07 (t, 2H); 6.95 (d, 4H).

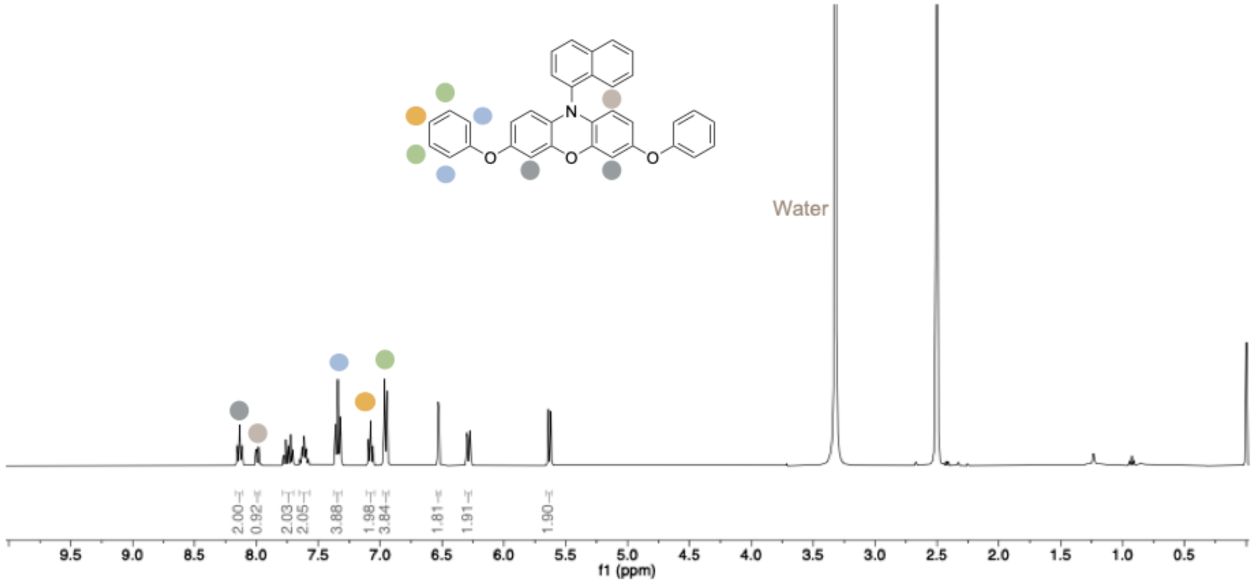

Figure S8. ^{1}H NMR spectrum of **PC 1c** in $(CD_3)_2SO$ at 23 °C with relevant peaks labeled.

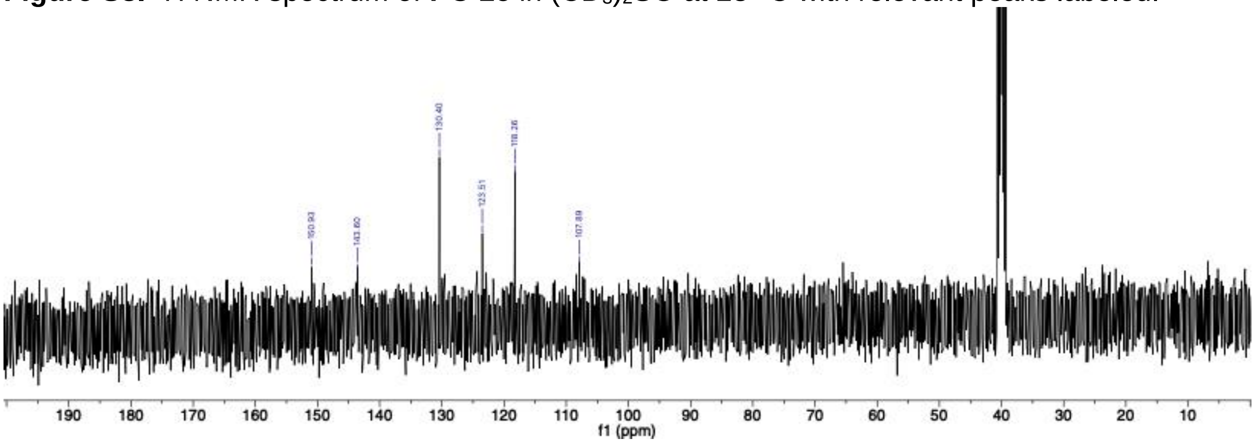

Figure S9. ^{13}C NMR spectrum of **PC 1c** in $(CD_3)_2SO$ at 23 °C with relevant peaks labeled.

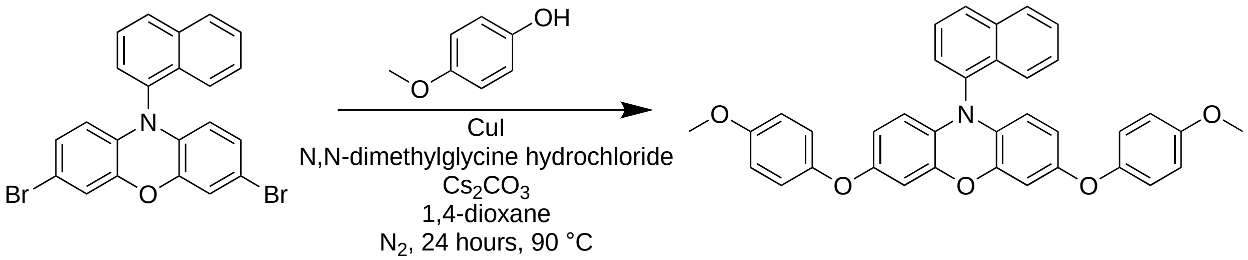

Figure S10. Scheme for the synthesis of **PC 1d.**

Synthesis of 3,7-di(4-methoxyphenol)-1-napthalene-10-phenoxazine (PC 1d). 3,7-dibromo-1-napthalene-10-phenoxazine (151.8 mg, 0.3235 mmol, 1.0 eq.), 4-methoxyphenol (241.0 mg, 1.941 mmol, 6.0 eq.), cesium carbonate (632.5 mg, 1.941 mmol, 6.0 eq.) and N,N-dimethylglycine hydrochloride (31.6 mg, 0.226 mmol, 0.70 eq.) were charged to an oven dried storage tube. The storage tube was cycled with nitrogen three times. Copper iodide (18.5 mg, 97.1 µmol, 0.30 eq.) was added to the reaction mixture. 3.0 mL of dry 1,4-dioxane was used to dissolve the reaction mixture. The reaction was heated to 90 °C for 24 hours. Once the reaction

was complete, the reaction mixture was diluted with ethyl acetate and washed with DI water three times. The organic layer was washed with brine twice and dried over $MgSO_4$. The drying agent was filtered off and concentrated under reduced pressure to yield a solid. The solid was purified via flash chromatography with 1:9 toluene to hexanes with 5% TEA. Yield: 57.0 mg (31.7%). ^1H NMR (400 MHz, $(CD_3)_2SO$) δ (ppm): 6.92 (t, 8H); 3.71 (s, 6H, J = 1.45 Hz).

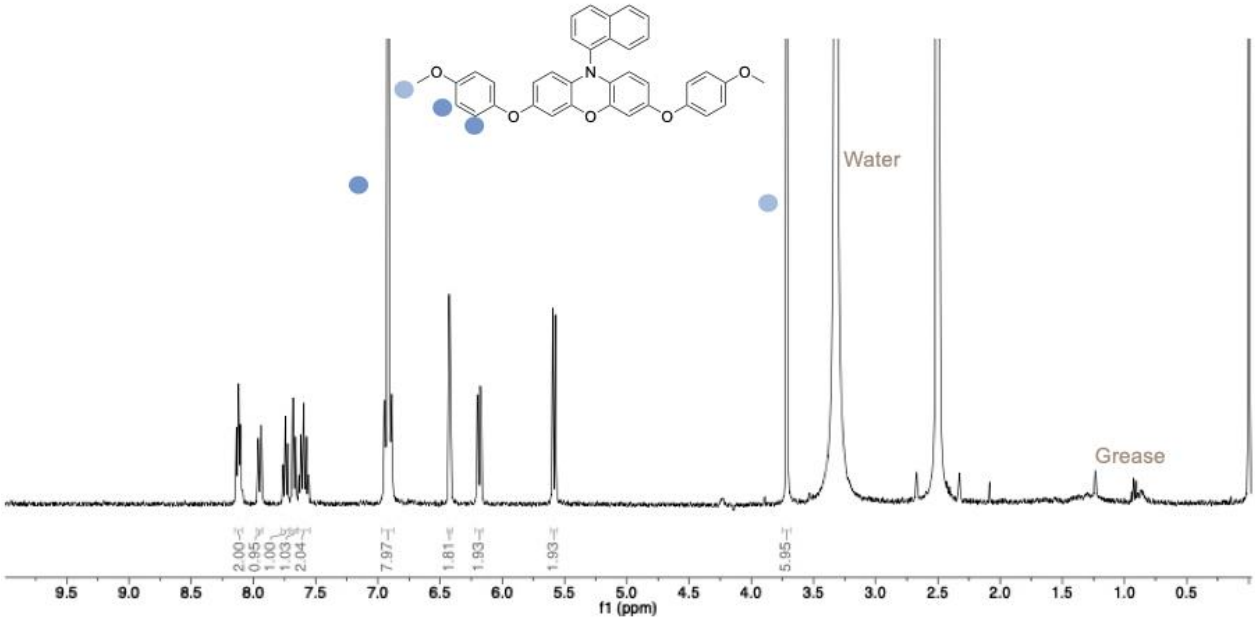

Figure S11. ^1H NMR spectrum of **PC 1d** in $(CD_3)_2SO$ at 23 °C with relevant peaks labeled.

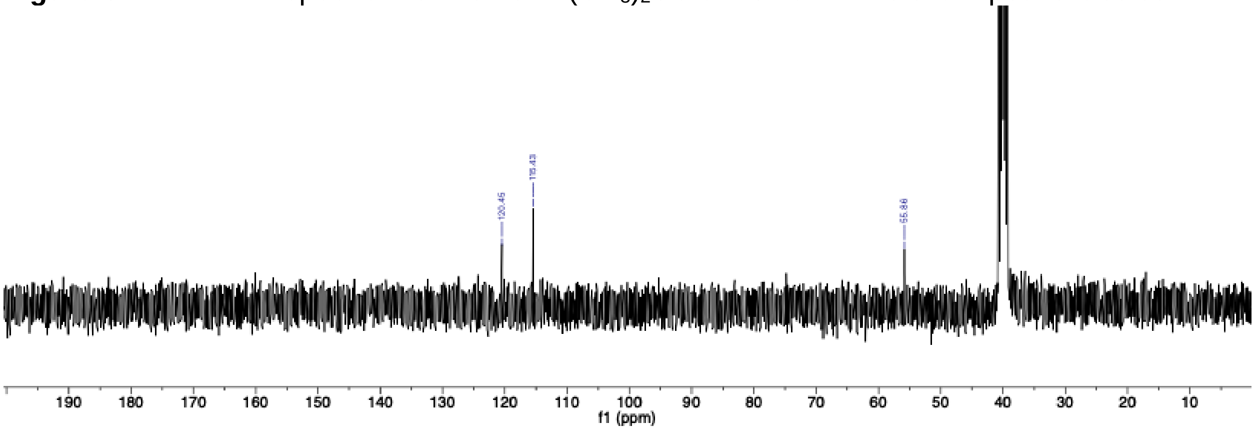

Figure S12. ^{13}C NMR spectrum of **PC 1d** in $(CD_3)_2SO$ with relevant peaks labeled.

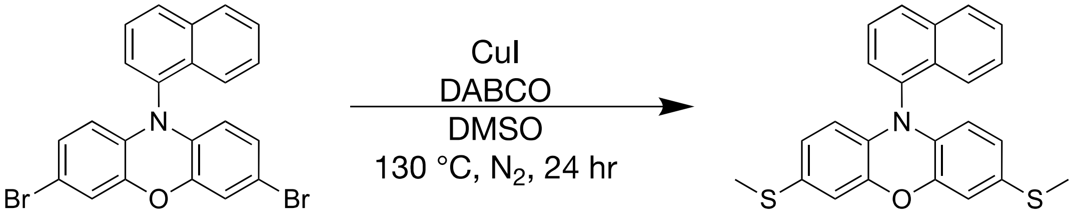

Figure S13. Reaction scheme for synthesis of **PC 2.**

Synthesis of 3,7-dimethanethiol-1-napthalene-10-phenoxazine (PC 2). 3,7-dibromo-1-napthalene-10-phenoxazine (100.0 mg, 0.2141 mmol, 1.0 eq.) and DABCO (144.1 mg, 1.284

mmol, 6.0 eq.) were charged to an oven dried storage tube. The storage tube was cycled with nitrogen three times. Copper iodide (12.2 mg, 64.2 µmol, 0.30 eq.) was added to the reaction mixture. 5.0 mL of dry DMSO was used as the sulfur source and to dissolve the reaction mixture. The reaction was heated to 130 °C for 24 hours. Once the reaction was complete, the reaction mixture was diluted with ethyl acetate and washed with DI water three times. The organic layer was washed with brine twice and dried over $MgSO_4$. The drying agent was filtered off and concentrated under reduced pressure to yield a solid. The resulting solid was purified through flash chromatography via hexanes with 5% TEA. Yield: 2.50 mg (2.91%). ^{1}H NMR in C_6D_6 δ (ppm): 5.58 (d, 2H, *J* = 8.31 Hz); 1.98 (s, 6H).

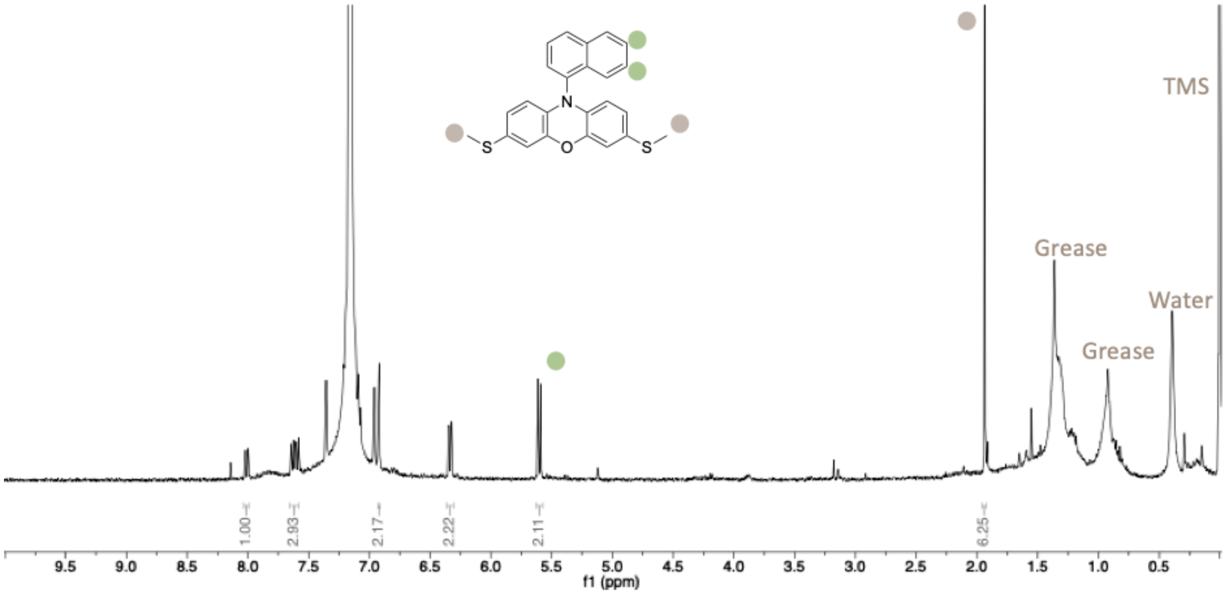

Figure S14. ^{1}H NMR spectrum of **PC 2** in C_6D_6 at 23 °C with relevant peaks labeled.

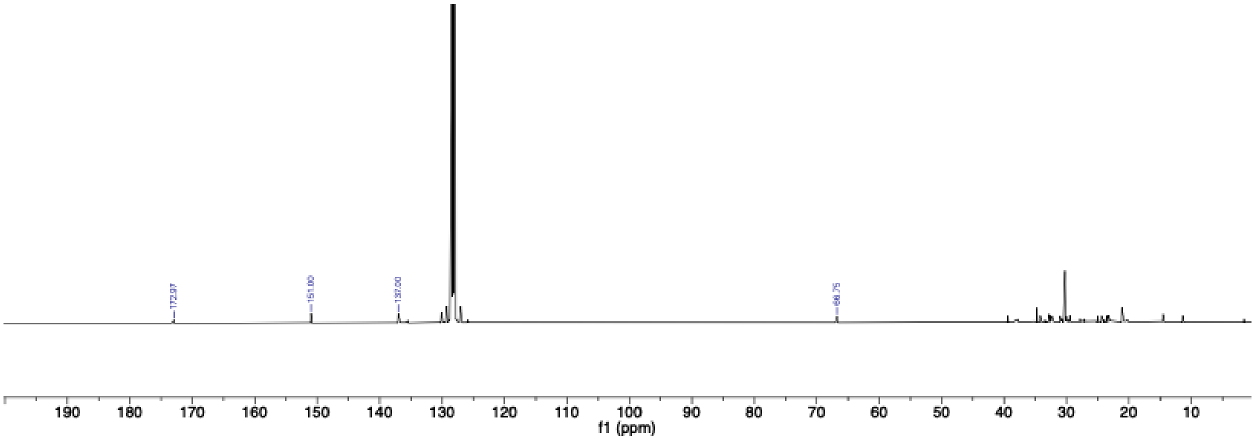

Figure S15. ^{13}C NMR spectrum of **PC 2** in C_6D_6 at 23 °C with relevant peaks labeled.

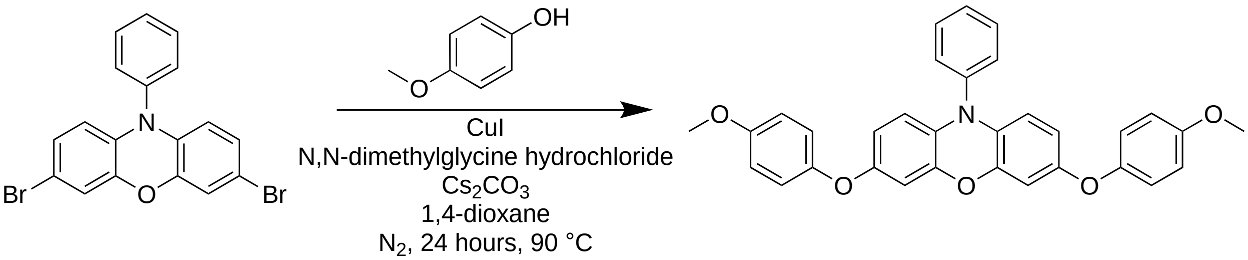

Figure S16. Reaction scheme for synthesis of **PC 3.**

Synthesis of 3,7-di(4-methoxyphenol)-1-phenyl-10-phenoxazine (PC 3). 3,7-dibromo-1-phenyl-10-phenoxazine (250.0 mg, 0.5965 mmol, 1.0 eq.), 4-methoxyphenol (444.3 mg, 3.579 mmol, 6.0 eq.), cesium carbonate (1.1660 g, 3.5790 mmol, 6.0 eq.) and N,N-dimethylglycine hydrochloride (58.3 mg, 0.418 mmol, 0.70 eq.) were charged to an oven dried storage tube. The storage tube was cycled with nitrogen three times. Copper iodide (34.1 mg, 0.179 mmol, 0.30 eq.) was added to the reaction mixture. 4.0 mL of dry 1,4-dioxane was used to dissolve the reaction mixture. The reaction was heated to 90 °C for 24 hours. Once the reaction was complete, the reaction mixture was diluted with ethyl acetate and washed with DI water x3. The organic layer was washed with brine x2 and dried over $MgSO_4$. The drying agent was filtered off and concentrated under reduced pressure to yield a solid. The solid was purified via flash chromatography with 1:1:8 toluene to ethyl acetate to hexanes with 5% TEA in the dark. Yield: 82.0 mg (27.2%). ^{1}H NMR (400 MHz, $(CD_3)_2SO$) δ (ppm): 7.65 (t, 2H, J = 7.7 Hz); 6.96 (m, 8H); 3.71 (s, 6H, J = 1.4 Hz).

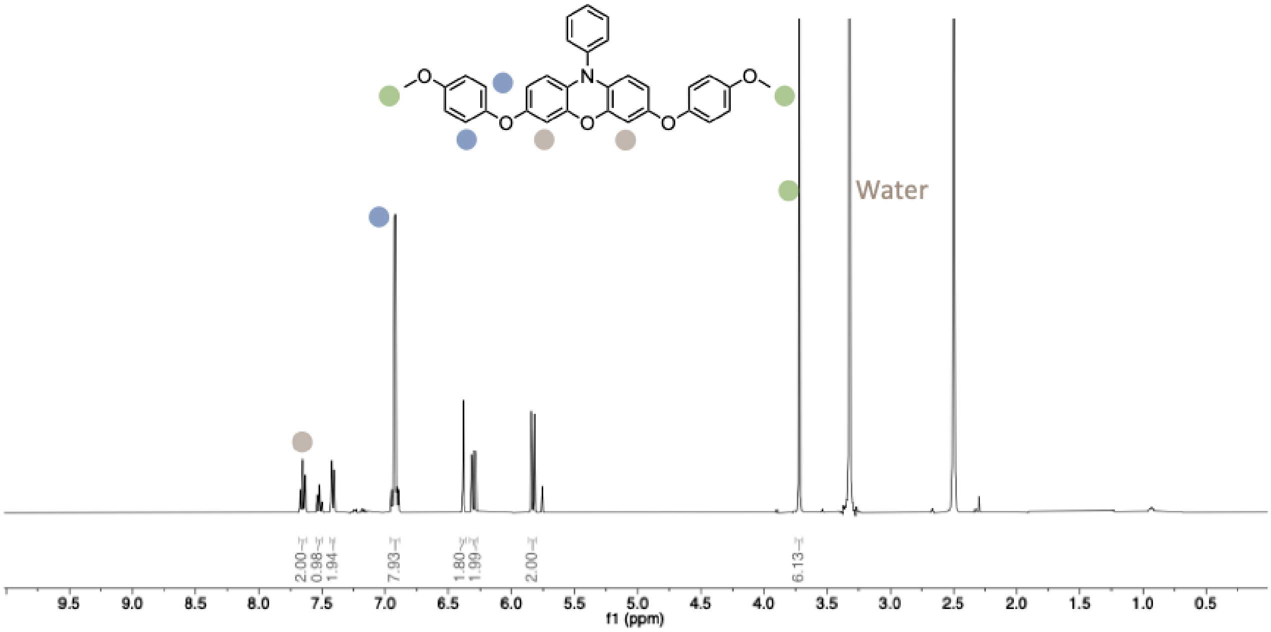

Figure S17. ^{1}H NMR spectrum of **PC 3** in $(CD_3)_2SO$ at 23 °C with relevant peaks labeled.

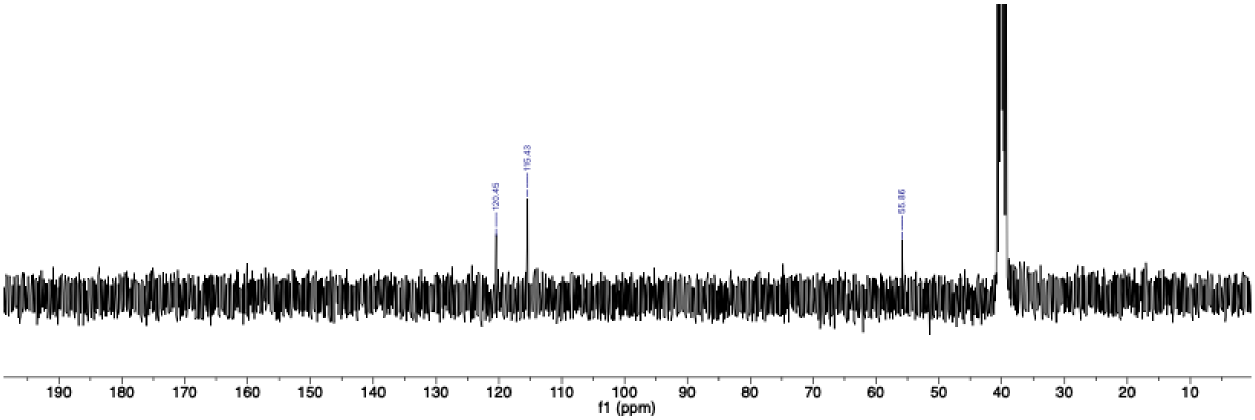

Figure S18. ^{13}C NMR spectrum of **PC 3** in $(CD_3)_2SO$ at 23 °C with relevant peaks labeled.

MALDI-TOF Mass Spectrometry of 1a-1d PCs

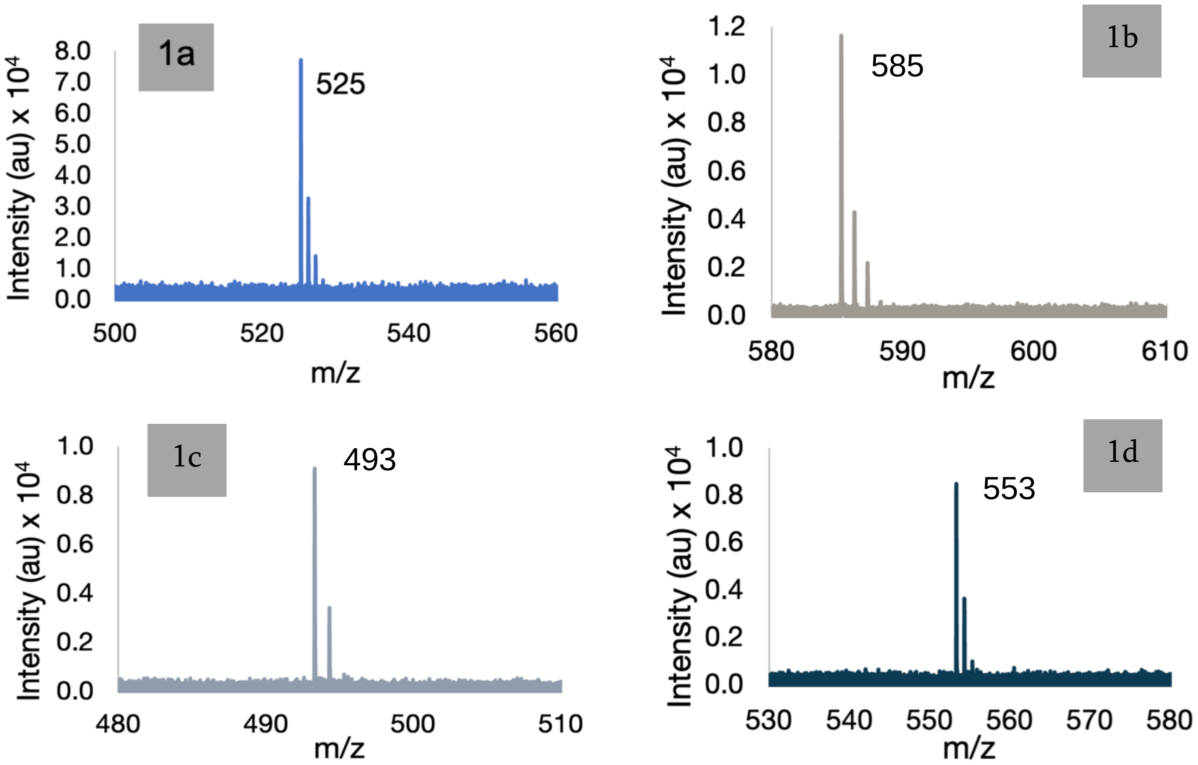

Figure S19. MALDI-TOF mass spectra of PCs **1a**, **1b**, **1c**, and **1d** using positive ion reflector mode.

PHOTOCATALYST CHARACTERIZATION

UV/Vis Absorption Spectroscopy

$\lambda_{max,abs}$ measurements were conducted on a Cary series UV-Vis-NIR spectrophotometer. Molar absorptivity measurements for PCs **1a**, **1b**, **1c**, **1d**, and **3** were made in a stock solution of 2mM

in DMAc and diluted to different concentrations. $\lambda_{max,abs}$ for PCs **1** and **2** were determined at an unknown concentration. Beer-Lambert's Law was not performed on PCs **1** or **2**.

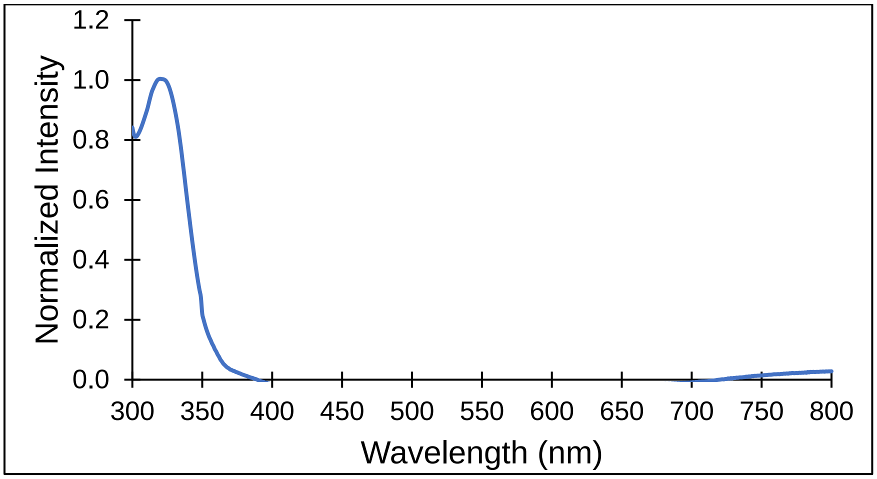

Figure S20. UV/Vis absorption of PC **1** at an unknown concentration in DMAc.

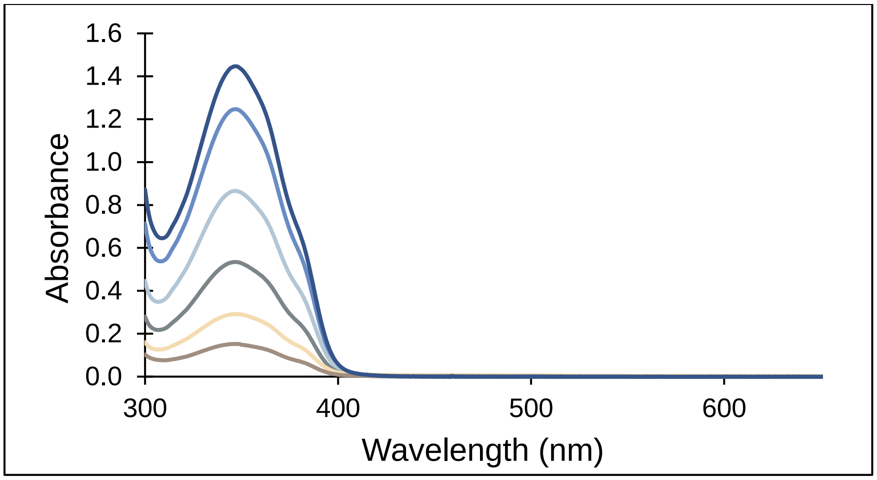

Figure S21. UV/Vis absorption for PC **1a** in 6 different concentrations in DMAc.

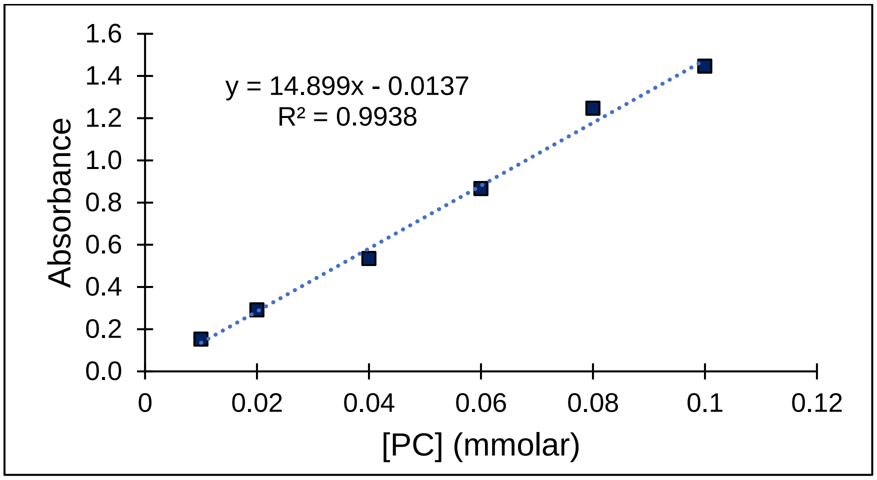

Figure S22. Beer-Lambert's law plot for determination of molar absorptivity of PC **1a** in DMAc at λ_{max} = 347 nm.

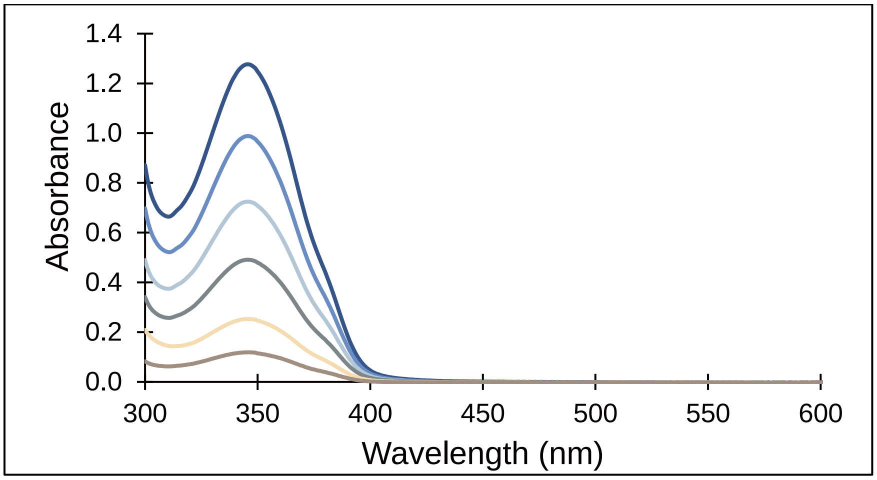

Figure S23. UV/Vis absorption for PC **1b** in 6 different concentrations in DMAc.

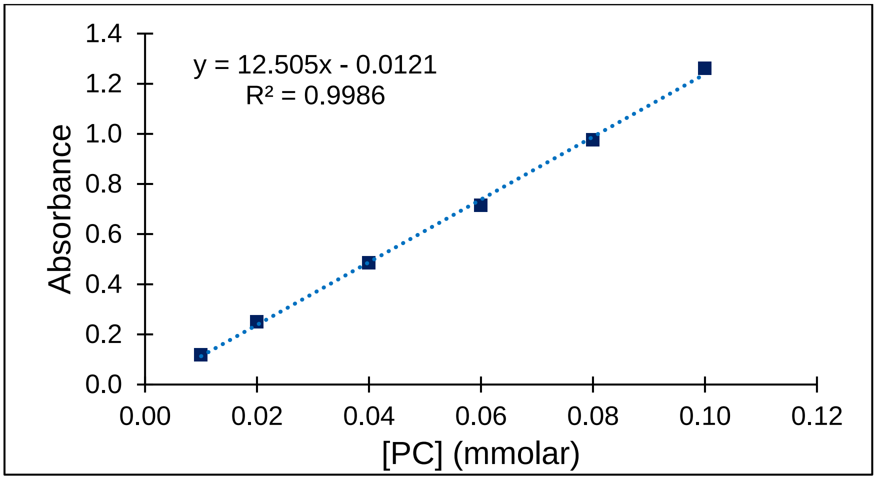

Figure S24. Beer-Lambert's law plot for determination of molar absorptivity of PC **1b** in DMAc at λ_{max} = 346 nm.

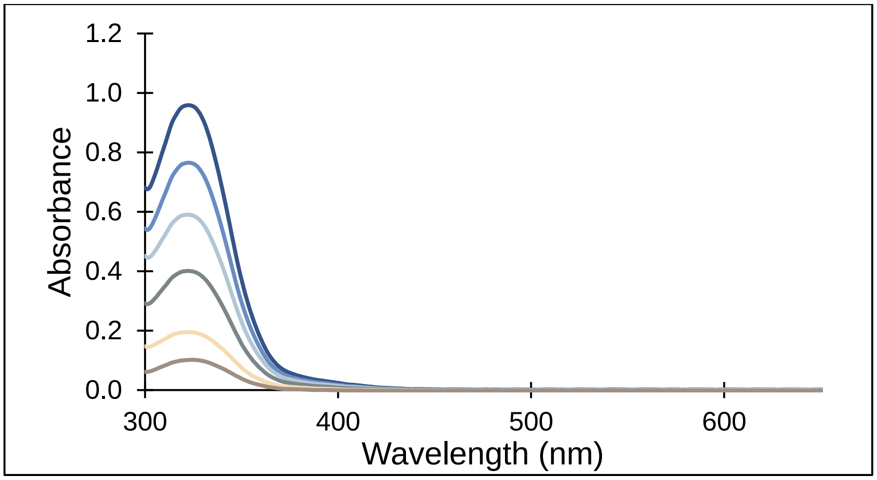

Figure S25. UV/Vis absorption for PC **1c** in 6 different concentrations in DMAc.

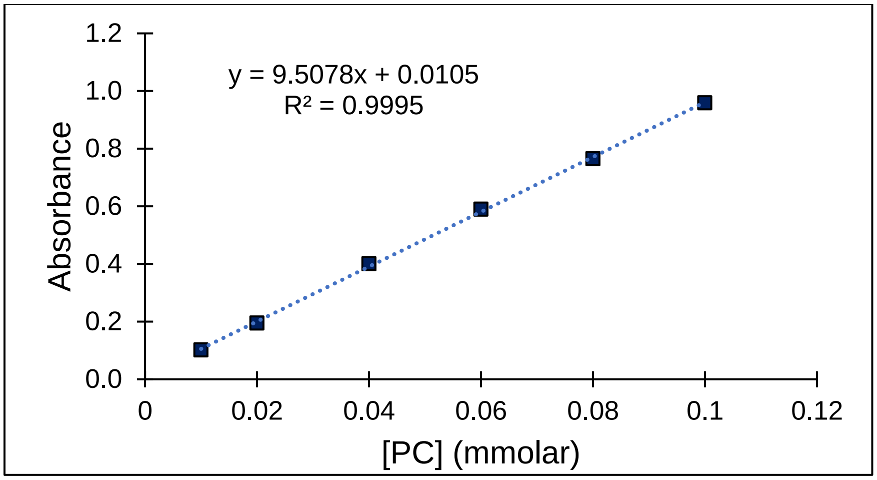

Figure S26. Beer-Lambert's law plot for determination of molar absorptivity of PC **1c** in DMAc at λ_{max} = 323 nm.

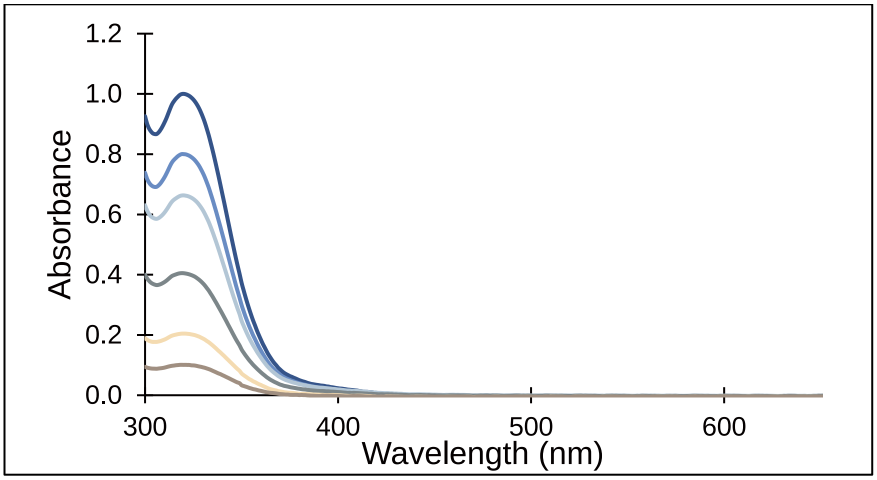

Figure S27. UV/Vis absorption for PC **1d** in 6 different concentrations in DMAc.

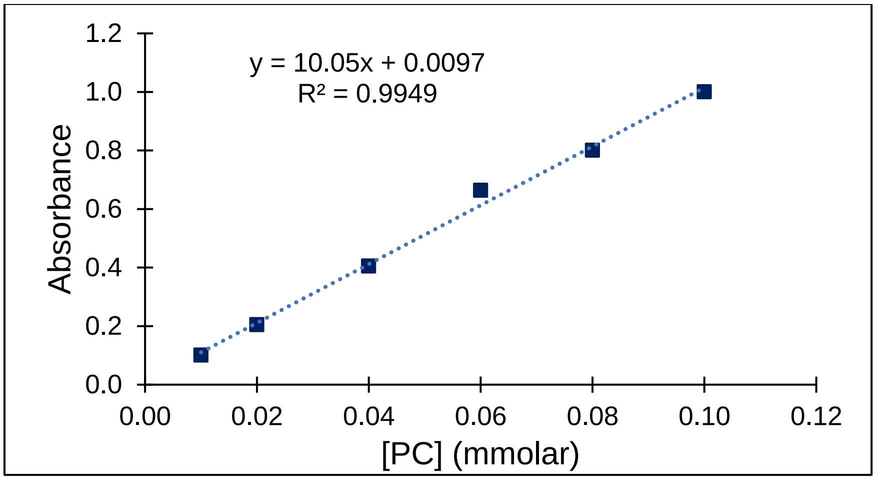

Figure S28. Beer-Lambert's law plot for determination of molar absorptivity of PC **1d** in DMAc at λ_{max} = 320 nm.

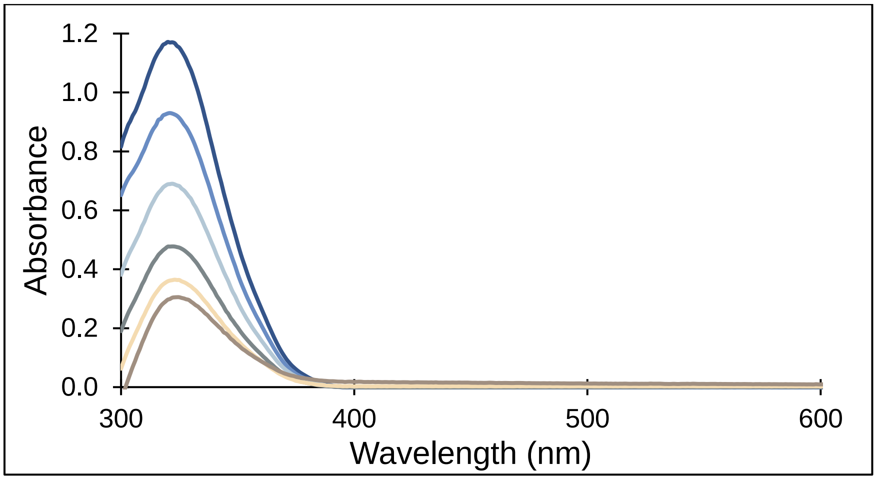

Figure S29. UV/Vis absorption for PC **3** in 6 different concentrations in DMAc.

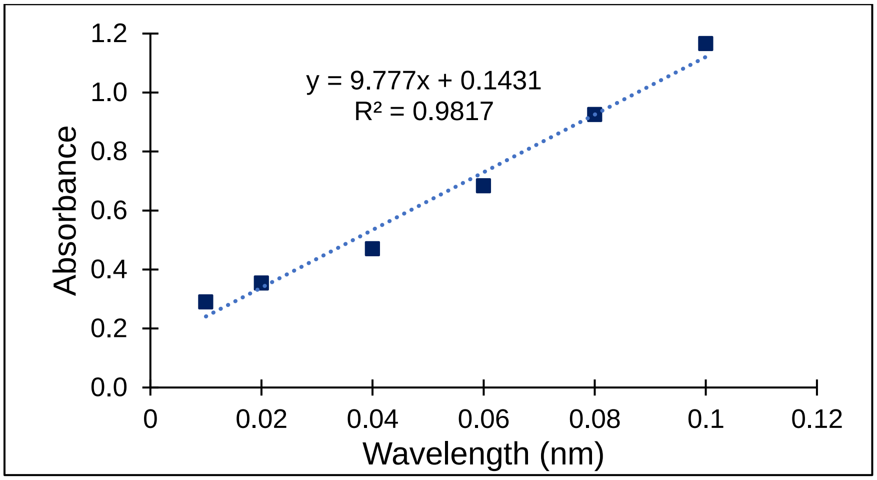

Figure S30. Beer-Lambert's law plot for determination of molar absorptivity of PC **3** in DMAc at λ_{max} = 319 nm.

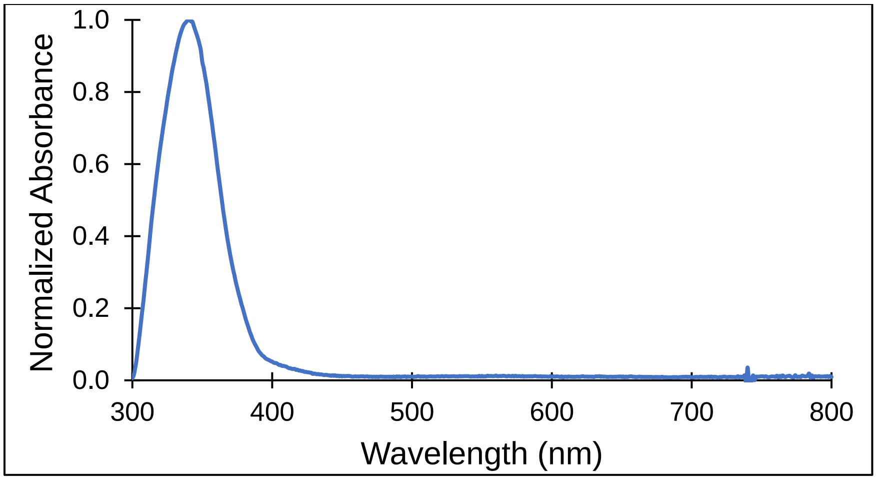

Figure S31. UV/Vis absorption of PC **2** at an unknown concentration in DMAc.

<u>Emission</u>

Fluorescent Spectroscopy
PCs **1a**, **1b**, **1c**, and **1d** were measured on a FS5 spectrofluorometer at 0.10 mM concentration in DMAc while PCs **1**, **2**, and **3** were measured at an unknown concentration. Emission experiments were conducted in triplicate with a step size of 1 nm.

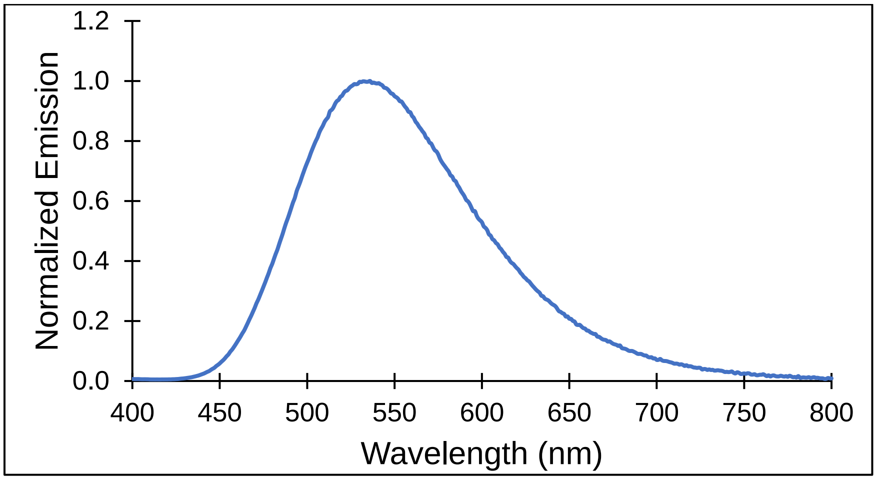

Figure S32. Fluorescence spectrum of PC **1** in DMAc excited at 340 nm.

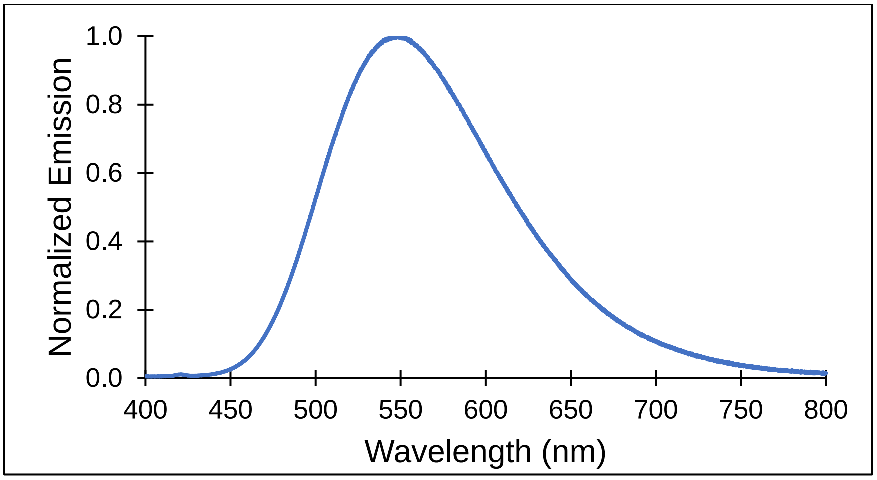

Figure S33. Fluorescence spectrum of PC **1a** in DMAc excited at 367 nm.

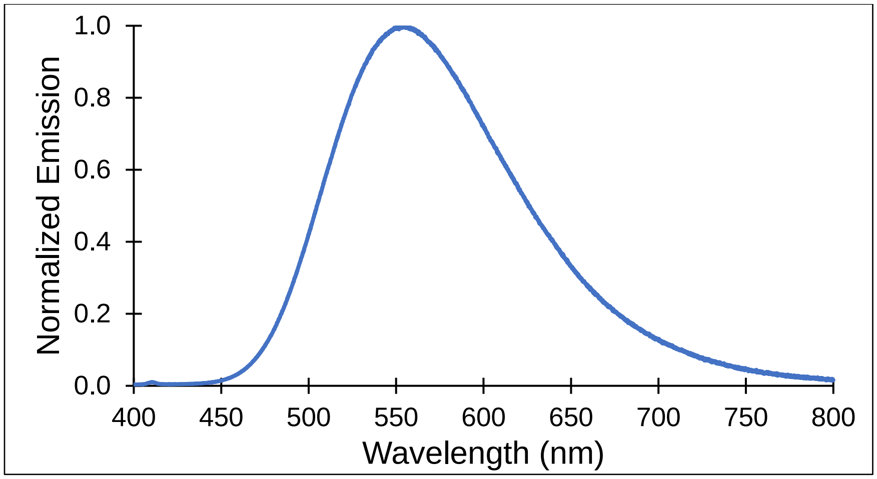

Figure S34. Fluorescence spectrum of PC **1b** in DMAc excited at 366 nm.

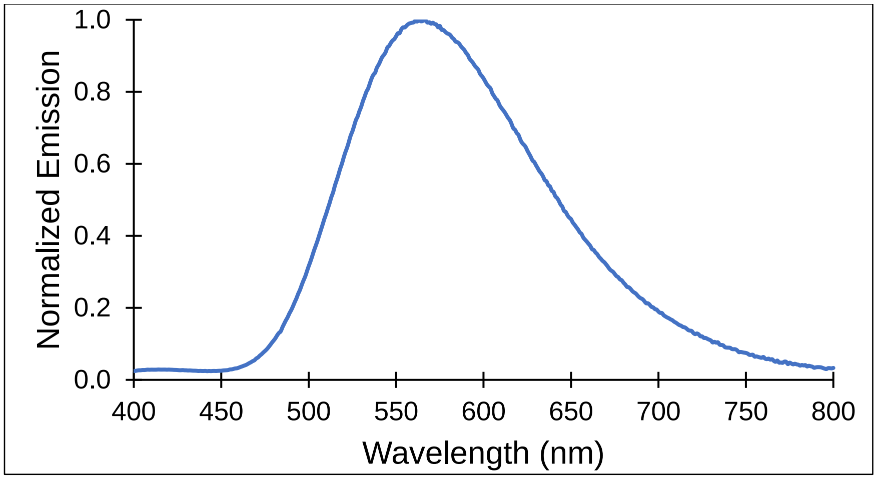

Figure S35. Fluorescence spectrum of PC **1c** in DMAc excited at 343 nm.

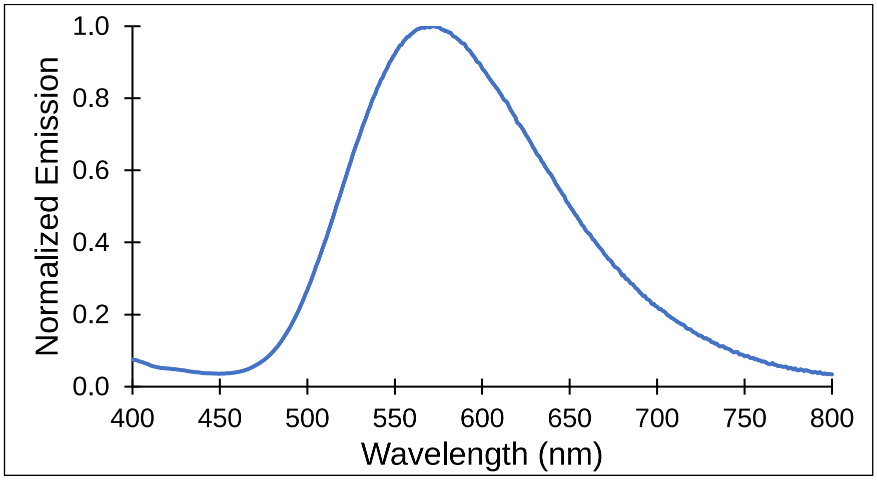

Figure S36. Fluorescence spectrum of PC **1d** in DMAc excited at 340 nm.

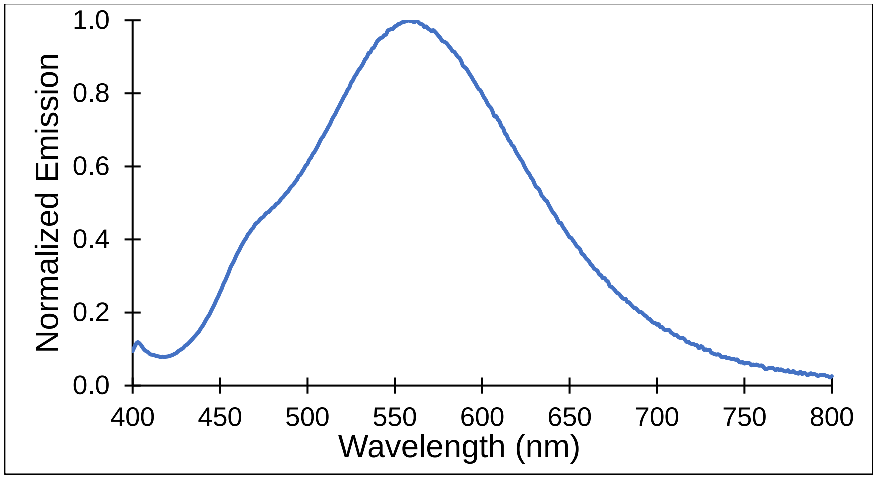

Figure S37. Fluorescence spectrum of PC **2** in DMAc excited at 360 nm.

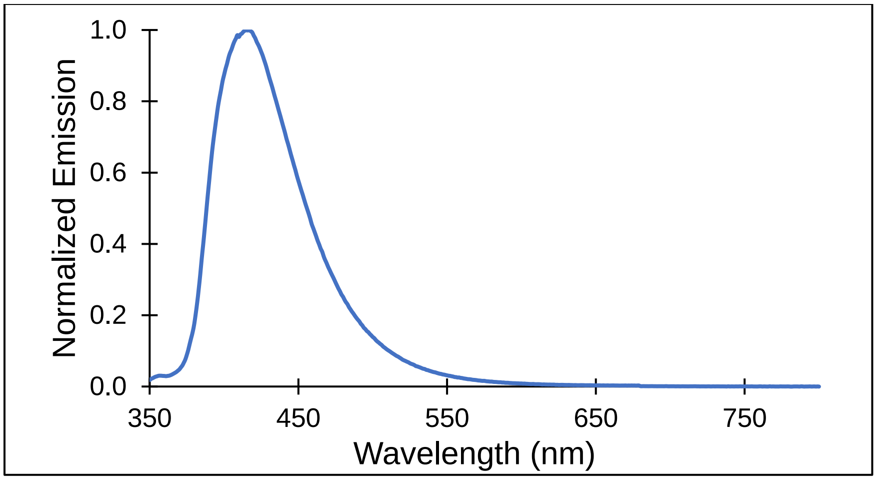

Figure S38. Fluorescence spectrum of PC **3** in DMAc excited at 339 nm.

Solvatochromism

PCs **1**, **1a**, and **1c** were dissolved in increasingly polar solvents and their $\lambda_{max,em}$ measured on FS5 spectrofluorometer. The spectra for each solvent were overlaid to observe the impact of solvent polarity on $\lambda_{max,em}$.

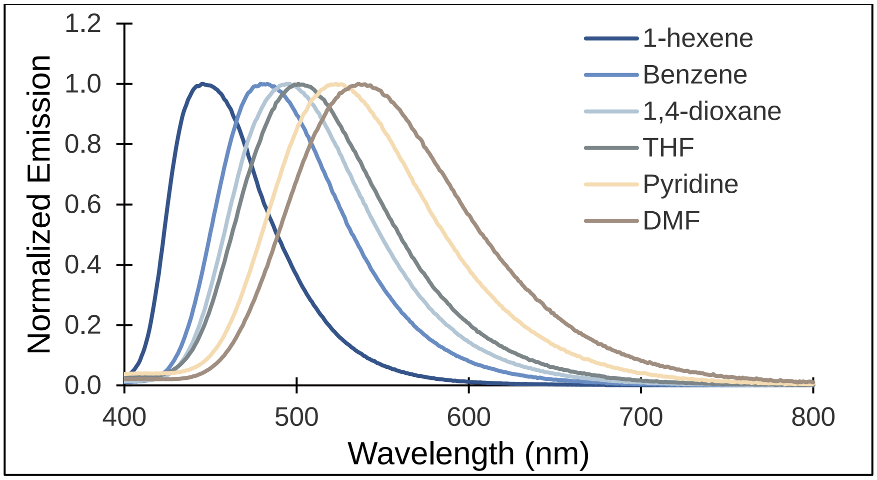

Figure S39. Overlaid emission spectra of PC **1** in different solvents, excited at 340 nm.

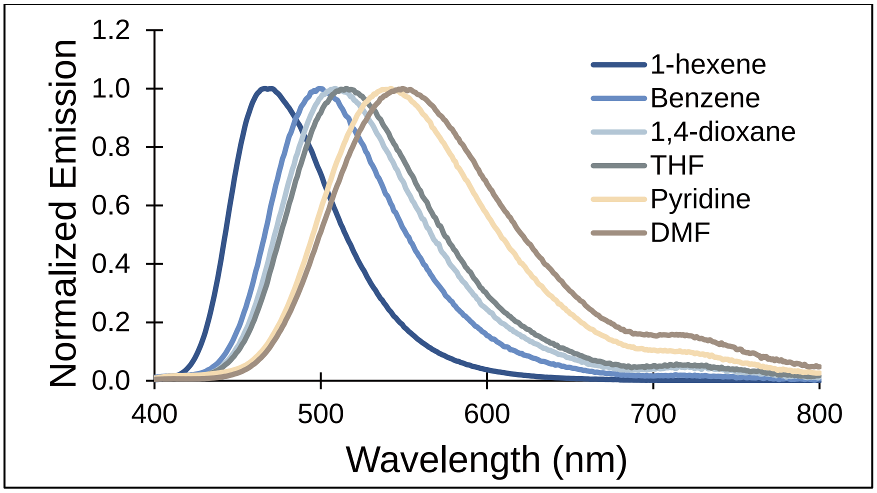

Figure S40. Overlaid emission spectra of PC **1a** in different solvents, excited at 367 nm.

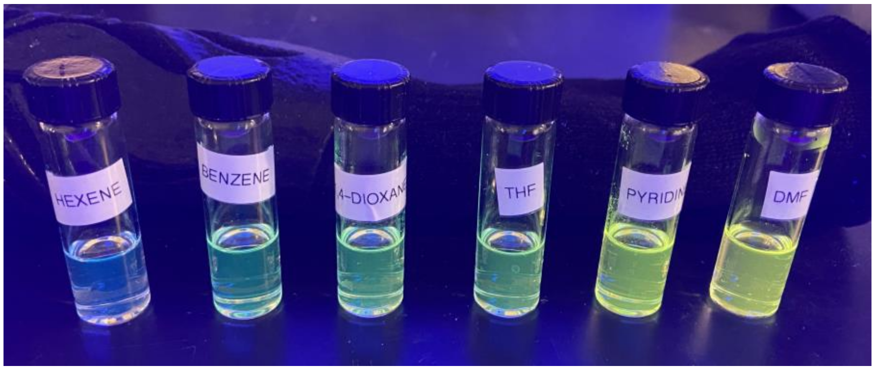

Figure S41. Fluorescence of PC **1a** irradiated by 365 nm light in different solvents.

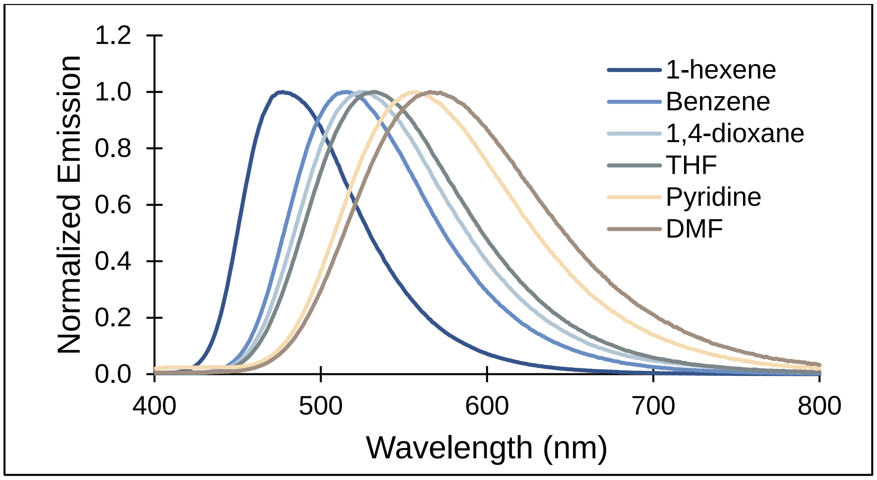

Figure S42. Overlaid emission spectra of PC **1c** in different solvents, excited at 343 nm.

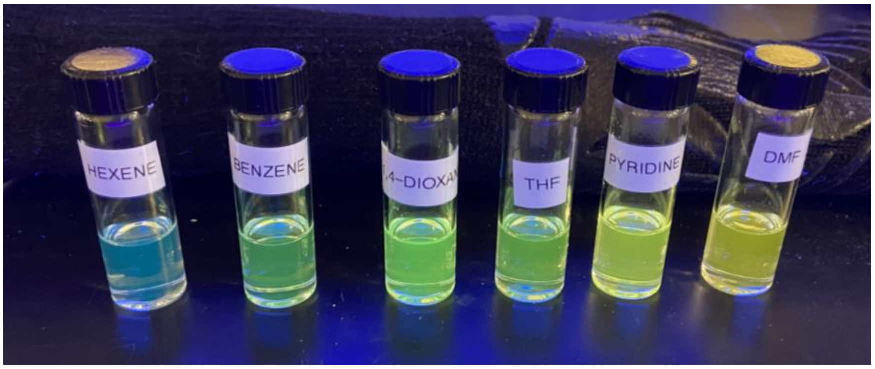

Figure S43. Fluorescence of PC **1c** irradiated by 365 nm light in different solvents.

Cyclic Voltammetry

PCs **1a**, **1b**, **1c**, **1d**, **2**, and **3** were analyzed at 100, 80, 50, and 20 mV/s scan rate with 5 cycles in N_2 sparged DMAc. PC **1** was scanned at 100 mV/s for 5 cycles in N_2 sparged DMAc.

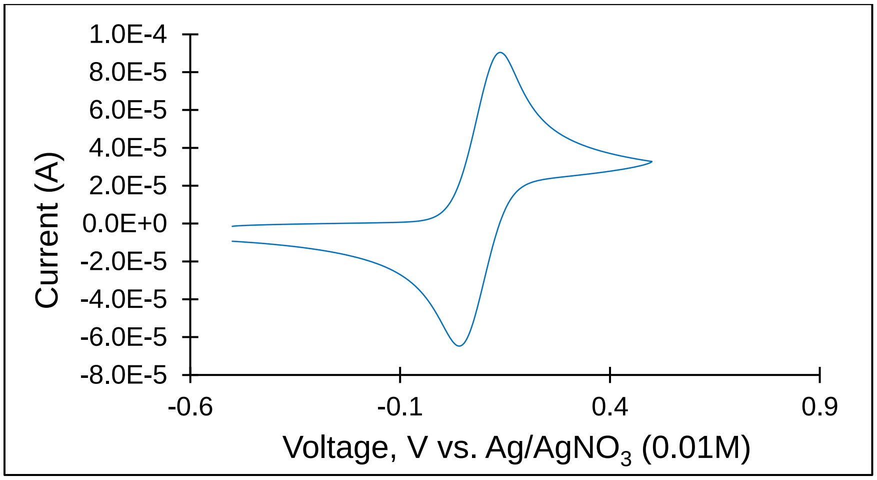

Figure S44. Cyclic voltammogram of PC **1** in DMAc at scan rate 100 mV/s.

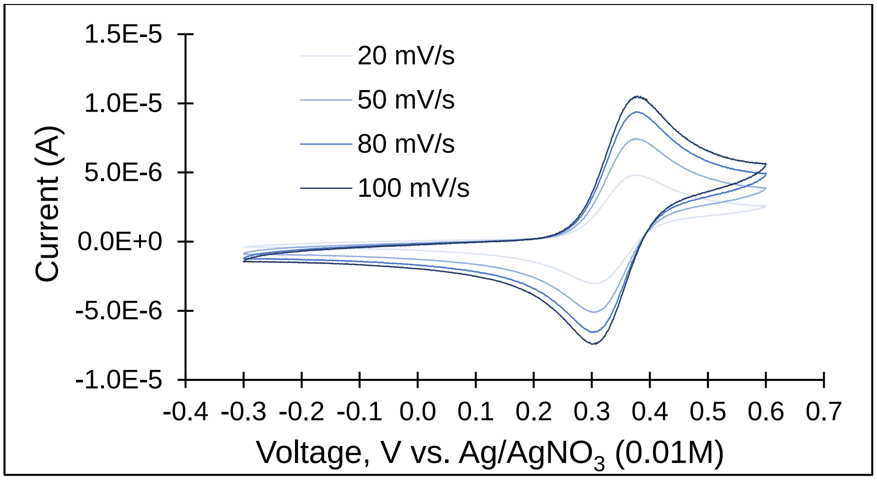

Figure S45. Cyclic voltammograms of PC **1a** in DMAc at different scan rates.

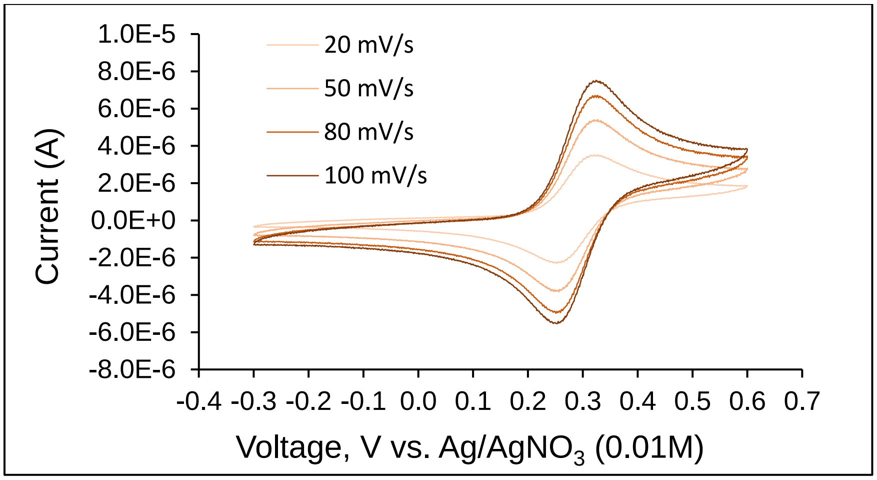

Figure S46. Cyclic voltammograms of PC **1b** in DMAc at different scan rates.

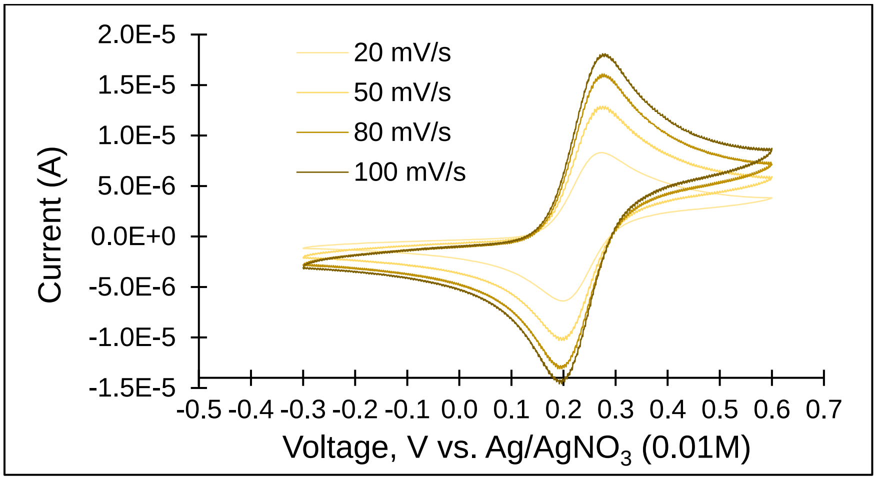

Figure S47. Cyclic voltammograms of PC **1c** in DMAc at different scan rates.

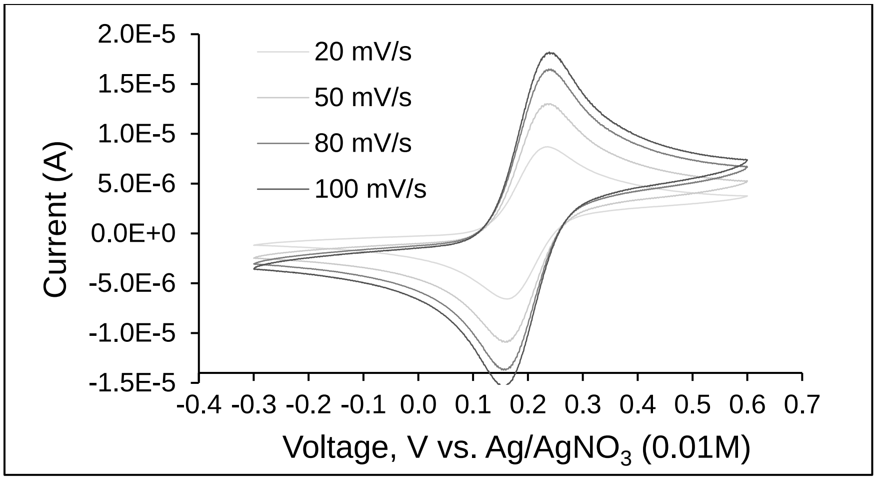

Figure S48. Cyclic voltammograms of PC **1d** in DMAc at different scan rates.

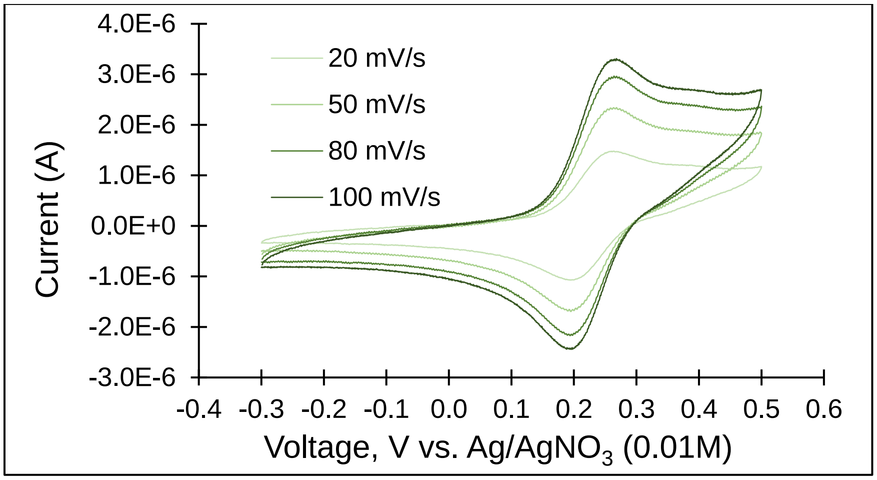

Figure S49. Cyclic voltammograms of PC **2** in DMAc at different scan rates.

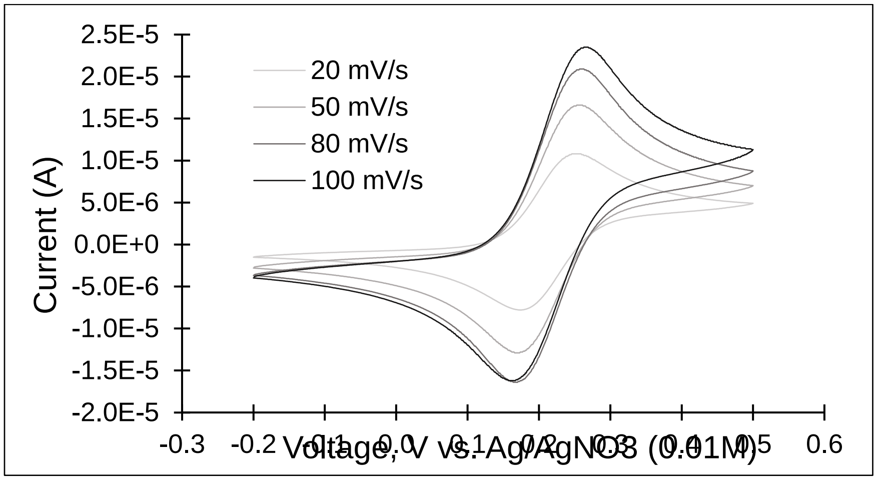

Figure S50. Cyclic voltammograms of PC **3** in DMAc at different scan rates.

Redox calculations

$E_{1/2}$ was taken in reference to $Ag/AgNO_3$ so the values were converted to reference SCE by adding 0.298 V to $E_{1/2}$ measured.

$E_{S1}°*$ was determined through utilizing the following equations where h is Planck's constant and c is the speed of light.

$$E_{S1} = \frac{hc}{\lambda_{max,em}} \quad \textbf{(Eqn. 1)}$$

$$E_{S1}°^{*} = E_{1/2} - E_{S1} \quad \textbf{(Eqn. 2)}$$

POLYMERIZATION DATA

*Control Experiments for O-ATRP of MMA with PC **1a***

Control experiments revealed low conversions or uncontrolled polymerizations when one component of the reaction mixture was absent.

Table S1: Control polymerizations in DMAc with PC **1a**[a]

Conditions	Conversion (%)[b]	$M_{n,exp}$ (kDa)[c]	$Đ$[c]	I^* (%)[d]
**Regular**	39.4	6.20	1.31	68
**No PC**	23.1	3.60	1.66	71
**No initiator**	47.6	6.03	2.47	83
**Dark**	0	-	-	-

[a]Ratio of [MMA]:[DBMM]:[PC] is [1000]:[10]:[1]. 1.0 mL of anhydrous DMAc was used as the solvent with 1.0 mL of MMA under N_2. Time points taken at 8 hours. [b]Calculated by ^1H NMR. [c]Determined by GPC with multiangle light scattering. [d]I^* calculated through $[M_{n,exp}]/[M_{n,theo}]$, where $M_{n,theo}$ is the theoretical number average molecular weight calculated by conversion.

General procedure for O-ATRP of MMA with white LED lights

A 20 mL scintillation vial was charged with PC and a stir bar then transferred to a nitrogen-atmosphere glove box. To the scintillation vial, 1.0 mL solvent and 1.0 mL MMA was added. After MMA was added, the scintillation vial was capped and placed under a light beaker to keep them completely in the dark. 18 microliters of DBMM was added to the scintillation vial and immediately capped and placed into an on light beaker. Polymerizations were stirred for the whole duration. For time points, 0.1 mL aliquots were taken out and injected into a vial with 0.4 mL of 250 ppm butylated hydroxytoluene (BHT) $CDCl_3$. ^1H NMR was utilized to determine conversion. Aliquots were dried down and re-dissolved in HPLC grade THF for analysis through gel permeation chromatography (GPC) equipped with multi-angle light scattering.

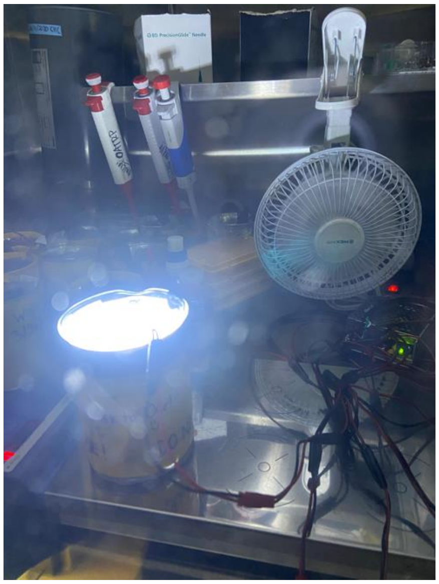

Figure S51. Photograph of white LED polymerization set up.

General procedure for O-ATRP of MMA with 54 W UV light

The same procedure as above was utilized for UV light polymerizations except a 54 W nail curing UV light was used instead of white LED light beakers.

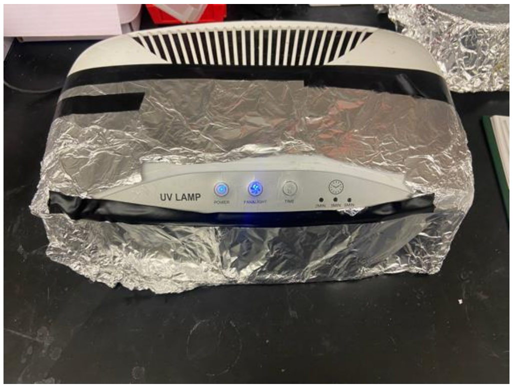

Figure S52. Photograph of 54 W UV light used for UV light polymerizations.

Results of O-ATRP irradiated with 54 W UV light in DMAc

Table S2: Results of O-ATRP catalyzed by PCs **1**, **1a**, **1b**, **1c**, and **1d** in DMAc[a,b]

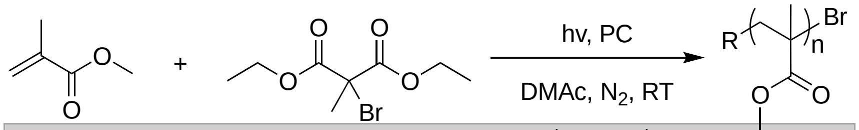

PC	Conversion (%)[c]	$M_{n,exp}$ (kDa)[d]	$Đ$[d]	I^* (%)[e]
**1**	73.9	9.88	1.20	77
**1a**	30.1	4.24	1.29	77

**1b**	30.1	4.33	1.24	75
**1c**	17.4	4.63	1.14	43
**1d**	41.9	5.14	1.23	86

[a]Ratio of [MMA]:[DBMM]:[PC] is [1000]:[10]:[1]. 1.0 mL of anhydrous DMAc was used as the solvent with 1.0 mL of MMA, irradiated with 54 W UV light for 8 hours under N_2. [b]R is the initiator fragment (diethyl 2-methylmalonate). [c]Calculated by ^{1}H NMR. [d]Determined by GPC with multiangle light scattering. [e]I^* calculated through [$M_{n,exp}$]/[$M_{n,theo}$], where $M_{n,theo}$ is the theoretical number average molecular weight calculated by conversion.

O-ATRP irradiated in both visible and 54 W UV light to compare HLCT and CT PCs

Table S3: Results of O-ATRP catalyzed by PhPhenO, PC **1**, and PC **3**[a,b]

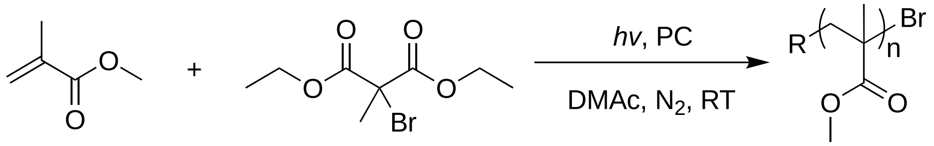

	UV Light[c]				White Light[d]			
PC	Conv. (%)[e]	$M_{n,exp}$ (kDa)[f]	$Đ$[f]	I^* (%)[g]	Conv. (%)[e]	$M_{n,exp}$ (kDa)[f]	$Đ$[f]	I^* (%)[g]
**PhPhenO**	49.7	7.80	1.95	22	-	-	-	-
**PC 1**	49.7	12.9	1.82	41	97.5	14.8	1.26	68
**PC 3**	50.0	23.0	1.70	23	50.0	81.5	1.62	6

[a]Ratio of [MMA]:[DBMM]:[PC] is [1000]:[10]:[1] in DMAc and irradiated for 22 hours. [b]R is the initiator fragment (diethyl 2-methylmalonate).[c]1.0 mL of MMA irradiated with 54 W UV light under N_2. [d]1.0 mL of MMA irradiated with white LED beakers under N_2. [e]Calculated by ^{1}H NMR. [f]Determined by GPC with multiangle light scattering. [g]I^* calculated through [$M_{n,exp}$]/[$M_{n,theo}$], where the $M_{n,theo}$ is the theoretical number average molecular weight calculated by conversion.

*Kinetic polymerizations of PCs **1a**, **1b**, **1c**, and **1d** in ethyl acetate*

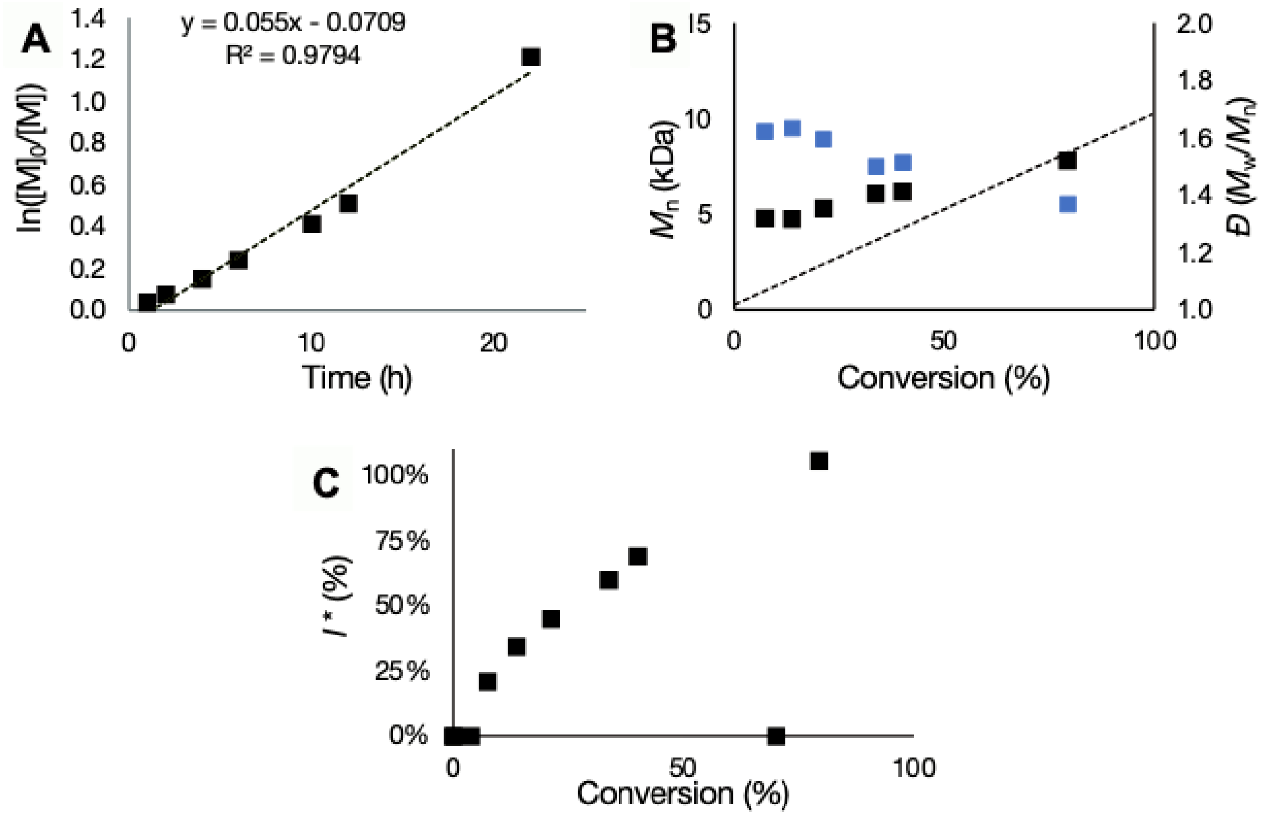

Figure S53. O-ATRP data of MMA with PC **1a**. [MMA]:[DBMM]:[PC] = [1000]:[10]:[1]; 1.0 mL MMA, 1.0 mL anhydrous ethyl acetate, irradiated with LED beakers for 24 hours. **A.** Pseudo-first order kinetics plot. **B.** growth of polymer molecular weight (black) and dispersity (blue) as a function of monomer conversion. The black dashed line represents $M_{n,theo}$. **C.** Initiator efficiency as a function of monomer conversion.

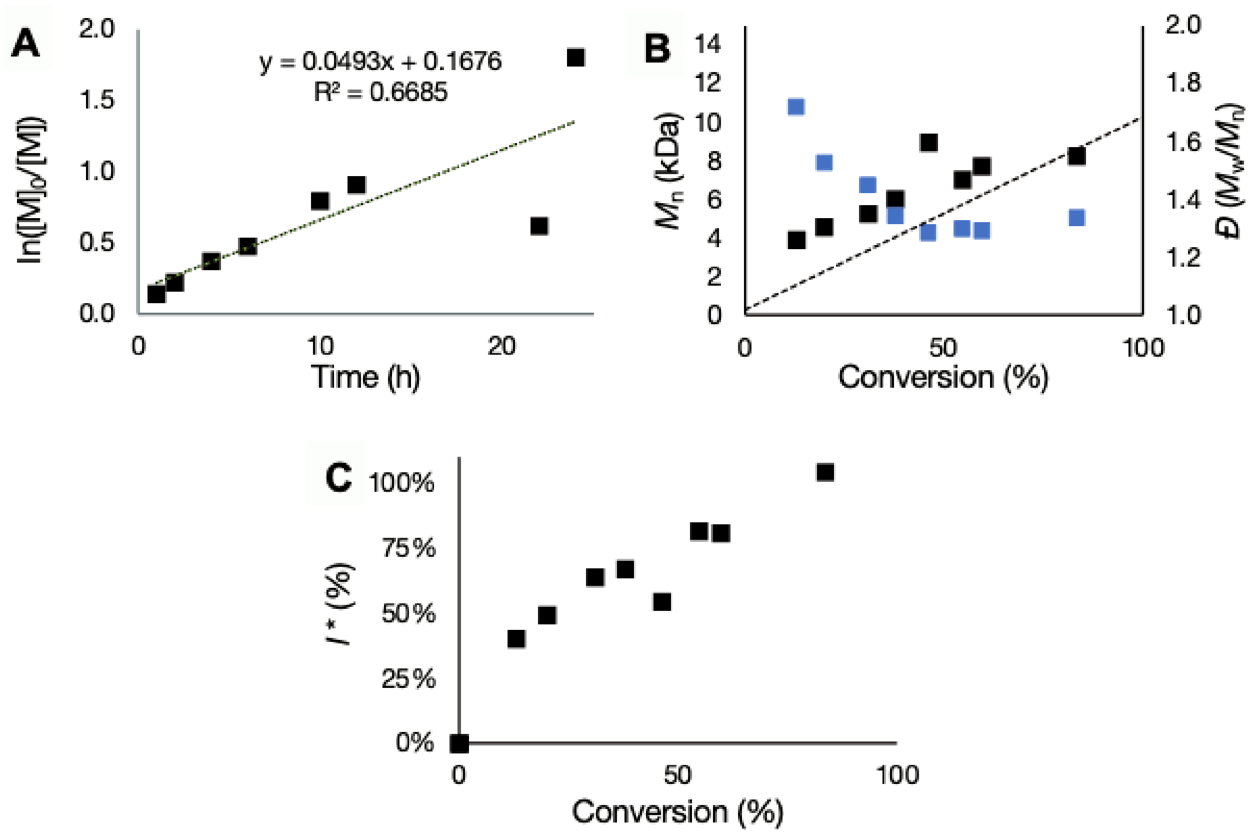

Figure S54. O-ATRP data of MMA with PC **1b**. [MMA]:[DBMM]:[PC] = [1000]:[10]:[1]; 1.0 mL MMA, 1.0 mL anhydrous ethyl acetate, irradiated with LED beakers for 24 hours. **A.** Pseudo-first order kinetics plot. **B.** growth of polymer molecular weight (black) and dispersity (blue) as a function of monomer conversion. The black dashed line represents $M_{n,theo}$. **C.** Initiator efficiency as a function of monomer conversion.

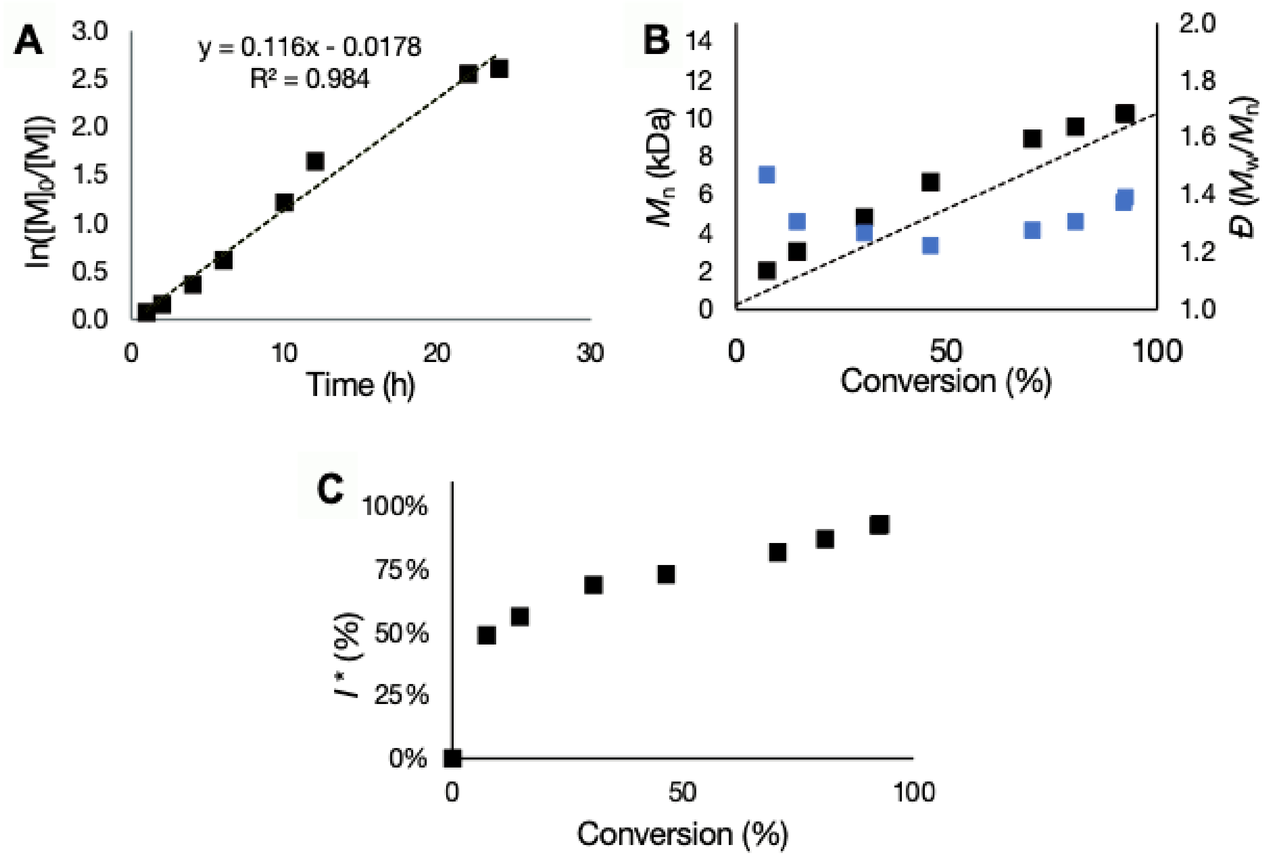

Figure S55. O-ATRP data of MMA with PC **1c**. [MMA]:[DBMM]:[PC] = [1000]:[10]:[1]; 1.0 mL MMA, 1.0 mL anhydrous ethyl acetate, irradiated with LED beakers for 24 hours. **A.** Pseudo-first order kinetics plot. **B.** growth of polymer molecular weight (black) and dispersity (blue) as a function of monomer conversion. The black dashed line represents $M_{n,theo}$ **C.** Initiator efficiency as a function of monomer conversion.

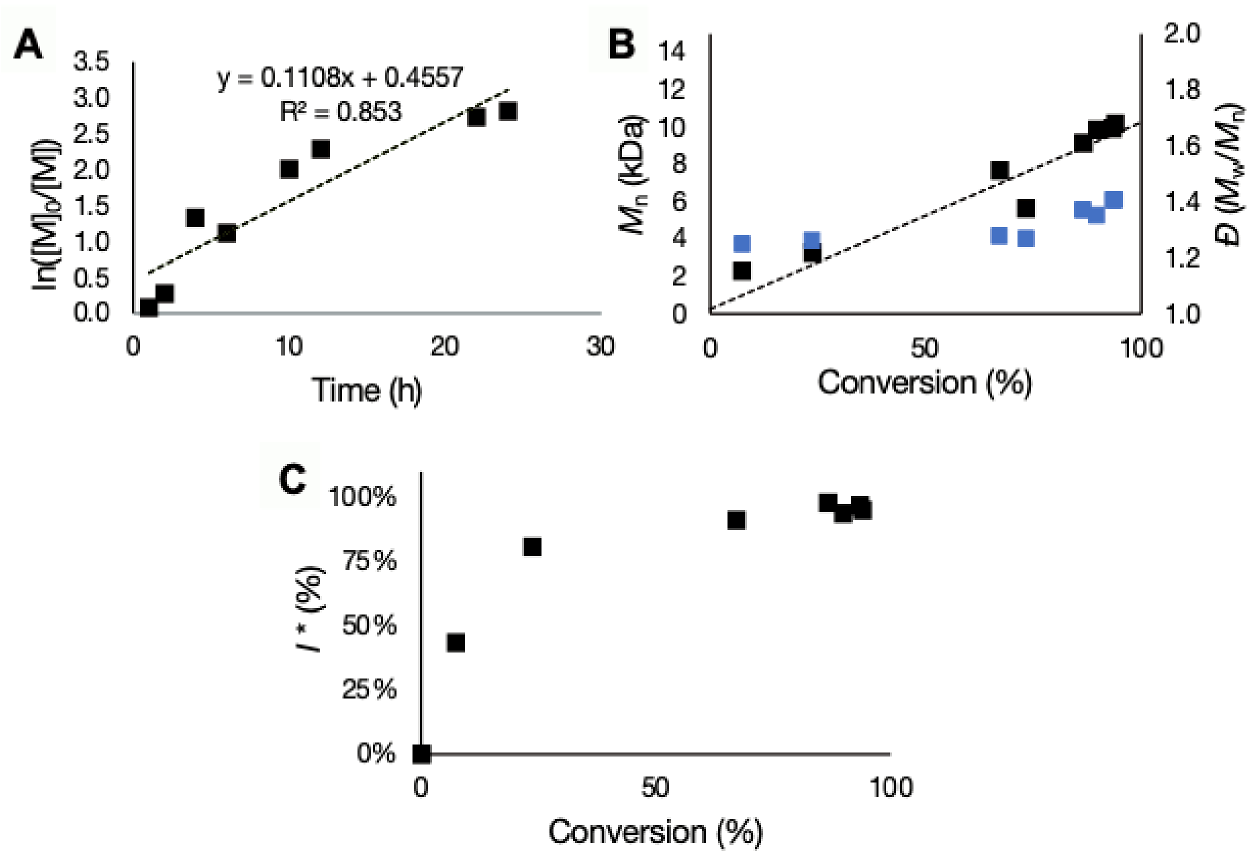

Figure S56. O-ATRP data of MMA with PC **1d**. [MMA]:[DBMM]:[PC] = [1000]:[10]:[1]; 1.0 mL MMA, 1.0 mL anhydrous ethyl acetate, irradiated with LED beakers for 24 hours. **A.** Pseudo-first order kinetics plot. **B.** growth of polymer molecular weight (black) and dispersity (blue) as a function of monomer conversion. The black dashed line represents $M_{n,theo}$. **C.** Initiator efficiency as a function of monomer conversion.

*Kinetic polymerizations of PCs **1a**, **1b**, **1c**, and **1d** in DMAc*

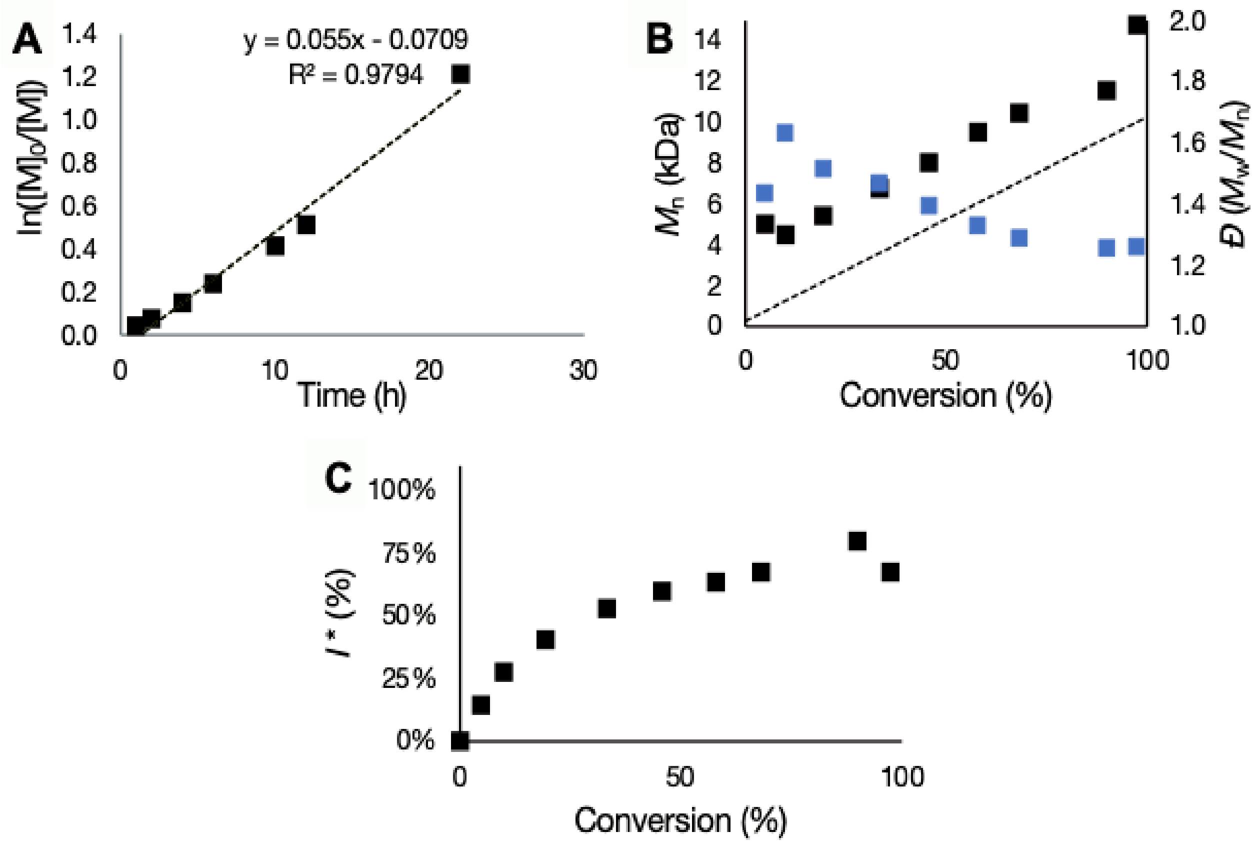

Figure S57. O-ATRP data of MMA with PC **1a**. [MMA]:[DBMM]:[PC] = [1000]:[10]:[1]; 1.0 mL MMA, 1.0 mL anhydrous DMAC, irradiated with LED beakers for 22 hours. **A.** Pseudo-first order kinetics plot. **B.** growth of polymer molecular weight (black) and dispersity (blue) as a function of monomer conversion. The black dashed line represents $M_{n,theo}$. **C.** Initiator efficiency as a function of monomer conversion.

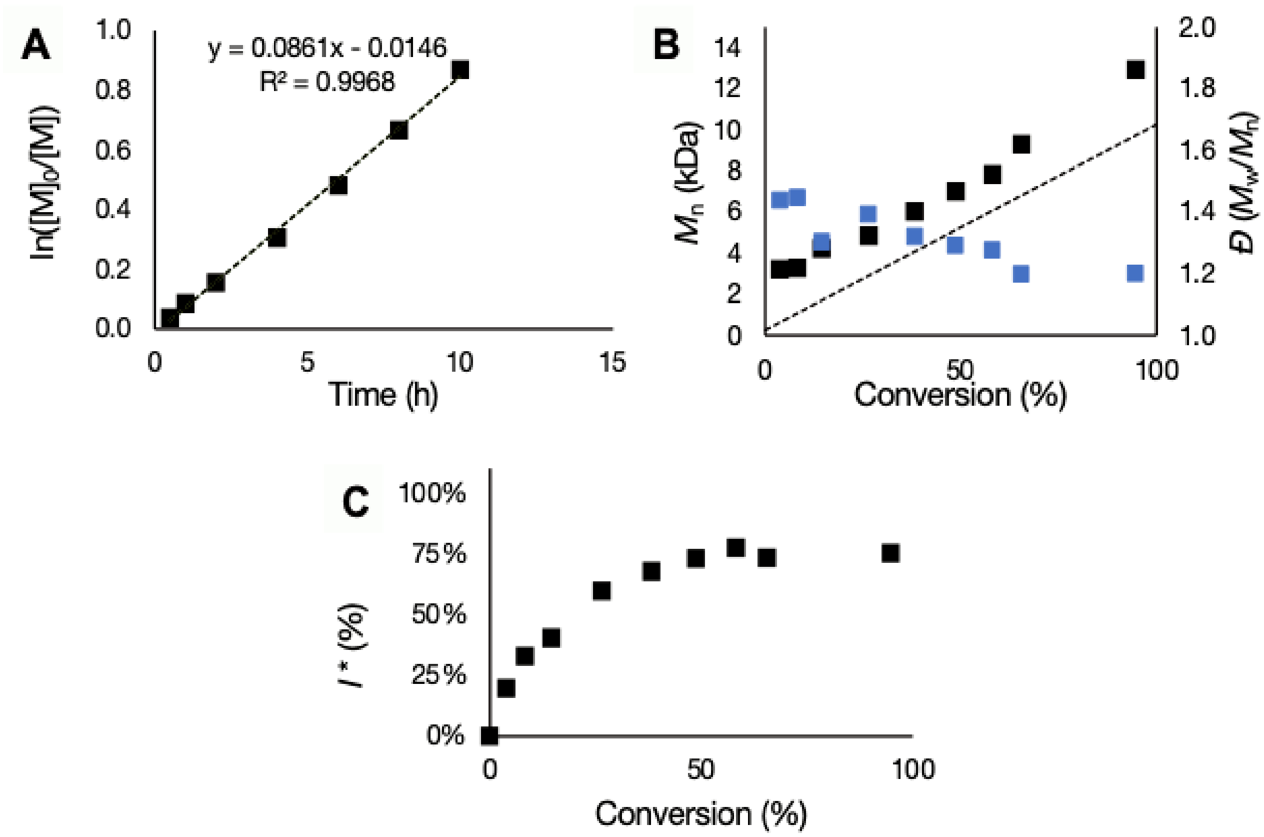

Figure S58. O-ATRP data of MMA with PC **1b**. [MMA]:[DBMM]:[PC] = [1000]:[10]:[1]; 1.0 mL MMA, 1.0 mL anhydrous DMAC, irradiated with LED beakers for 22 hours. **A.** Pseudo-first order kinetics plot. **B.** growth of polymer molecular weight (black) and dispersity (blue) as a function of monomer conversion. The black dashed line represents $M_{n,theo}$. **C.** Initiator efficiency as a function of monomer conversion.

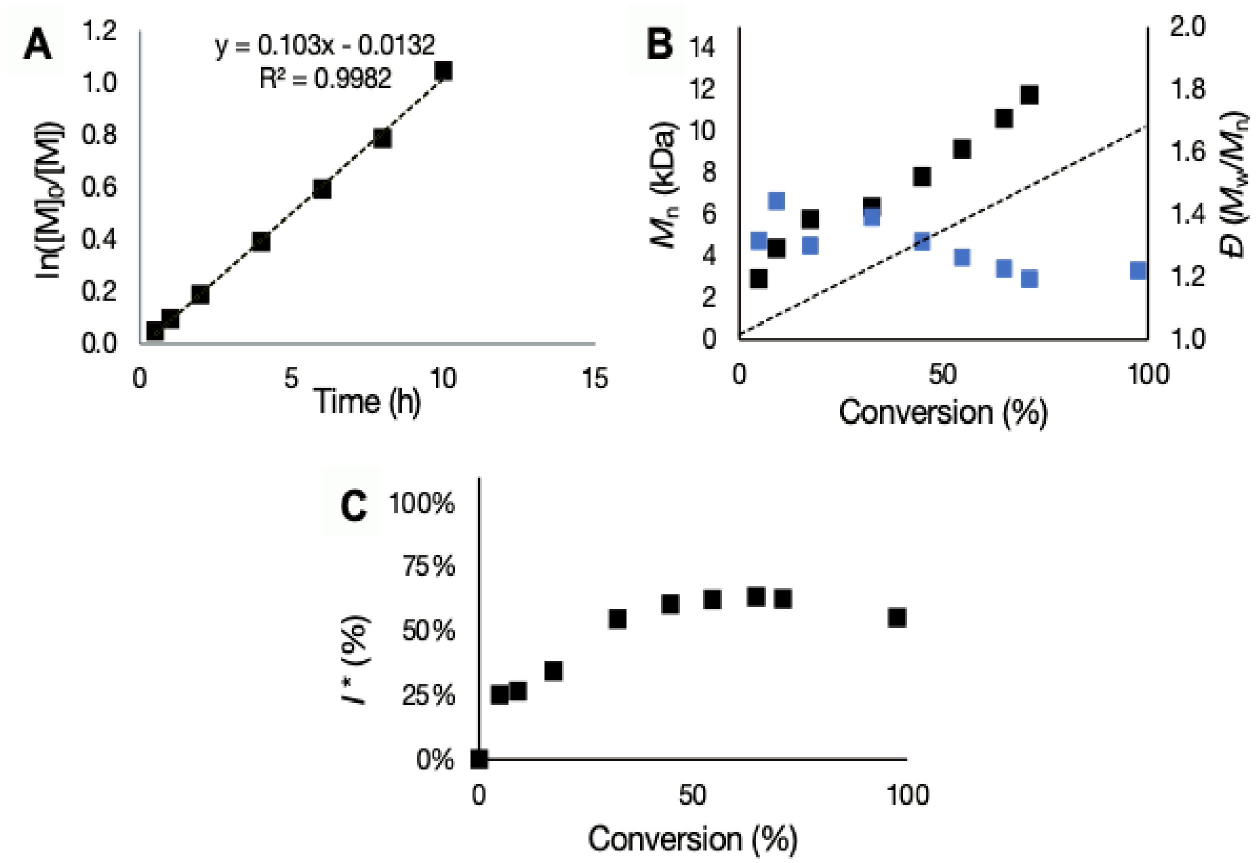

Figure S59. O-ATRP data of MMA with PC **1c**. [MMA]:[DBMM]:[PC] = [1000]:[10]:[1]; 1.0 mL MMA, 1.0 mL anhydrous DMAC, irradiated with LED beakers for 22 hours. **A.** Pseudo-first order kinetics plot. **B.** growth of polymer molecular weight (black) and dispersity (blue) as a function of monomer conversion. The black dashed line represents $M_{n,theo}$. **C.** Initiator efficiency as a function of monomer conversion.

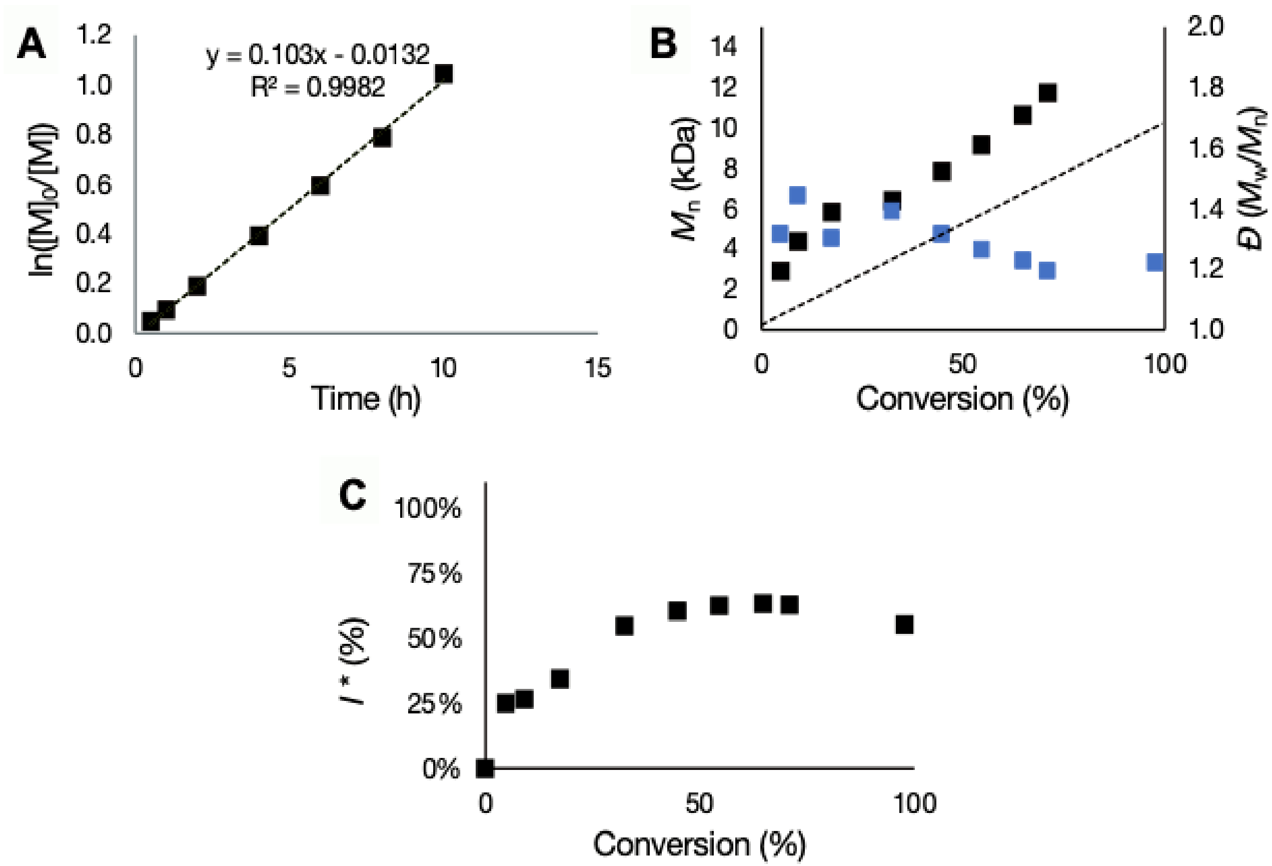

Figure S60. O-ATRP data of MMA with PC **1d**. [MMA]:[DBMM]:[PC] = [1000]:[10]:[1]; 1.0 mL MMA, 1.0 mL anhydrous DMAC, irradiated with LED beakers for 22 hours. **A.** Pseudo-first order kinetics plot. **B.** growth of polymer molecular weight (black) and dispersity (blue) as a function of monomer conversion. The black dashed line represents $M_{n,theo}$. **C.** Initiator efficiency as a function of monomer conversion.

COMPUTATIONAL DETAILS

All density functional theory (DFT) calculations were carried out in *Gaussian 16* version C01.[35] *Pymol*[36] was employed to generate all of the molecular graphics. The isovalue used for molecular orbitals was set to 0.02. The long range separated dispersion-corrected ωB97X-D[37,38] functional and Pople's double-ζ valence 6-31+G(d,p)[39-43] basis set were employed to perform geometry optimizations of all stationary points. The hybrid meta-GGA M06-2X[44] functional applied with Grimme's D3 dispersion[45] in conjunction with the def2-SVPD[46,47] (for C and H) and def2-TZVPD[47,48] (for O, S and N) basis sets were used to perform single-point energy corrections. Solvation effects were accounted for using the SMD[49] variation of the integral equation formalism variant of the polarizable continuum model[50-53] (IEF-PCM) under the presence of solvent, dimethylacematide (smd, solvent=n,n-DiMethylAcetamide).

Vibrational frequencies were computed on the optimized geometries to verify that stationary points were minima on the potential energy surface (PES). The software *Goodvibes*[54] was used to perform quasi-harmonic corrections on the computed vibrational entropies, which follows Grimme's quasi-harmonic approximation (QHA) model[55] with a frequency cut-off value of 100 cm^{-1}. Also, a standard state correction to 1 M solution from gas phase at 1 atm (option "-c 1") was employed.

Single point time-dependent density functional theory (TD-DFT) calculations using the optimized geometries were performed at the (SMD=n,n-DiMethylAcetamide) ωB97X-D/6-31+G(d,p) level of theory to calculate the predicted percentage contribution of corresponding orbitals involved in the initial photoexcitation. Conformation sampling was performed manually. Only orbital contribution of ≥10% were considered.

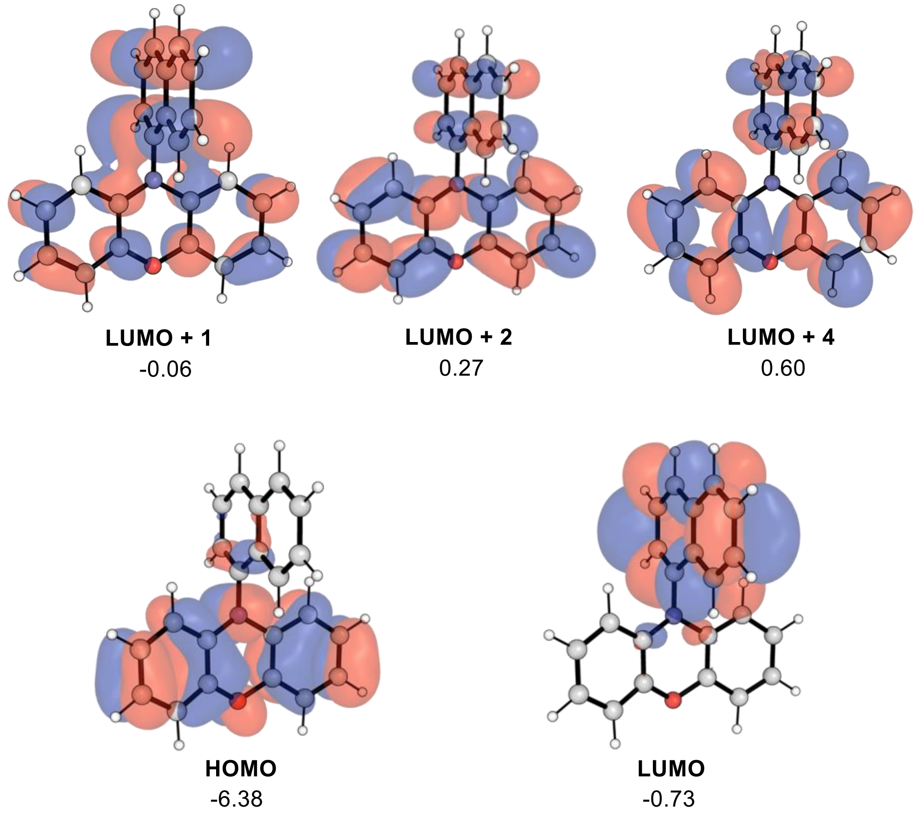

Orbital Contribution	Percent Contribution
HOMO ⟶ LUMO + 1	16%
HOMO ⟶ LUMO + 2	27%
HOMO ⟶ LUMO + 4	45%

Figure S61. Visualization of molecular orbitals and computed molecular orbital transitions for the S_0 -> S_3 excitation of PC 1. The computed molecular orbitals calculated at the (SMD=n,n-DiMethylAcetamide) M06-2x(D3)/def2-TZVPD(def2-SVPD for C & H)//(SMD=n,n-DiMethylAcetamide) ωB97X-D/6-31+G** level of theory in eV units are shown in black.

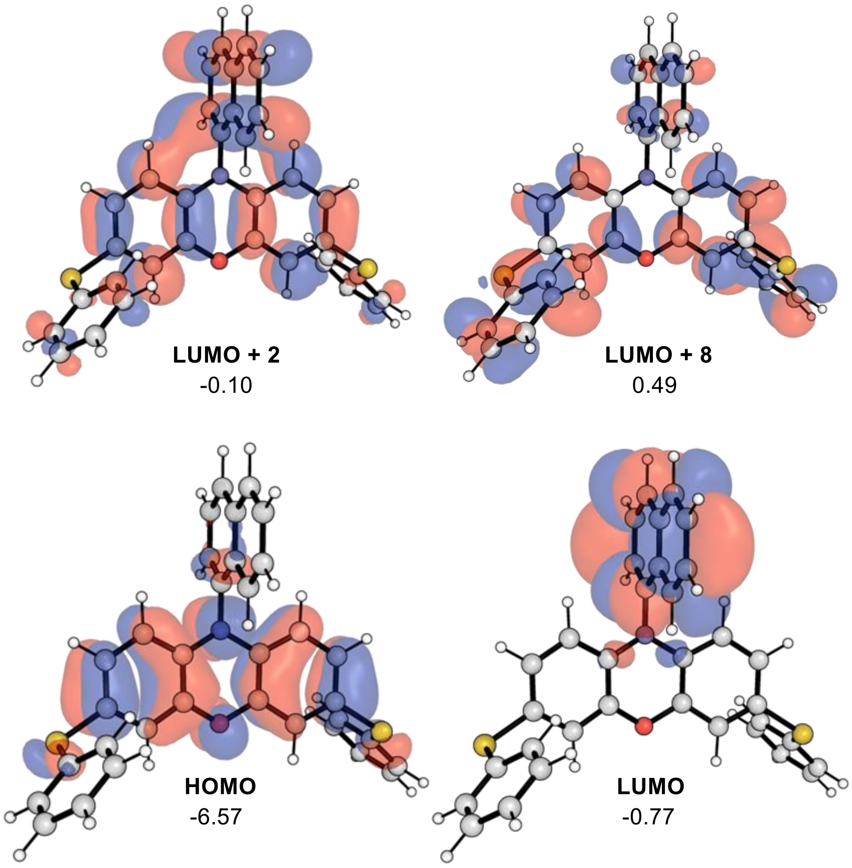

Orbital Contribution	Percent Contribution
HOMO ⟶ LUMO + 2	58%
HOMO ⟶ LUMO + 8	14%

Figure S62. Visualization of molecular orbitals and computed molecular orbital transitions for the S_0 -> S_3 excitation of PC 1a. The computed molecular orbitals calculated at the (SMD=n,n-DiMethylAcetamide) M06-2x(D3)/def2-TZVPD(def2-SVPD for C & H)//(SMD=n,n-DiMethylAcetamide) ωB97X-D/6-31+G** level of theory in eV units are shown in black.

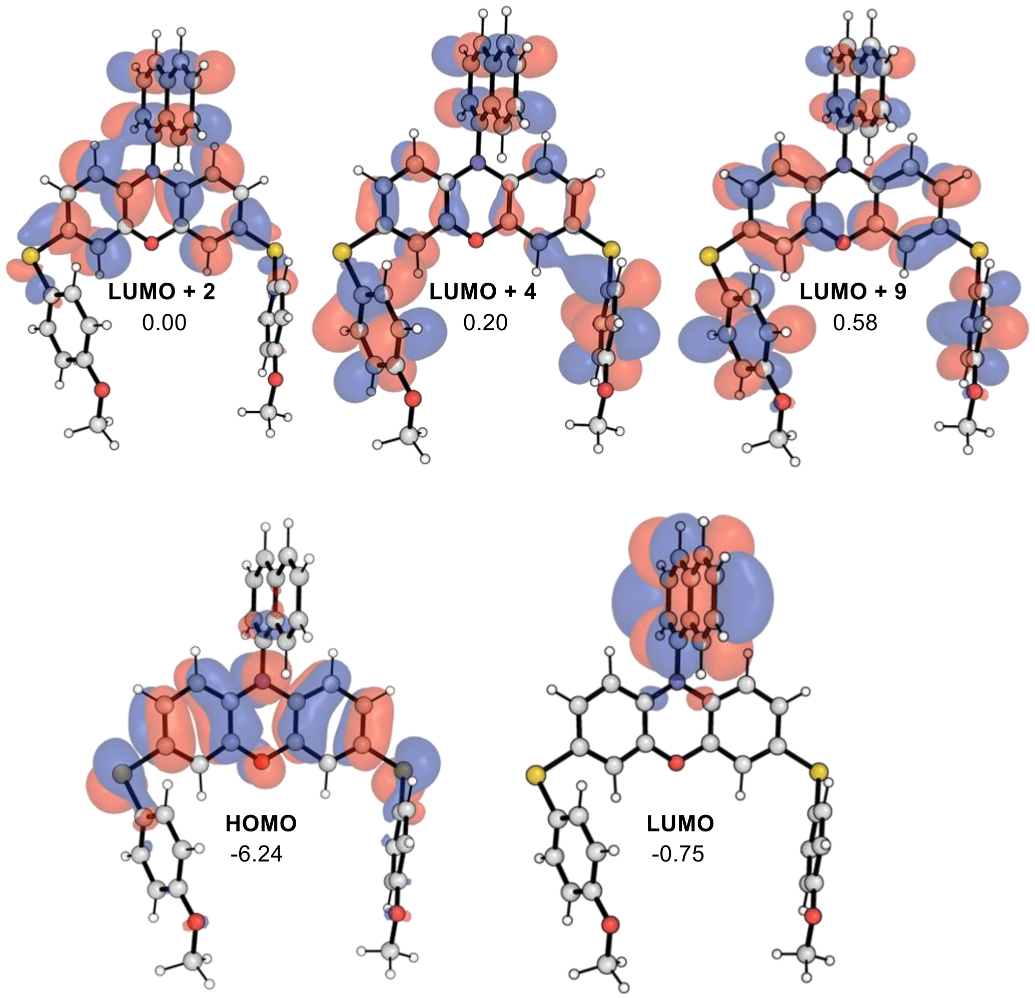

Orbital Contribution	Percent Contribution
HOMO ⟶ LUMO + 2	50%
HOMO ⟶ LUMO + 4	11%
HOMO ⟶ LUMO + 9	13%

Figure S63. Visualization of molecular orbitals and computed molecular orbital transitions for the S_o -> S_3 excitation of PC 1b. The computed molecular orbitals calculated at the (SMD=n,n-DiMethylAcetamide) M06-2x(D3)/def2-TZVPD(def2-SVPD for C & H)//(SMD=n,n-DiMethylAcetamide) ωB97X-D/6-31+G** level of theory in eV units are shown in black.

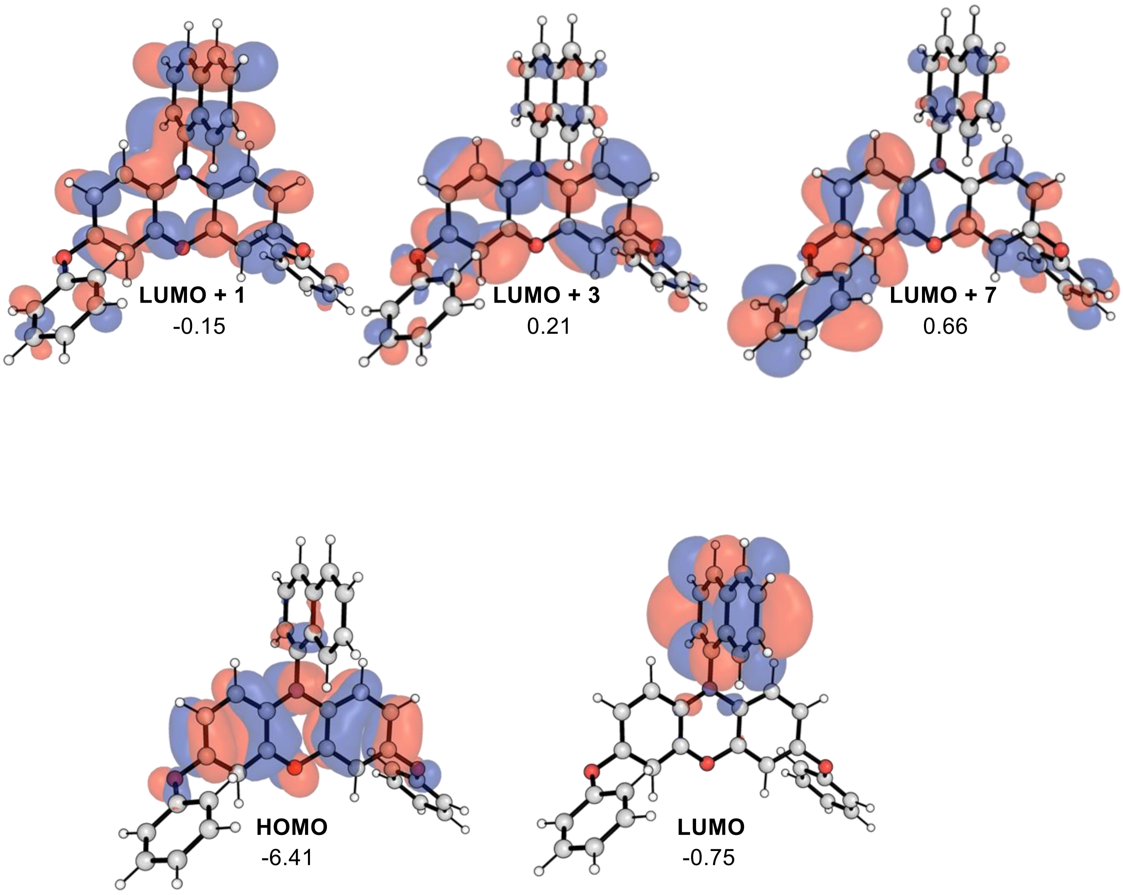

Orbital Contribution	Percent Contribution
HOMO ⟶ LUMO + 1	15%
HOMO ⟶ LUMO + 3	54%
HOMO ⟶ LUMO + 7	10%

Figure S64. Visualization of molecular orbitals and computed molecular orbital transitions for the S_0 -> S_3 excitation of PC 1c. The computed molecular orbitals calculated at the (SMD=n,n-DiMethylAcetamide) M06-2x(D3)/def2-TZVPD(def2-SVPD for C & H)//(SMD=n,n-DiMethylAcetamide) ωB97X-D/6-31+G** level of theory in eV units are shown in black.

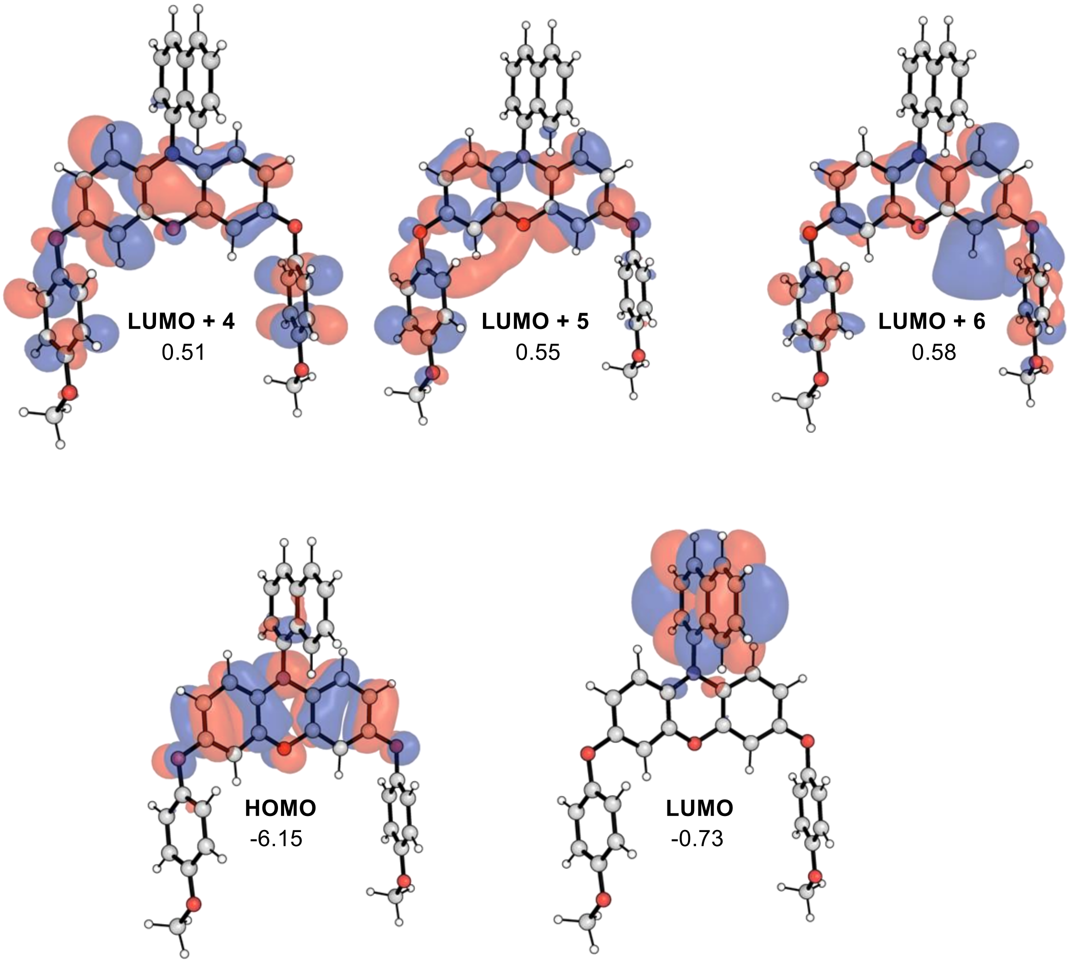

Orbital Contribution	Percent Contribution
HOMO → LUMO + 4	14%
HOMO → LUMO + 5	31%
HOMO → LUMO + 6	25%

Figure S65. Visualization of molecular orbitals and computed molecular orbital transitions for the S_0 -> S_3 excitation of PC 1d. The computed molecular orbitals calculated at the (SMD=n,n-DiMethylAcetamide) M06-2x(D3)/def2-TZVPD(def2-SVPD for C & H)//(SMD=n,n-DiMethylAcetamide) ωB97X-D/6-31+G** level of theory in eV units are shown in black.

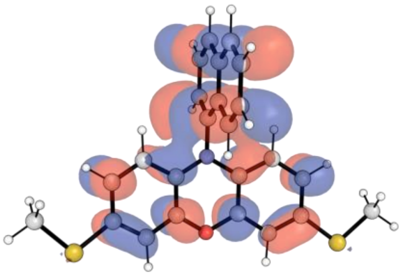

LUMO + 1
-0.08

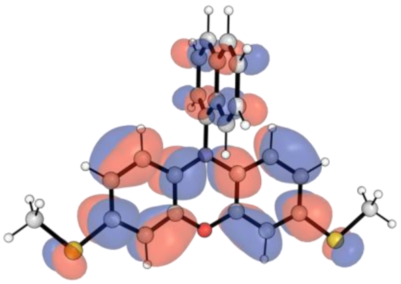

LUMO + 2
0.18

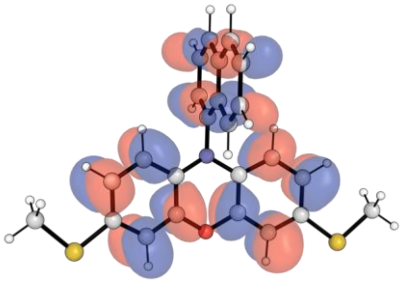

LUMO + 4
0.47

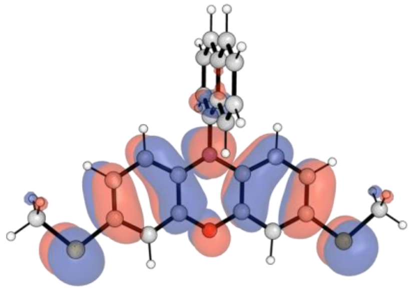

HOMO
-6.09

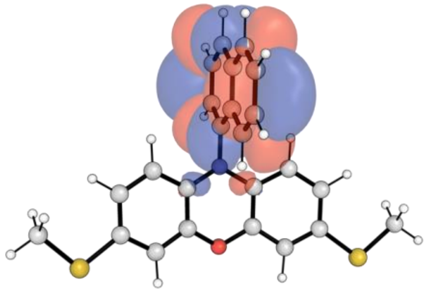

LUMO
-0.74

Orbital Contribution	Percent Contribution
HOMO ⟶ LUMO + 1	15%
HOMO ⟶ LUMO + 2	54%
HOMO ⟶ LUMO + 4	10%

Figure S66. Visualization of molecular orbitals and computed molecular orbital transitions for the S_0 -> S_3 excitation of PC 2. The computed molecular orbitals calculated at the (SMD=n,n-DiMethylAcetamide) M06-2x(D3)/def2-TZVPD(def2-SVPD for C & H)//(SMD=n,n-DiMethylAcetamide) ωB97X-D/6-31+G** level of theory in eV units are shown in black.

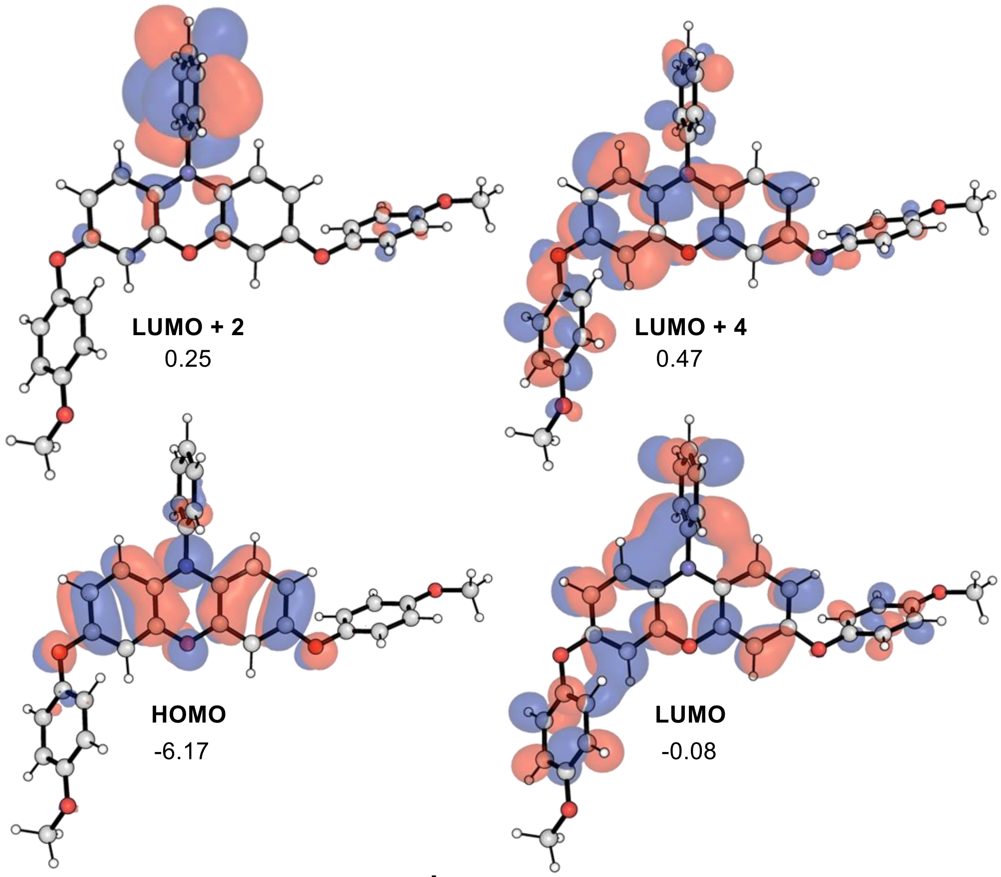

Orbital Contribution	Percent Contribution
HOMO → LUMO + 2	24%
HOMO → LUMO + 4	50%

Figure S67. Visualization of molecular orbitals and computed molecular orbital transitions for the S_0 -> S_3 excitation of PC 3. The computed molecular orbitals calculated at the (SMD=n,n-DiMethylAcetamide) M06-2x(D3)/def2-TZVPD(def2-SVPD for C & H)//(SMD=n,n-DiMethylAcetamide) ωB97X-D/6-31+G** level of theory in eV units are shown in black.

Legend

E_SPC = Absolute energy obtained using single-point energy corrections
ZPE = Absolute zero-point energy
H_SPC = Absolute enthalpy corrected using E_SPC
T.S = Temperature times entropy with no correction
T.qh_S = Absolute temperature times the quasi-harmonic corrected entropy value
G(T)_SPC = Absolute Gibbs free energy corrected with DE_SPC
qh-G(T)_SPC = Absolute Gibbs free energy calculated using DE_SPC and quasi-harmonic corrected entropy.

Table S4: Compiled thermochemical data computed at the (SMD= n,n-DiMethylAcetamide) M06-2x(D3)/def2-TZVPD(def2-SVPD for C & H)// (SMD= n,n-DiMethylAcetamide) ωB97X-D/6-31+G**

Structure	E_SPC	E	ZPE	H_SPC	T.S	T.qh-S	G(T)_SPC	qh-G(T)_SPC
1d	-1817.475081	-1818.456678	0.547467	-1816.892961	0.102876	0.092715	-1816.995836	-1816.985676
1d_triplet	-1817.381413	-1818.364162	0.543552	-1816.802923	0.103382	0.093893	-1816.906305	-1816.896816
1c	-1588.506835	-1589.468081	0.481776	-1587.995555	0.09151	0.082625	-1588.087065	-1588.078181
1c_triplet	-1588.411112	-1589.373735	0.477797	-1587.903467	0.091673	0.083552	-1587.995139	-1587.987019
3	-1663.991957	-1664.854121	0.499846	-1663.459988	0.097647	0.088046	-1663.557636	-1663.548035
3_triplet	-1663.888346	-1664.752901	0.497409	-1663.358505	0.097958	0.088981	-1663.456463	-1663.447486
1b	-2463.416762	-2464.418233	0.541906	-2462.838759	0.10789	0.096396	-2462.946649	-2462.935155
1b_triplet	-2463.321103	-2464.321909	0.537943	-2462.746867	0.107286	0.096890	-2462.854153	-2462.843757
2	-1851.373419	-1852.057597	0.369449	-1850.979396	0.076596	0.071704	-1851.055991	-1851.0511

2_triplet	-1851.278134	-1851.961993	0.365237	-1850.888003	0.078728	0.073595	-1850.966732	-1850.961599
1	-976.459462	-977.102627	0.310997	-976.130519	0.060906	0.05792	-976.191425	-976.188438
1_triplet								
1a	-2234.444931	-2235.425672	0.47618	-2233.937868	0.094919	0.085608	-2234.032787	-2234.023477
1a_triplet	-976.357455	-977.002087	0.307220	-976.032002	0.062363	0.059481	-976.094365	-976.091483

Molecular XYZ Coordinates

Molecular XYZ Coordinates

69

1d Eopt -1818.456678

C	0.284326	-3.563189	-0.199487
C	-0.346369	-2.334819	-0.400050
C	0.428303	-1.209301	-0.636367
C	1.825280	-1.261872	-0.669731
C	2.431978	-2.500885	-0.461841
C	1.672581	-3.645872	-0.232841
C	1.861835	1.142121	-0.783745
C	0.463359	1.134523	-0.744708
C	-0.275698	2.299755	-0.614004
H	-1.357668	2.233648	-0.591817
C	0.392609	3.521934	-0.527979
C	1.781766	3.560122	-0.571746
C	2.506010	2.376127	-0.694569
H	-1.425476	-2.232790	-0.386186
H	3.513351	-2.580874	-0.485427
H	2.159344	-4.602990	-0.078417
H	2.297393	4.512275	-0.506765
H	3.589257	2.420442	-0.725408
C	3.974568	-0.096676	-0.844223
C	4.622241	-0.050976	0.428693
C	4.705355	-0.155227	-2.003473
C	3.902079	0.009695	1.651952
C	6.045592	-0.068462	0.463383
C	6.119937	-0.171437	-1.961613
H	4.183642	-0.188593	-2.955079
C	4.568868	0.050109	2.851893
H	2.816837	0.023200	1.633789
C	6.704755	-0.024794	1.722344
C	6.773835	-0.129122	-0.756562
H	6.680647	-0.217475	-2.889785
C	5.984536	0.032690	2.889465
H	4.008106	0.095621	3.780558
H	7.791231	-0.038321	1.743125
H	7.859741	-0.141497	-0.716644
H	6.497062	0.065343	3.846081
N	2.548107	-0.079684	-0.923068
O	-0.248101	-0.035818	-0.881408
C	-1.603228	4.724771	-0.102726
C	-2.542992	5.011566	-1.079602
C	-1.998926	4.493659	1.214977
C	-3.900111	5.072012	-0.751466
H	-2.220864	5.188246	-2.101158
C	-3.344537	4.546602	1.545871
H	-1.254238	4.270957	1.973166
C	-4.303826	4.835958	0.564808
H	-4.619300	5.300066	-1.529150
H	-3.669197	4.370331	2.566547
C	-1.776298	-4.681241	0.144341

C	-2.570706	-4.962112	-0.956279
C	-2.353536	-4.400539	1.381964
C	-3.962282	-4.963047	-0.834617
H	-2.107364	-5.179661	-1.913802
C	-3.734936	-4.397084	1.509063
H	-1.720820	-4.182384	2.236874
C	-4.547768	-4.676341	0.401554
H	-4.565750	-5.188555	-1.705904
H	-4.201215	-4.180685	2.465081
O	-0.258763	4.726468	-0.434185
O	-0.397363	-4.732697	0.023545
O	-5.592709	4.867468	0.987556
O	-5.885286	-4.647707	0.626496
C	-6.603298	5.154673	0.030754
H	-6.465842	6.150098	-0.407160
H	-6.623564	4.401381	-0.765145
H	-7.547082	5.128206	0.576015
C	-6.753893	-4.904593	-0.468428
H	-6.616278	-4.166557	-1.266967
H	-6.603082	-5.913666	-0.868700
H	-7.766303	-4.822077	-0.072320

61

1c Eopt -1589.468081

C	3.405733	-0.630433	0.875799
C	2.322108	-1.389068	0.434997
C	1.233336	-0.744059	-0.122243
C	1.186067	0.654174	-0.233010
C	2.277397	1.388623	0.228190
C	3.391646	0.750153	0.776021
C	-1.103567	0.466953	-0.960802
C	-0.999283	-0.926623	-0.829776
C	-2.099134	-1.751139	-0.976216
H	-1.986997	-2.825149	-0.869436
C	-3.339187	-1.181689	-1.264914
C	-3.473169	0.188420	-1.407663
C	-2.353967	1.009001	-1.252192
H	2.329523	-2.471643	0.508828
H	2.267735	2.470262	0.153516
H	4.240903	1.329676	1.121949
H	-4.442210	0.619815	-1.634793
H	-2.461523	2.081964	-1.365409
C	-0.045094	2.675180	-0.871215
C	-0.469455	3.412636	0.275471
C	0.273810	3.312222	-2.043058
C	-0.807170	2.787381	1.505524
C	-0.553563	4.830073	0.171837
C	0.187472	4.721387	-2.138551
H	0.592638	2.722002	-2.896822
C	-1.209521	3.540095	2.581328
H	-0.744761	1.706988	1.590601
C	-0.973868	5.579640	1.304452
C	-0.216659	5.461677	-1.056615
H	0.443167	5.209405	-3.073671
C	-1.294257	4.950468	2.481611
H	-1.464971	3.052171	3.517110
H	-1.036494	6.661260	1.220141
H	-0.285328	6.544093	-1.123467
H	-1.613683	5.531199	3.341613
N	0.050950	1.250550	-0.804621
O	0.213092	-1.533271	-0.598695
C	-5.031175	-2.591131	-0.388682
C	-5.957925	-3.595552	-0.675307
C	-4.774014	-2.217046	0.930444
C	-6.626772	-4.229918	0.366475
H	-6.142461	-3.868444	-1.709673
C	-5.451680	-2.865686	1.964019
H	-4.059460	-1.432759	1.155830
C	-6.377007	-3.870836	1.692744
H	-7.345257	-5.011915	0.139136
H	-5.249541	-2.573633	2.990391
C	5.414875	-1.873135	0.675342
C	5.446421	-1.744504	-0.713323
C	6.376803	-2.636663	1.340596
C	6.451841	-2.393561	-1.432190
H	4.703949	-1.149037	-1.233366
C	7.372858	-3.274936	0.609234
H	6.332729	-2.721353	2.421983
C	7.416492	-3.159178	-0.781969
H	6.474215	-2.292069	-2.513302
H	8.118246	-3.867969	1.130960
H	8.194136	-3.660077	-1.349864
H	-6.898402	-4.369759	2.503518
O	-4.434011	-1.998873	-1.473269
O	4.480275	-1.262412	1.473017

63

3 Eopt -1664.854121

C	2.602435	-1.182237	-0.224593
C	1.236290	-1.323402	-0.480858
C	0.432753	-0.200177	-0.526808
C	0.947482	1.087078	-0.309408
C	2.309565	1.201291	-0.047432
C	3.142322	0.078701	-0.006148
C	-1.304520	1.933672	-0.326025
C	-1.762844	0.627409	-0.537142
C	-3.109503	0.304926	-0.499436
H	-3.408809	-0.723044	-0.672152
C	-4.042845	1.312689	-0.251482
C	-3.618508	2.619850	-0.047996
C	-2.258033	2.922624	-0.083558
H	0.804480	-2.303360	-0.654262
H	2.744189	2.180120	0.122327
H	4.198988	0.208756	0.197158

H	-4.347787	3.400704	0.139596
H	-1.941439	3.947133	0.077685
C	0.562764	3.484399	-0.017409
C	0.674941	3.825635	1.331368
C	0.923184	4.393303	-1.008419
C	1.149920	5.085294	1.686511
C	1.399464	5.654182	-0.649302
H	0.828866	4.107542	-2.051805
C	1.512830	6.000111	0.696354
H	1.681214	6.363866	-1.421119
H	1.883483	6.981936	0.975176
N	0.078024	2.192039	-0.397154
O	-0.891719	-0.393234	-0.842030
C	-5.851380	-0.196256	0.008662
C	-6.567234	-0.861806	-0.973081
C	-5.650860	-0.784070	1.258912
C	-7.096087	-2.130655	-0.718981
H	-6.716737	-0.392850	-1.940626
C	-6.165244	-2.046022	1.513454
H	-5.091127	-0.255485	2.024724
C	-6.891750	-2.727951	0.526561
H	-7.655661	-2.632509	-1.499522
H	-6.017218	-2.516691	2.480370
C	4.706390	-2.264807	-0.156684
C	5.378869	-2.524971	1.026406
C	5.412846	-1.981536	-1.325762
C	6.775938	-2.503887	1.058700
H	4.816117	-2.745910	1.928131
C	6.798938	-1.952926	-1.299052
H	4.875766	-1.781015	-2.247964
C	7.489469	-2.213338	-0.106601
H	7.283164	-2.714014	1.992902
H	7.363893	-1.733983	-2.199727
O	-5.397300	1.085490	-0.248010
O	3.324327	-2.348618	-0.183828
O	8.843216	-2.163050	-0.183519
C	9.590908	-2.423909	0.996263
H	9.405772	-3.438096	1.368472
H	10.639429	-2.330275	0.712367
H	9.362238	-1.695180	1.782381
O	-7.356242	-3.954986	0.874113
C	-8.094946	-4.688453	-0.092888
H	-7.489610	-4.896775	-0.982675
H	-8.364457	-5.629344	0.387653
H	-9.007859	-4.156433	-0.384372
H	1.237583	5.353146	2.735084
H	0.389784	3.104114	2.091710

69

1b Eopt -2464.418233

C	0.552654	-3.560862	-0.946128
C	-0.043893	-2.327350	-1.230382
C	0.717732	-1.174855	-1.213002
C	2.090842	-1.204125	-0.924775
C	2.675399	-2.439815	-0.654557
C	1.913356	-3.609326	-0.659498
C	2.090864	1.204150	-0.924727
C	0.717753	1.174917	-1.212956
C	-0.043849	2.327427	-1.230292
H	-1.100488	2.249192	-1.466343
C	0.552720	3.560916	-0.945987
C	1.913422	3.609343	-0.659348
C	2.675444	2.439818	-0.654457
H	-1.100530	-2.249085	-1.466428
H	3.734138	-2.496093	-0.426590
H	2.395212	-4.553897	-0.426699
H	2.395294	4.553895	-0.426507
H	3.734183	2.496067	-0.426483
C	4.188062	-0.000012	-0.536574
C	4.538419	-0.000034	0.847569
C	5.153206	-0.000005	-1.510676
C	3.566276	-0.000039	1.883486
C	5.919636	-0.000051	1.192567
C	6.524111	-0.000022	-1.159783
H	4.852671	0.000013	-2.553992
C	3.953564	-0.000061	3.200987
H	2.511375	-0.000026	1.627526
C	6.286373	-0.000073	2.566067
C	6.897405	-0.000045	0.160265
H	7.275004	-0.000016	-1.943498
C	5.326842	-0.000079	3.547667
H	3.202594	-0.000065	3.985273
H	7.342039	-0.000086	2.823873
H	7.948362	-0.000057	0.436346
H	5.616392	-0.000096	4.594078
N	2.810450	0.000006	-0.919334
O	0.082595	0.000043	-1.541573
C	-1.939911	4.586460	-0.301988
C	-3.086838	4.567611	-1.091148
C	-2.035210	4.229033	1.050540
C	-4.322916	4.202137	-0.552967
H	-3.027582	4.834061	-2.142149
C	-3.251830	3.848217	1.590648
H	-1.150630	4.241815	1.680596
C	-4.406047	3.834947	0.792194
H	-5.197024	4.199549	-1.193367
H	-3.329571	3.564944	2.635599
C	-1.939962	-4.586425	-0.302074
C	-3.086926	-4.567493	-1.091177
C	-2.035192	-4.229115	1.050489
C	-4.322973	-4.202050	-0.552906
H	-3.027724	-4.833854	-2.142204
C	-3.251781	-3.848327	1.590688

H	-1.150582	-4.241965	1.680502
C	-4.406036	-3.834974	0.792291
H	-5.197112	-4.199395	-1.193264
H	-3.329467	-3.565144	2.635668
O	-5.547105	3.455215	1.411279
C	-6.748226	3.427629	0.649678
H	-6.996166	4.422776	0.264049
H	-6.675244	2.716256	-0.180420
H	-7.529047	3.101622	1.336940
O	-5.547059	-3.455280	1.411467
C	-6.748220	-3.427611	0.649933
H	-6.675277	-2.716165	-0.180105
H	-6.996195	-4.422721	0.264230
H	-7.529000	-3.101657	1.337268
S	-0.377603	-5.086054	-1.006538
S	-0.377511	5.086126	-1.006335

47

2 Eopt -1852.057597

C	3.571082	-1.826465	0.043090
C	2.327454	-2.476148	0.025974
C	1.175474	-1.766075	-0.249876
C	1.204332	-0.386606	-0.504954
C	2.441821	0.249519	-0.479879
C	3.617069	-0.459030	-0.214297
C	-1.204355	-0.386573	-0.504934
C	-1.175534	-1.766041	-0.249864
C	-2.327532	-2.476085	0.025994
H	-2.242122	-3.542035	0.217751
C	-3.571141	-1.826371	0.043126
C	-3.617092	-0.458931	-0.214260
C	-2.441828	0.249586	-0.479845
H	2.242021	-3.542092	0.217750
H	2.503042	1.314418	-0.676618
H	4.554181	0.085202	-0.213038
H	-4.554189	0.085324	-0.212999
H	-2.503021	1.314486	-0.676587
C	0.000019	1.700134	-0.949449
C	0.000046	2.553258	0.195784
C	0.000022	2.221304	-2.218043
C	0.000049	2.050088	1.524312
C	0.000071	3.961621	-0.012234
C	0.000049	3.622399	-2.417161
H	0.000004	1.545489	-3.067866
C	0.000075	2.910564	2.594560
H	0.000031	0.977191	1.689455
C	0.000098	4.824684	1.117563
C	0.000072	4.471424	-1.339611
H	0.000052	4.017190	-3.428136
C	0.000099	4.312289	2.391007
H	0.000078	2.515725	3.606007
H	-4.701903	-0.000522	1.784407
H	0.000117	5.898842	0.952867
H	0.000092	5.548202	-1.485693
H	0.000120	4.979208	3.247812
N	-0.000004	0.281472	-0.778067
O	-0.000040	-2.478182	-0.303453
S	-4.993401	-2.833727	0.396632
S	4.993313	-2.833850	0.396612
C	-6.361511	-1.652456	0.348050
H	-7.261016	-2.232156	0.568620
H	-6.248265	-0.876812	1.109028
H	-6.468090	-1.201763	-0.641405
C	6.361425	-1.652578	0.348088
H	7.260926	-2.232294	0.568634
H	6.468006	-1.201837	-0.641345
H	6.248179	-0.876970	1.109104

39

1 Eopt -977.102627

C	2.514837	-3.565014	-0.350813
C	3.175484	-2.334332	-0.368683
C	2.487856	-1.176178	-0.048692
C	1.125146	-1.205690	0.283294
C	0.472911	-2.439646	0.291936
C	1.166090	-3.611585	-0.017662
C	1.124850	1.205743	0.283415
C	2.487562	1.176611	-0.048576
C	3.174884	2.334974	-0.368463
H	4.228679	2.264474	-0.619630
C	2.513907	3.565479	-0.350485
C	1.165140	3.611660	-0.017353
C	0.472273	2.439509	0.292142
H	4.229261	-2.263534	-0.619841
H	-0.579621	-2.487636	0.549301
H	0.637117	-4.559216	0.002345
H	0.635909	4.559145	0.002722
H	-0.580283	2.487179	0.549489
C	-0.919338	-0.000196	0.897480
C	-1.873732	-0.000117	-0.164513
C	-1.321881	-0.000318	2.208541
C	-1.494504	0.000092	-1.533548
C	-3.257137	-0.000262	0.172057
C	-2.698624	-0.000438	2.535538
H	-0.570919	-0.000336	2.992823
C	-2.449677	0.000114	-2.520212
H	-0.441046	0.000213	-1.795841
C	-4.220296	-0.000223	-0.873764
C	-3.642986	-0.000428	1.540477
H	-2.999080	-0.000550	3.578446
C	-3.826834	-0.000047	-2.188790
H	-2.149351	0.000258	-3.563633
H	-5.274938	-0.000338	-0.611751
H	-4.569515	-0.000023	-2.980897

N	0.478139	-0.000061	0.597902
O	3.202331	0.000305	-0.023051
H	3.057827	-4.472328	-0.594594
H	3.056655	4.472958	-0.594188

61

1a Eopt -2235.425672

C	3.371641	-0.494793	1.161722
C	2.316759	-1.279746	0.679330
C	1.247984	-0.679023	0.043311
C	1.180191	0.714836	-0.113412
C	2.228386	1.487531	0.385942
C	3.319849	0.887493	1.012827
C	-1.033215	0.456835	-1.028339
C	-0.917129	-0.930472	-0.843679
C	-1.980016	-1.778935	-1.084111
H	-1.847485	-2.844347	-0.925520
C	-3.202596	-1.257220	-1.525257
C	-3.329446	0.113636	-1.725314
C	-2.252583	0.963917	-1.476603
H	2.327686	-2.360394	0.779302
H	2.201302	2.565895	0.277392
H	4.130626	1.508843	1.378590
H	-4.270350	0.533050	-2.065930
H	-2.367293	2.030363	-1.634351
C	-0.046521	2.693706	-0.875029
C	-0.588859	3.445051	0.210087
C	0.364743	3.307978	-2.029757
C	-1.023938	2.840046	1.419529
C	-0.693505	4.857201	0.062857
C	0.257897	4.712023	-2.167728
H	0.772483	2.705150	-2.835486
C	-1.539753	3.608080	2.434327
H	-0.945481	1.763678	1.538086
C	-1.231989	5.622637	1.133011
C	-0.259038	5.467287	-1.145621
H	0.587750	5.184046	-3.087611
C	-1.645723	5.013300	2.291342
H	-1.869656	3.136425	3.354997
H	-1.310035	6.700157	1.015885
H	-0.344304	6.545905	-1.245907
H	-2.055918	5.605935	3.103318
N	0.071056	1.272658	-0.758578
O	0.270532	-1.505214	-0.458087
C	-5.265625	-2.610502	-0.218054
C	-6.220088	-3.627302	-0.096226
C	-4.922048	-1.845305	0.898962
C	-6.825857	-3.870672	1.133761
H	-6.489365	-4.232258	-0.958170
C	-5.529255	-2.102779	2.128028
H	-4.186565	-1.051130	0.820331
C	-6.482269	-3.111594	2.252730
H	-7.564809	-4.662277	1.216228
H	-5.252767	-1.503769	2.990836
C	5.782228	-1.801262	0.618359
C	5.566218	-1.425384	-0.709350
C	6.867007	-2.626392	0.938702
C	6.430487	-1.877937	-1.706471
H	4.731448	-0.784362	-0.973801
C	7.727175	-3.067024	-0.063593
H	7.040333	-2.928725	1.968336
C	7.512804	-2.696824	-1.391582
H	6.251807	-1.581816	-2.736083
H	8.565220	-3.706648	0.197164
H	8.182046	-3.045001	-2.172241
H	-6.952295	-3.306058	3.211816
S	-4.572061	-2.348239	-1.846367
S	4.753341	-1.263629	1.979451

69

1d_triplet Eopt -1818.364162

C	2.414441	-1.815331	-0.167305
C	1.027633	-1.933119	-0.114527
C	0.256100	-0.794279	-0.252719
C	0.837422	0.475595	-0.449236
C	2.238779	0.565074	-0.499737
C	3.021923	-0.560502	-0.361110
C	-1.341859	1.430192	-0.494600
C	-1.887774	0.145261	-0.304779
C	-3.256159	-0.067692	-0.219706
H	-3.628249	-1.074864	-0.074824
C	-4.102935	1.030322	-0.320414
C	-3.582764	2.328762	-0.500528
C	-2.226619	2.524153	-0.586463
H	0.555709	-2.897743	0.034028
H	2.698444	1.534851	-0.649206
H	4.100505	-0.469555	-0.401126
H	-4.267383	3.166611	-0.570555
H	-1.820925	3.519017	-0.725753
C	0.602656	2.891360	-0.771166
C	0.954624	3.645459	0.381037
C	0.790731	3.353996	-2.092229
C	0.768598	3.195683	1.707127
C	1.538686	4.948894	0.153951
C	1.350467	4.605932	-2.285834
H	0.497128	2.724793	-2.927874
C	1.144180	3.995593	2.801576
H	0.328751	2.217566	1.884895
C	1.901317	5.720885	1.281936
C	1.723555	5.400789	-1.185286
H	1.502544	4.977082	-3.296074
C	1.706881	5.248669	2.590219
H	0.991220	3.625831	3.812282
H	2.341074	6.702538	1.118805

H	2.162687	6.382260	-1.346881
H	1.997654	5.867904	3.435154
N	0.025903	1.586325	-0.577651
O	-1.091814	-0.947220	-0.194422
C	-6.039543	-0.311507	-0.129421
C	-6.324038	-1.044644	-1.278515
C	-6.381243	-0.786345	1.125454
C	-6.954542	-2.274187	-1.159982
H	-6.050172	-0.655000	-2.254063
C	-7.018188	-2.022848	1.249434
H	-6.152032	-0.198928	2.009083
C	-7.303917	-2.771348	0.103703
H	-7.186595	-2.862329	-2.042029
H	-7.282073	-2.380110	2.237652
C	4.500694	-2.929774	-0.055470
C	5.208498	-2.755120	1.122192
C	5.157834	-3.127569	-1.266481
C	6.604256	-2.770110	1.101537

61

1c_triplet Eopt -1589.373735

C	-0.742586	-3.520485	-0.237515
C	-1.319685	-2.258525	-0.162702
C	-0.494102	-1.149404	-0.281825
C	0.894677	-1.268172	-0.479604
C	1.450252	-2.561827	-0.545748
C	0.646386	-3.669014	-0.427253
C	1.061193	1.105592	-0.499577
C	-0.329304	1.185316	-0.296100
C	-0.990416	2.400735	-0.188490
H	-2.062727	2.408426	-0.033653
C	-0.242315	3.568094	-0.282027
C	1.152188	3.517924	-0.482824
C	1.791726	2.307345	-0.589801
H	-2.382910	-2.113741	-0.013215
H	2.518356	-2.669797	-0.691242
H	1.068757	-4.666307	-0.476872
H	1.710060	4.445138	-0.549720
H	2.863021	2.261761	-0.744944
C	3.091966	-0.232771	-0.784592
C	3.920596	-0.242663	0.370421
C	3.591739	-0.323986	-2.102300
C	3.433483	-0.154898	1.693416
C	5.345831	-0.349413	0.149744
C	4.960267	-0.426003	-2.289850
H	2.900488	-0.312266	-2.940402
C	4.313243	-0.169411	2.790976
H	2.363321	-0.075783	1.867446
C	6.195003	-0.360784	1.280568
C	5.834499	-0.439591	-1.186263
H	5.361613	-0.496398	-3.297538

H	4.677984	-2.603061	2.057055
C	6.544780	-3.144312	-1.291841
H	4.586102	-3.265007	-2.178919
C	7.275867	-2.964035	-0.109413
H	7.144519	-2.631221	2.030330
H	7.077553	-3.298270	-2.224722
O	-5.449999	0.944990	-0.248337
O	3.108740	-2.967710	-0.021781
O	8.624519	-2.995142	-0.237763
C	9.414292	-2.821277	0.931584
H	9.223519	-3.615835	1.661825
H	10.451812	-2.876642	0.601577
H	9.232486	-1.843901	1.392672
O	-7.914896	-3.980752	0.113827
C	-8.294055	-4.531147	1.368640
H	-7.421696	-4.692431	2.011940
H	-9.018513	-3.888691	1.881765
H	-8.759262	-5.491301	1.144558

C	5.684011	-0.271389	2.585664
H	3.912665	-0.100023	3.799122
H	7.268346	-0.441238	1.122039
H	6.907453	-0.519970	-1.343072
H	6.365249	-0.282467	3.432822
N	1.668322	-0.130745	-0.595672
O	-1.089893	0.066361	-0.197801
C	-2.145425	4.939659	0.016932
C	-2.624557	5.011182	1.319358
C	-2.986769	5.050498	-1.083367
C	-3.991526	5.195214	1.521468
H	-1.937313	4.922024	2.154848
C	-4.351759	5.234420	-0.867696
H	-2.577121	4.991436	-2.086824
C	-4.854968	5.305823	0.431384
H	-4.379580	5.254083	2.533608
H	-5.020821	5.323181	-1.717982
C	-2.821737	-4.608899	0.057189
C	-3.658465	-4.612511	-1.052152
C	-3.319919	-4.600131	1.354328
C	-5.037959	-4.602530	-0.851234
H	-3.233805	-4.621101	-2.051083
C	-4.701272	-4.590199	1.541705
H	-2.635933	-4.599085	2.197244
C	-5.559978	-4.590446	0.442325
H	-5.703647	-4.606122	-1.708753
H	-5.104444	-4.584194	2.549602
H	-6.634864	-4.584090	0.593815
H	-5.918426	5.450586	0.594192
O	-0.776234	4.807879	-0.191924
O	-1.445361	-4.671906	-0.136162

63

3_triplet Eopt -1664.752901

C	-3.296704	1.432857	0.323972
C	-2.296618	0.589680	-0.057311
C	-0.972831	1.127992	-0.276530
C	-0.744012	2.558188	0.014030
C	-1.803555	3.376602	0.387124
C	-3.085817	2.848700	0.542880
C	1.583343	2.076856	-0.331107
C	1.322848	0.708989	-0.143698
C	2.345659	-0.240317	-0.181415
H	2.094188	-1.284369	-0.034025
C	3.644584	0.180011	-0.423260
C	3.923757	1.538125	-0.643974
C	2.906303	2.469846	-0.597513
H	-2.471491	-0.463857	-0.244394
H	-1.642353	4.440260	0.528927
H	-3.922144	3.465620	0.848368
H	4.943606	1.846682	-0.846655
H	3.134802	3.517141	-0.760024
C	0.847245	4.397763	-0.069506
C	0.621275	5.227932	-1.162934
C	1.355015	4.896068	1.127074
C	0.911522	6.586415	-1.054323
C	1.642791	6.255214	1.227074
H	1.520709	4.224191	1.963659
C	1.421407	7.099151	0.138394
H	2.037890	6.654251	2.155969
H	1.646878	8.158047	0.219915
N	0.543204	2.998065	-0.179246
O	0.064416	0.263037	0.061778
C	4.522265	-2.003867	-0.172109
C	4.564926	-2.409778	1.160703
C	4.344344	-2.929071	-1.188001
C	4.422301	-3.754118	1.470970
H	4.705837	-1.674369	1.946906
C	4.202671	-4.284743	-0.882101
H	4.313820	-2.596977	-2.221101
C	4.239299	-4.699534	0.451923
H	4.453724	-4.089016	2.502928
H	4.066320	-4.994295	-1.689567
C	-4.917121	-0.286887	0.353951
C	-4.810998	-1.149087	1.443172
C	-5.396951	-0.740343	-0.864335
C	-5.185923	-2.477566	1.302586
H	-4.435269	-0.778740	2.391956
C	-5.776975	-2.076310	-1.011873
H	-5.474316	-0.055477	-1.703169
C	-5.669324	-2.949380	0.074418
H	-5.112467	-3.163663	2.140424
H	-6.151396	-2.414186	-1.970886
O	4.714415	-0.667533	-0.490724
O	-4.593027	1.053195	0.507497
O	4.110609	-5.987698	0.856085
O	-6.009662	-4.262077	0.036731
C	3.913540	-6.984979	-0.137161
H	2.991467	-6.806721	-0.702172
H	4.764565	-7.034352	-0.826021
H	3.830043	-7.929654	0.400696
C	-6.514288	-4.787802	-1.183272
H	-7.441242	-4.284033	-1.480322
H	-5.775357	-4.705377	-1.988591
H	-6.722004	-5.840806	-0.991484
H	0.740247	7.242309	-1.902036
H	0.223645	4.809904	-2.082617

69

1b_triplet Eopt -2464.321909

C	0.501487	-3.558049	-0.372752
C	-0.149300	-2.332616	-0.293597
C	0.598785	-1.166405	-0.359388
C	1.998794	-1.187496	-0.509861
C	2.641469	-2.438542	-0.584916
C	1.907116	-3.598808	-0.517617
C	2.006078	1.190657	-0.472839
C	0.606269	1.173443	-0.320871
C	-0.134252	2.341652	-0.216340
H	-1.209830	2.266942	-0.103712
C	0.524165	3.564928	-0.257555
C	1.929676	3.601577	-0.404403
C	2.656423	2.439369	-0.510402
H	-1.224427	-2.254655	-0.178919
H	3.718578	-2.472665	-0.696873
H	2.420885	-4.553179	-0.576891
H	2.449497	4.554039	-0.434442
H	3.733445	2.470136	-0.624098
C	4.130049	-0.001336	-0.716478
C	4.920460	-0.012169	0.464788
C	4.675191	0.008574	-2.019261
C	4.387099	-0.023659	1.772609
C	6.355789	-0.010728	0.288450
C	6.052575	0.008950	-2.163885
H	4.010970	0.015938	-2.878960
C	5.231200	-0.032517	2.897847
H	3.309076	-0.025728	1.912915
C	7.167936	-0.019869	1.446097
C	6.890926	-0.000050	-1.032703
H	6.489018	0.016647	-3.159319
C	6.611435	-0.030508	2.735348
H	4.795690	-0.041033	3.893767
H	8.248698	-0.018609	1.321209
H	7.971207	0.000752	-1.156119
H	7.265021	-0.037234	3.604053
N	2.697125	0.000620	-0.572166

O	-0.080236	0.004562	-0.271339
C	-2.012408	4.662334	0.041080
C	-2.822730	4.520388	-1.084246
C	-2.556300	4.449112	1.314457
C	-4.162714	4.159125	-0.955908
H	-2.411229	4.683290	-2.075793
C	-3.886850	4.089583	1.452764
H	-1.934568	4.557389	2.197952
C	-4.698083	3.939298	0.318099
H	-4.768304	4.054101	-1.848066
H	-4.315488	3.920237	2.435325
C	-2.045523	-4.652005	-0.144356
C	-2.832074	-4.468922	-1.289212
C	-2.617241	-4.482326	1.115677
C	-4.165309	-4.112926	-1.169051
H	-2.398198	-4.599170	-2.275973
C	-3.957651	-4.123808	1.247419
H	-2.016500	-4.622231	2.009301
C	-4.736376	-3.936039	0.100083
H	-4.781427	-3.967511	-2.050574
H	-4.374186	-3.997080	2.239514
O	-5.978753	3.582882	0.552829
C	-6.847924	3.406715	-0.561025
H	-6.961699	4.338618	-1.125314
H	-6.486263	2.612502	-1.223149
H	-7.811738	3.117749	-0.142035
O	-6.040458	-3.587840	0.115259
C	-6.674589	-3.391693	1.374913
H	-6.664476	-4.311044	1.970543
H	-6.197524	-2.580275	1.935174
H	-7.705174	-3.118384	1.148571
S	-0.334205	-5.116246	-0.297861
S	-0.303104	5.124864	-0.137434

47

2_triplet Eopt -1851.961993

C	2.467399	-2.892217	-0.114489
C	1.087261	-3.026117	0.051874
C	0.275376	-1.911259	-0.054007
C	0.802771	-0.632836	-0.329300
C	2.191427	-0.516937	-0.509631
C	3.009294	-1.623676	-0.401535
C	-1.406318	0.256827	-0.253403
C	-1.898021	-1.042706	-0.005344
C	-3.251164	-1.297136	0.165958
H	-3.554706	-2.320419	0.353515
C	-4.155865	-0.240924	0.099675
C	-3.677921	1.070976	-0.126735
C	-2.336333	1.317354	-0.295354
H	0.625181	-3.987224	0.256471
H	2.613015	0.452543	-0.745352
H	4.075279	-1.495751	-0.546531
H	-4.377811	1.900615	-0.159521
H	-1.979538	2.327127	-0.455465
C	0.459779	1.763901	-0.723716
C	1.227320	2.450000	0.264833
C	0.144309	2.327333	-1.987830
C	1.503160	1.942727	1.550928
C	1.737779	3.752696	-0.098575
C	0.644562	3.574102	-2.306552
H	-0.456847	1.755564	-2.689318
C	2.274821	2.675001	2.468738
H	1.099130	0.977449	1.844457
C	2.509450	4.454633	0.852733
C	1.438065	4.283090	-1.386296
H	0.426478	4.009148	-3.278192
C	2.778843	3.920634	2.120356
H	2.472584	2.257355	3.452293
H	2.892375	5.436043	0.581642
H	1.827676	5.264490	-1.643861
H	3.378292	4.487354	2.827962
N	-0.049786	0.456875	-0.425570
O	-1.061696	-2.106062	0.094929
S	-5.890009	-0.424859	0.295853
S	3.430377	-4.356134	0.041187
C	-6.078444	-2.197194	0.605385
H	-7.149304	-2.361837	0.742220
H	-5.733878	-2.785406	-0.247699
H	-5.553286	-2.494529	1.515629
C	5.118054	-3.775813	-0.259836
H	5.749864	-4.662988	-0.178159
H	5.220150	-3.359206	-1.263774
H	5.427902	-3.048836	0.493314

61

1a_triplet Eopt -2235.325732

C	2.455877	-2.124290	-0.073141
C	1.067989	-2.248126	-0.020010
C	0.280096	-1.121900	-0.187471
C	0.845452	0.148701	-0.415318
C	2.246166	0.254096	-0.467430
C	3.037670	-0.860681	-0.299147
C	-1.343389	1.075922	-0.480690
C	-1.874145	-0.210024	-0.256264
C	-3.240147	-0.431462	-0.158290
H	-3.592035	-1.442525	0.012825
C	-4.109261	0.646662	-0.278457
C	-3.594250	1.945336	-0.496699
C	-2.240070	2.156453	-0.596382
H	0.581713	-3.203273	0.152858
H	2.695205	1.224714	-0.642058
H	4.114764	-0.750301	-0.342947
H	-4.269103	2.790765	-0.586599
H	-1.847310	3.152471	-0.762172

C	0.582413	2.550415	-0.807842
C	0.946032	3.336044	0.319525
C	0.744031	2.981231	-2.143269
C	0.783109	2.919202	1.659138
C	1.517632	4.636756	0.050012
C	1.291147	4.231758	-2.377134
H	0.441163	2.329371	-2.957955
C	1.171375	3.748594	2.726760
H	0.351409	1.943750	1.868799
C	1.893725	5.438985	1.152032
C	1.677566	5.056029	-1.303148
H	1.423457	4.578669	-3.398697
C	1.723078	4.999057	2.474696
H	1.036566	3.403832	3.748794
H	2.324584	6.418770	0.957108
H	2.107305	6.035903	-1.496397
H	2.023784	5.641023	3.298908
N	0.021338	1.245000	-0.572802
O	-1.065163	-1.290550	-0.124894
C	-6.114928	-1.239232	0.117181
C	-6.282126	-2.105632	-0.965931
C	-6.163970	-1.723888	1.426657
C	-6.490219	-3.463913	-0.734503
H	-6.243804	-1.722777	-1.981115
C	-6.372424	-3.083472	1.649525
H	-6.034621	-1.045112	2.264018
C	-6.534335	-3.952468	0.570908
H	-6.618105	-4.138877	-1.575041
H	-6.409038	-3.461622	2.666415
C	5.084241	-3.040524	0.102809
C	5.707179	-2.623722	1.282070
C	5.786084	-3.046631	-1.104906
C	7.034382	-2.201385	1.246924
H	5.157098	-2.623462	2.218034
C	7.114119	-2.624830	-1.131310
H	5.297013	-3.372440	-2.017720
C	7.736973	-2.201392	0.042117
H	7.518539	-1.874106	2.161760
H	7.660256	-2.627360	-2.069456
H	8.771567	-1.873018	0.018316
H	-6.697578	-5.011183	0.747973
S	3.392476	-3.611236	0.147005
S	-5.868934	0.505373	-0.172940

39

1_triplet Eopt -977.002087

C	2.706934	-3.470770	-0.347441
C	3.299189	-2.227041	-0.474290
C	2.538567	-1.089306	-0.219142
C	1.186007	-1.182666	0.169845
C	0.601636	-2.459243	0.287895
C	1.356830	-3.583322	0.031354
C	1.088367	1.196968	0.262411
C	2.439850	1.245812	-0.136155
C	3.101119	2.458795	-0.309075
H	4.141717	2.454781	-0.614266
C	2.409347	3.635930	-0.087194
C	1.060035	3.606808	0.309768
C	0.403446	2.407604	0.484451
H	4.336702	-2.114677	-0.769085
H	-0.438895	-2.535505	0.579430
H	0.902476	-4.563765	0.122748
H	0.528122	4.536275	0.480919
H	-0.635013	2.374638	0.791352
C	-0.914710	-0.100724	0.808725
C	-1.902785	-0.029620	-0.210780
C	-1.218348	-0.240723	2.181027
C	-1.611884	0.100228	-1.586779
C	-3.283287	-0.095160	0.214928
C	-2.547511	-0.304439	2.564629
H	-0.411479	-0.293684	2.906573
C	-2.643413	0.171013	-2.540423
H	-0.576669	0.146684	-1.915375
C	-4.289099	-0.021037	-0.776136
C	-3.574123	-0.230900	1.603993
H	-2.799181	-0.410439	3.616687
C	-3.972218	0.110837	-2.137779
H	-2.392802	0.272296	-3.593277
H	-5.329871	-0.069343	-0.463012
H	-4.614826	-0.280014	1.914923
H	-4.770481	0.165634	-2.873735
N	0.469486	-0.028551	0.417149
O	3.145794	0.112488	-0.362622
H	3.289726	-4.363890	-0.545376
H	2.914038	4.586689	-0.220570

8. B. G. McCarthy, R. M. Pearson, C. H. Lim, S. M. Sartor, N. H. Damrauer, G. M. Miyake, *J. Am. Chem. Soc.* **2018**, *140*, 5088.

11. R. M. Pearson, C. H. Lim, B. G. McCarthy, C. B. Musgrave, G. M. Miyake, *J. Am. Chem. Soc.* **2016,** *138*, 11399.

35. Frisch, M. J. *et al. Gaussian 16 Rev. C.01*, Wallingford, CT, 2016.

36. The PyMOL Molecular Graphics System, Version 2.0.7, Schrödinger, LLC

37. A. D. Becke, *J. Chem. Phys.* **1997**, *107*, 8554.

38. J.-D. Chai, M. Head-Gordon, *Phys. Chem. Chem. Phys.* **2008**, *10*, 6615.

39. V. A. Rassolov, M. A. Ratner, J. A. Pople, P. C. Redfern, L. A. Curtiss, *J. Comput. Chem.* **2001**, *22*, 976.

40. M. M. Francl, W. J. Pietro, W. J. Hehre, J. S. Binkley, M. S. Gordon, D. J. DeFrees, J. A. Pople, *J. Chem. Phys.* **1982**, *77*, 3654.

41. P. C. Hariharan, J. A. Pople, *Theor. Chim. Acta* **1973**, *28*, 213.

42. W. J. Hehre, R. Ditchfield, J. A. Pople, *J. Chem. Phys.* **1972**, *56*, 2257.

43. T. Clark, J. Chandrasekhar, G. W. Spitznagel, P. V. R. Schleyer, *J. Comput. Chem.* **1983**, *4*, 294.

44. Y. Zhao, D. G. Truhlar, *Theor. Chem. Acc.* **2008**, *120*, 215.

45. S. Grimme, J. Antony, S. Ehrlich, H. A Krieg, *J. Chem. Phys.* **2010**, *132*, 154104.

46. F. Weigend, R. Ahlrichs, *Phys. Chem. Chem. Phys.* **2005**, *7*, 3297.

47. A. Hellweg, D. Rappoport, *Phys. Chem. Chem. Phys.* **2015**, *17*, 1010.

48. F. Weigend, *Phys. Chem. Chem. Phys.* **2006**, *8*, 1057.

49. A. V. Marenich, C. J. Cramer, D. G. Truhlar, *J. Phys. Chem. B* **2009**, *113*, 6378.

50. B. Mennucci, E. Cancès, J. Tomasi, *J. Phys. Chem. B* **1997**, *101*, 10506.

51. G. Scalmani, M. J. Frisch, *J. Chem. Phys.* **2010**, *132*, 114110.

52. J. Tomasi, B. Mennucci, E. Cancès, *J. Mol. Struct. THEOCHEM* **1999**, *464*, 211.

53. B. Mennucci, J. Tomasi, *J. Chem. Phys.* **1997**, *106*, 5151.

54. G. Luchini, J. V. Alegre-Requena, I. Funes-Ardoiz, R. S. Paton, *F1000Research* **2020**, *9*, 291.

55. S. Grimme, *Chem. – Eur. J.* **2012**, *18*, 9955.
